# F‐Box and Leucine‐Rich Repeat Protein 4 (FBXL4) Maintains Sarcomere Integrity and Cardiac Function by Enhancing K48‐Linked Ubiquitinated Degradation of Profilin‐1 (PFN1)

**DOI:** 10.1002/advs.202516702

**Published:** 2026-01-27

**Authors:** Xingda Li, Xueqi He, Xinyuan Hao, Yu Zhang, Xin Zhao, Shuang Wang, Zhenru Wang, Haonan Du, Hongda Li, Lian Yi, Zhimin Du, Weijie Du

**Affiliations:** ^1^ Institute of Clinical Pharmacy the Second Affiliated Hospital of Harbin Medical University & State Key Laboratory of Frigid Zone Cardiovascular Disease, Harbin Medical University Harbin China; ^2^ Department of Cardiology the First Affiliated Hospital of Harbin Medical University Harbin Heilongjiang Province China; ^3^ State Key Laboratory of Quality Research in Chinese Medicines Macau University of Science and Technology Macau China; ^4^ State Key Laboratory of Frigid Zone Cardiovascular Diseases (SKLFZCD) Department of Pharmacology (State Key Laboratory ‐Province Key Laboratories of Biomedicine‐Pharmaceutics of China, Key Laboratory of Cardiovascular Research, College of Pharmacy, Ministry of Education) Harbin Medical University Harbin China; ^5^ Research Unit of Noninfectious Chronic Diseases in Frigid Zone (2019RU070) Chinese Academy of Medical Sciences Harbin China; ^6^ Northern Translational Medicine Research and Cooperation Center, Heilongjiang Academy of Medical Sciences Harbin Medical University Harbin China

**Keywords:** cardiac hypertrophy, FBXL4, PFN1, sarcomere remodeling, ubiquitin

## Abstract

Pathological cardiac hypertrophy is characterized by profound disruptions in protein turnover, a hallmark of maladaptive cardiac remodeling. This study aimed to elucidate the role and underlying molecular mechanisms of an FBP, F‐box and leucine‐rich repeat protein 4 (FBXL4), in pathological cardiac hypertrophy. Transcriptomic analysis of murine heart failure and human dilated cardiomyopathy samples revealed consistent downregulation of FBXL4. Similarly, FBXL4 expression was reduced in failing human hearts, hypertrophic mouse hearts, and angiotensin II (Ang II)‐treated neonatal mouse cardiomyocytes (NMCMs). Inducible ablation of FBXL4 in cardiomyocytes resulted in HF with reduced cardiac function, an enlarged heart chamber, increased fibrosis, and myofibrillar disorganization and sarcomere remodeling. Conversely, cardiac‐specific overexpression of FBXL4 attenuated pressure overload–induced hypertrophy. Mechanistically, FBXL4 interacts with PFN1 and promotes its K48‐linked ubiquitination at lysine 70, leading to its proteasomal degradation and the preservation of sarcomeric integrity. Restoration of FBXL4 expression via AAV9 delivery ameliorated cardiac hypertrophy and dysfunction in FBXL4‐iCKO mice, while AAV9‐mediated PFN1 knockdown or pharmacological inhibition partially reversed these phenotypes. Furthermore, the transcription factor SP1 was found to repress FBXL4 expression during hypertrophy. FBXL4 deficiency also induced hypertrophic features in hiPSC‐derived cardiomyocytes. Together, these findings establish FBXL4 as a key regulator of sarcomere integrity and cardiac function through ubiquitin‐mediated degradation of PFN1.

## Graphical abstract

Schematic diagram depicting the proposed signaling mechanisms underlying the effects of FBXL4 in the setting of cardiac hypertrophy. Under hypertrophic stimulation, cardiomyocytes‐specific overexpression FBXL4 maintains sarcomere integrity and cardiac function by enhancing K48‐linked ubiquitinated degradation of PFN1 at the K70 site.

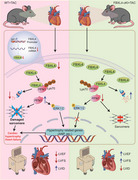

**Abbreviations**
AAV9: adeno‐associated virus serotype 9
Ang II: angiotensin II
cTnT: cardiomyocyte‐specific troponin‐T
DCM: dilated cardiomyopathy
FBPs: F‐box proteins
FBXL4: F‐box and leucine‐rich repeat protein 4
FBXL4‐cKI: cardiomyocyte‐specific FBXL4 knock‐in
FBXL4‐iCKO: inducible cardiomyocyte‐specific FBXL4 knockout
HCM: hypertrophic cardiomyopathy
HF: heart failure
HW/BW: heart weight‐to‐body weight
HW/TL: heart weight‐to‐tibia length
IF: immunofluorescence
LC/MS: liquid chromatography/mass spectrometry
LVIDd: left ventricular internal diameter diastolic
LVIDs: left ventricular internal diameter systolic
MTDPS13: mitochondrial DNA depletion syndrome 13
NMCMs: neonatal mouse ventricular cardiomyocytes
PFN1: profilin‐1
PTMs: post‐translational modifications
scRNA‐seq: single‐cell RNA sequencing
TAC: transaortic constriction
TEM: transmission electron microscope
UPS: ubiquitin–proteasome system

## 1 Introduction

Cardiac hypertrophy originates from the compensatory adaptation of the heart to various pathological injuries and is characterized by reactivated fetal genes, enlarged cardiomyocytes, enhanced protein synthesis, and disrupted sarcomere tissue structure, ultimately leading to heart failure (HF) [1, 2, 3]. Despite advances in pharmacological therapies (e.g., ACE inhibitors and beta‐blockers) [4, 5], the molecular mechanisms underlying pathological hypertrophy remain incompletely understood, highlighting the urgent need for novel therapeutic targets.

F‐box proteins (FBPs) function as integral subunits of the SKP1–CUL1–F‐box (SCF) ubiquitin ligase complex and play a critical role in substrate recognition followed by ubiquitin‐mediated degradation [6, 7]. These proteins directly interact with substrates via several structural domains, including leucine‐rich repeats, WD40 repeats, and additional motifs, facilitating the classification of FBPs into three subfamilies: FBXL, FBXW, and FBXO [8]. F‐box and leucine‐rich repeat protein 4 (FBXL4), a core SCF component, orchestrates protein interactions and contributes to mitochondrial dynamics, including mitochondrial fusion and calcium homeostasis [9, 10]. Pathogenic variants in FBXL4 cause mitochondrial DNA depletion syndrome 13 (MTDPS13), a disorder characterized by severe neurodegeneration, mitochondrial DNA depletion, and defective oxidative phosphorylation [11]. Recent studies have shown that FBXL4 limits excessive mitophagy by regulating the proteasomal degradation of the mitophagy receptors BNIP3 and NIX [12]. In addition, FBXL4 confers cardioprotection in HFpEF by modulating mitochondrial dynamics via Drp1 and enhancing SERCA2a function [13]. Furthermore, analysis of single‐cell RNA sequencing (scRNA‐seq) data from healthy and HF hearts revealed significant downregulation of FBXL4 expression in HF cardiomyocytes (GSE271946). Bulk RNA‐sequencing data from human and mouse heart samples (GSE46224 and GSE116250) further corroborated the decreased expression of FBXL4 in both dilated cardiomyopathy (DCM) and hypertrophic cardiomyopathy (HCM). Accordingly, we focused on the functional role and underlying molecular mechanism of FBXL4 in cardiac hypertrophy and HF.

Profilin‐1 (PFN1) is a highly conserved and multifunctional actin‐binding protein that plays a crucial role in catalyzing the nucleotide exchange of G‐actin and delivers ATP‐G‐actin to the growing barbed ends of F‐actin, which in turn promotes filament elongation [14, 15, 16]. Elevated PFN1 levels are observed in endothelial cells, vascular smooth muscle cells, and immune cells during cardiovascular stress, promoting endothelial dysfunction, inflammation, and plaque formation [17, 18, 19]. Recent studies have shown that ectopic PFN1 expression acts as a pathogenic driver in cardiomyocytes and is sufficient to promote hypertrophy, reactivate the fetal gene program, and induce sarcomeric disarray with filament elongation through ERK1/2 MAPK activation [20]. These findings position PFN1 as a potential therapeutic target for mitigating pathological cardiac remodeling in conditions such as heart failure and hypertrophic cardiomyopathy.

Therefore, in this study, we explored whether FBXL4 plays a critical role in stress‐induced cardiac hypertrophy and heart failure. FBXL4 functions as a previously unrecognized hypertrophy suppressor that attenuates cardiac dysfunction and sarcomeric disorganization through direct PFN1 binding and K48‐linked ubiquitin‐mediated degradation. This study provides new insights into the role and mechanism of FBXL4 in the treatment of cardiac hypertrophy‐related diseases.

## 2 Results

### 2.1 FBXL4 Expression Is Downregulated in Heart Failure Cardiac Tissue

**FIGURE 1:**
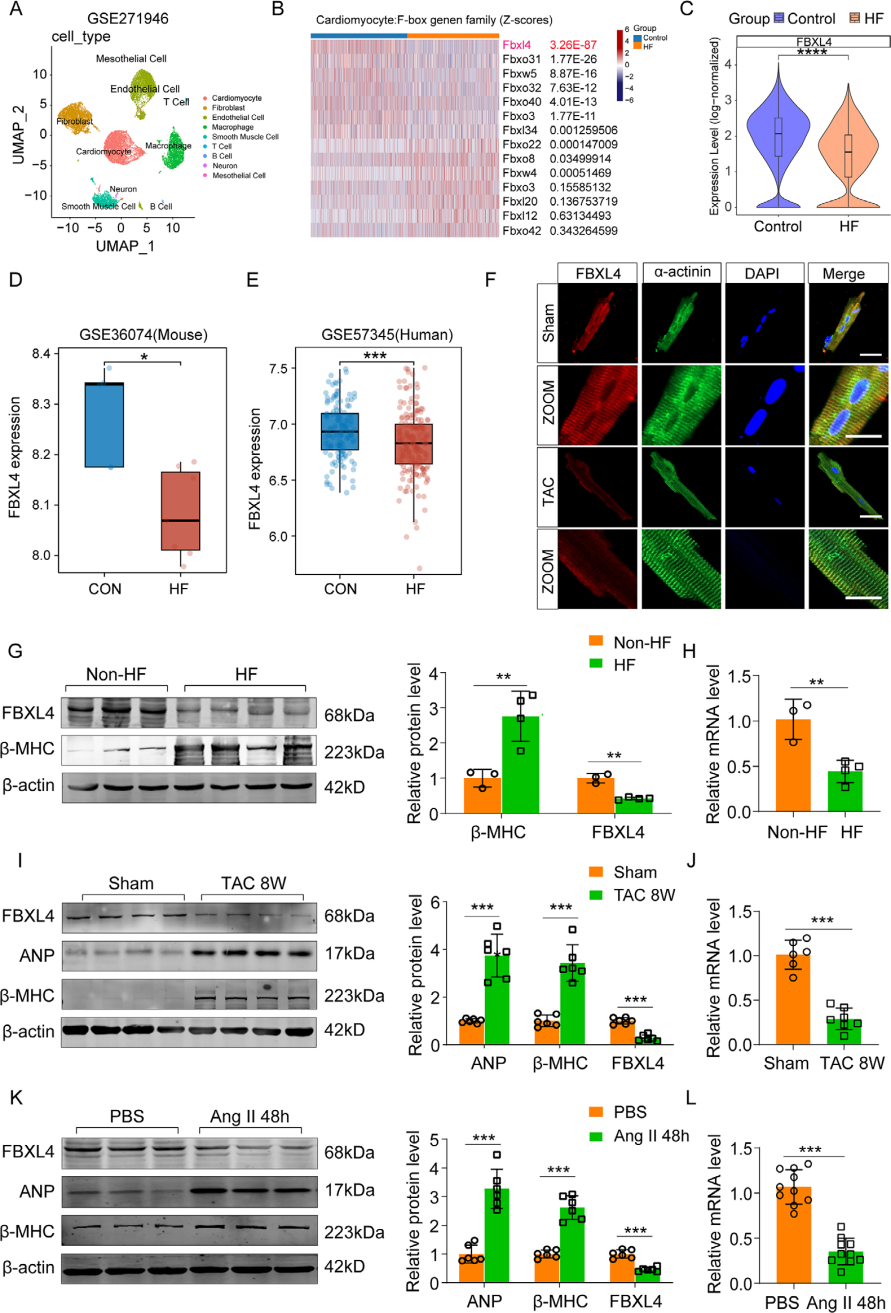
FBXL4 expression is down‐regulated in cardiac tissues of heart failure. (A) Uniform Manifold Approximation and Projection (UMAP) plot showing single cells isolated from the hearts of control and HF mice (from the GSE271946 database). (B) Heatmap showing the differentially regulated genes between control and HF. (C) The transcriptional level of FBXL4 in human control (*n* = 8) and HF (*n* = 60) from the GSE271946 database. (D) The transcriptional level of FBXL4 in mouse control (*n* = 4) and HF (*n* = 7) from the GSE36074 database. (E) The transcriptional level of FBXL4 in human control (*n* = 136) and HF (*n* = 176) from the GSE57345 database. (F) Immunofluorescence staining of adult cardiomyocytes from sham‐operated and TAC treated‐mice demonstrated cytoplasmic localization of FBXL4 with decreased expression post‐TAC surgery, stained with DAPI (blue) α‐actinin (green) and FBXL4 (red), *n* = 6, scale bar = 100 µm. Zoomed images show a higher magnification. Scale bar = 20 µm. (G, H) Western blot and qRT‐PCR analyses of FBXL4 expression in left ventricular tissues of human non‐HF subjects (*n* = 3) and patients with HF (HF, *n* = 4). (I) Protein levels of ANP, β‐MHC, and FBXL4 in left ventricular tissues of hypertrophic mice after 8‐weeks sham or TAC operation, *n* = 6 hearts/group. (J) mRNA levels of FBXL4 in the mouse left ventricle tissues at 8 weeks after sham or TAC operation, *n* = 6 mice/group. (K) Protein levels of ANP, β‐MHC, and FBXL4 in neonatal mouse cardiomyocytes (NMCMs) treated with either PBS as a control or angiotensin II (Ang II; 1 µm) for 48 h (*n* = 6/group). (L) mRNA levels of FBXL4 in NMCMs treated with Ang II (1 µm) for 48 h (*n* = 9/group). n represents the number of independent samples per group. Data are shown as mean ± SD. ^*^ *p* < 0.05, ^**^ *p* < 0.01, and ^***^ *p* < 0.001. Statistical differences were assessed by Wilcoxon rank‐sum test (B, C, D, E), Mann‐Whitney U Test (G, H, I, J, K, L).

Recent years have witnessed a surge of evidence for the involvement of FBPs in the process of maintaining cardiac homeostasis. To identify novel FBPs that are involved in the pathogenesis of cardiac hypertrophy and HF, we screened a single‐cell database of mouse HF derived from a sham surgery group and a transverse aortic constriction (TAC)‐induced HF model group (GSE271946). The cell types were divided into 7 clusters using Seurat, and we extracted cardiomyocyte subsets and compared the HF cardiomyocytes with control cardiomyocytes to analyze characteristic genes (Figure 1A and Figure S1). In this dataset, we filtered the top 20 F‐box proteins and identified F‐box/LRR‐repeat protein 4 (FBXL4) as the most differentially expressed (Figure 1B). FBXL4 was markedly downregulated in heart failure models (Figure 1C). To clarify the expression profile of the F‐box family in the heart, we scanned RNA‐sequencing data from mouse and human heart samples from the GSE36074 and GSE57345 datasets. We found that the expression of FBXL4 decreased coincidently during HF (Figure 1D,E), which may play important roles in myocardial hypertrophy. Therefore, we focused on the functional role of FBXL4 in hypertrophy.

We next assessed FBXL4 expression in pathological cardiac hypertrophy. Immunofluorescence (IF) staining revealed a weaker intensity of striated FBXL4 signal colocalization with α‐actinin in TAC‐subjected adult mouse cardiomyocytes than in sham control cardiomyocytes (Figure 1F), which implies that the protein is recurrently associated with sarcomeres. We subsequently compared the expression level of FBXL4 in left ventricular tissues from patients with heart failure (HF) and non‐HF subjects. The failed hearts were from patients with dilated cardiomyopathy. The results revealed that the protein and mRNA expression levels of FBXL4 were significantly lower in HF hearts than in non‐HF hearts (Figure 1G,H). A similar expression profile was observed in murine hearts when hypertrophy was induced in wild‐type (WT) mice by TAC for 8 weeks. As shown in Figure 1I,J, the expression level of FBXL4 was inversely correlated with those of the hypertrophic markers natriuretic peptide A (ANP) and β‐myosin heavy chain (β‐MHC) after pressure overload. In parallel, when hypertrophy was induced in isolated neonatal mouse ventricular cardiomyocytes (NMCMs) by Ang II stimulation, FBXL4 expression was strongly downregulated, which was reminiscent of that observed in human and mouse hearts (Figure 1K,L). These in vivo and in vitro results suggest that FBXL4 may play a regulatory role in the process of cardiac hypertrophy.

### 2.2 Inducible Cardiomyocyte‐Specific Deletion of FBXL4 in an Adult Heart Leads to the Development of HF

To explore the role of FBXL4 in cardiac hypertrophy, we engineered a FBXL4‐conditional allele, *FBXL4* ^flox/flox^, by targeting exon 4. We engineered an inducible cardiomyocyte‐specific *FBXL4* knockout model in which *FBXL4* ^flox/flox^ mice were interbred with Myh6‐MerCreMer mice, yielding iCKO animals (Figure S2A,B). At 8 weeks of age, the mice received intraperitoneal tamoxifen (20 mg/kg/day) for five consecutive days. Compared with control mice, iCKO mice presented markedly reduced levels of FBXL4 protein and mRNA expression at 7 days post‐induction (Figure S2C,D), whereas tamoxifen‐treated wild‐type mice maintained normal cardiac function and morphology at 28 days (Figure S2E–I).

**FIGURE 2:**
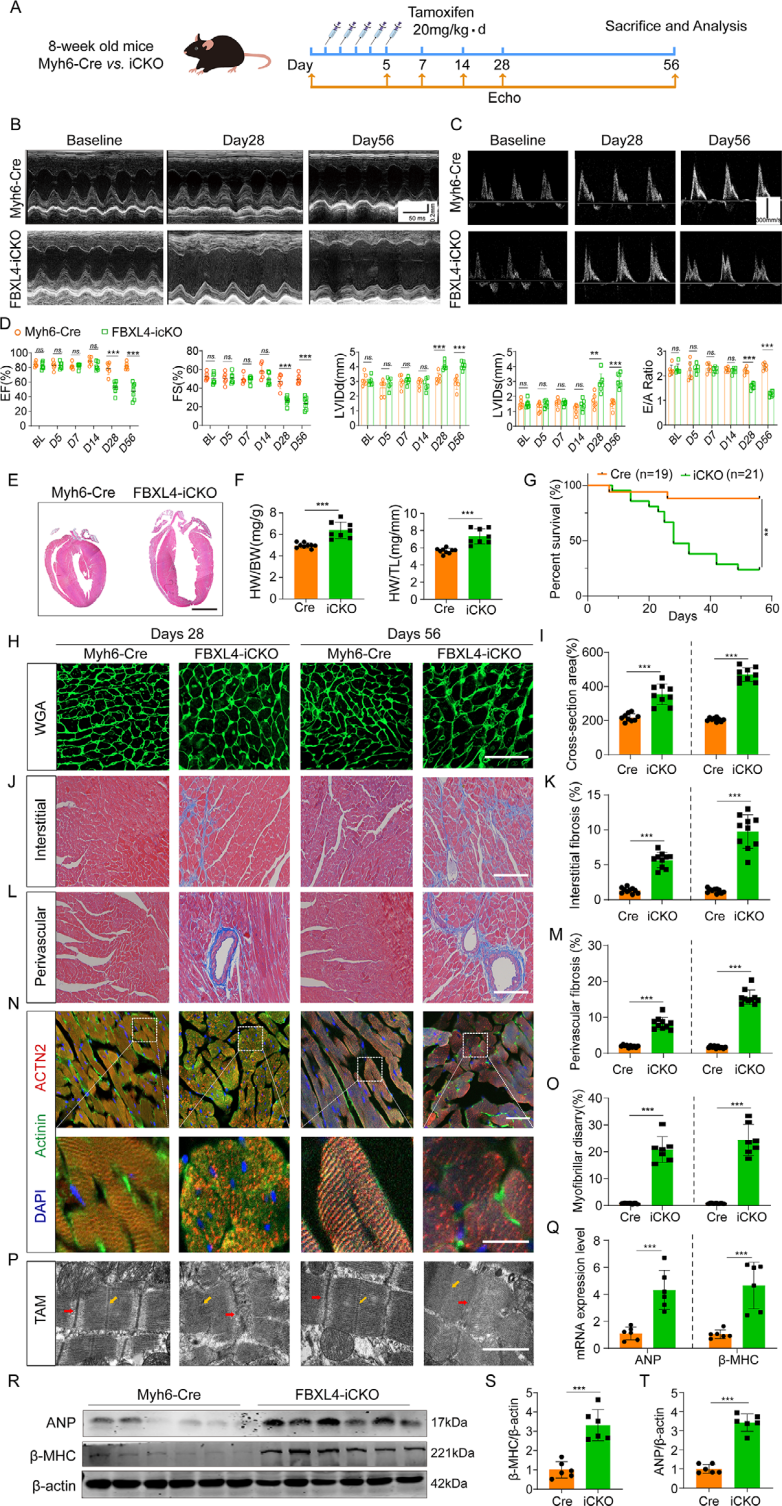
Inducible cardiomyocyte‐specific deletion of FBXL4 in an adult heart develops HF. (A) Schematic of tamoxifen injection and the echocardiography experiments in adult mouse hearts. Echo, echocardiography. (B, C) Representative echocardiographic images of M‐mode and mitral valve pulse‐wave Doppler in CTR vs. iCKO mice at baseline, day 28, and day 56. (D) Echo parameters of CTR vs. iCKO mice at baseline (BL), day 5, day 7, day 14, day 28, and day 56 were calculated (*n* = 6–8/group). (E) Representative 4‐chamber H&E images on day 56. Scale bar = 2 mm. (F) Heart weight normalized body weight (HW/BW) and heart weight normalized to tibia length (HW/TL) of CTR vs. iCKO on day 56 (*n* = 8/group). (G) Survival curve of Myh6‐Cre vs. FBXL4‐iCKO mice (*n* = 19 for Myh6‐Cre, *n* = 21 for iCKO). H, I) Representative WGA staining images and quantitation of cardiomyocyte size on days 28 and 56 (*n* = 300 cells/group). Scale bar = 100 µm. (J, K) Representative trichrome staining images and quantitation of cardiac interstitial fibrosis on days 28 and 56 (*n* = 5/group). Scale bar = 100 µm. (L, M) Representative trichrome staining images and quantitation of cardiac perivascular fibrosis on days 28 and 56 (*n* = 5/group), Scale bar = 100 µm. (N, O) Representative IF images and quantitation of Z‐disc organization and F‐actin architecture in FBXL4‐cKI mice following TAC‐induced hypertrophy, stained with DAPI (blue), ACTN2 (red), and α‐actinin (green). Three sections/mouse, *n* = 4 mice. Scale bars: upper, 100 µm; lower, 20 µm. (P) Representative transmission electron microscopy (TEM) analysis of sarcomere structure in Myh6‐Cre vs. iCKO on day 56. n = 3/group. The red and yellow arrows indicate Z‐disc and M‐band, respectively. Scale bars, 20 µm. Q) RT‐qPCR analysis showing ANP and β‐MHC expression in Myh6‐Cre vs. iCKO mice on day 56 (n = 5/group). (R–T) Western blot analysis of ANP and β‐MHC protein expression in Myh6‐Cre vs. iCKO mice on day 56 (n = 6/group). *n* represents the number of independent samples per group. Data are shown as mean ± SD. *ns*. indicates no significance. ^*^ *p* < 0.05, ^**^ *p* < 0.01, and ^***^ *p* < 0.001. Statistical differences were assessed by two‐way ANOVA with Sidak's multiple comparisons test (D), Kaplan–Meier analysis with the log‐rank Mantel‐Cox test (G), unpaired t test with Welch's correction (F, I, K, M, O, Q, S, T).

Serial measurements of cardiac function by echocardiography revealed that FBXL4‐iCKO mice manifested progressive contractile dysfunction, as evidenced by an impaired ejection fraction (EF%), fractional shortening (FS%), and an altered E/A ratio. The left ventricular internal diameters in both the diastolic (LVIDd) and systolic (LVID) stages increased significantly, indicating that the heart chambers were larger in the FBXL4‐iCKO mice than in the control mice (Figure 2A–D). Consistent with the echocardiography results, morphological analysis revealed significant enlargements in heart size and heart chamber size and thinning of the ventricle walls in iCKO mice (Figure 2E), along with elevated heart weight‐to‐body weight (HW/BW) and heart weight‐to‐tibia length (HW/TL) ratios (Figure 2F). Compared with Myh6‐Cre mice, FBXL4‐iCKO mice exhibited significantly reduced survival, with mortality onset at 1 week and markedly decreased survival rates by 4 weeks (Figure 2G). Histological analysis revealed a marked increase in the cross‐sectional area of cardiomyocytes and significant increases in both interstitial fibrosis and perivascular fibrosis in iCKO mice (Figure 2H–M). Numerous studies have demonstrated that sarcomeres function as the fundamental structural and contractile units of cardiomyocytes and play a pivotal role in the pathophysiology of cardiac hypertrophy and heart failure [21, 22]. IF revealed that FBXL4 deficiency led to apparent cytoskeleton reorganization, as evidenced by the disordered sarcomere structure and shortened sarcomere length (Figure 2N,O). Moreover, ultrastructural examination of the myocardium by transmission electron microscopy (TEM) revealed that the tight lateral arrangement of myofibrils and the aligned Z‐lines of adjacent myofibrils were disrupted in the cardiomyocytes of FBXL4‐iCKO mice (Figure 2P). Consistent with pathological hypertrophy, the protein and mRNA expression levels of atrial natriuretic peptide (ANP) and β‐myosin heavy chain (β‐MHC) were significantly increased in the FBXL4‐iCKO myocardium (Figure 2Q–T). Collectively, these findings establish that the cardiomyocyte‐specific ablation of FBXL4 in adulthood triggers sarcomeric disarray and promotes heart failure via both structural and molecular remodeling.

**FIGURE 3:**
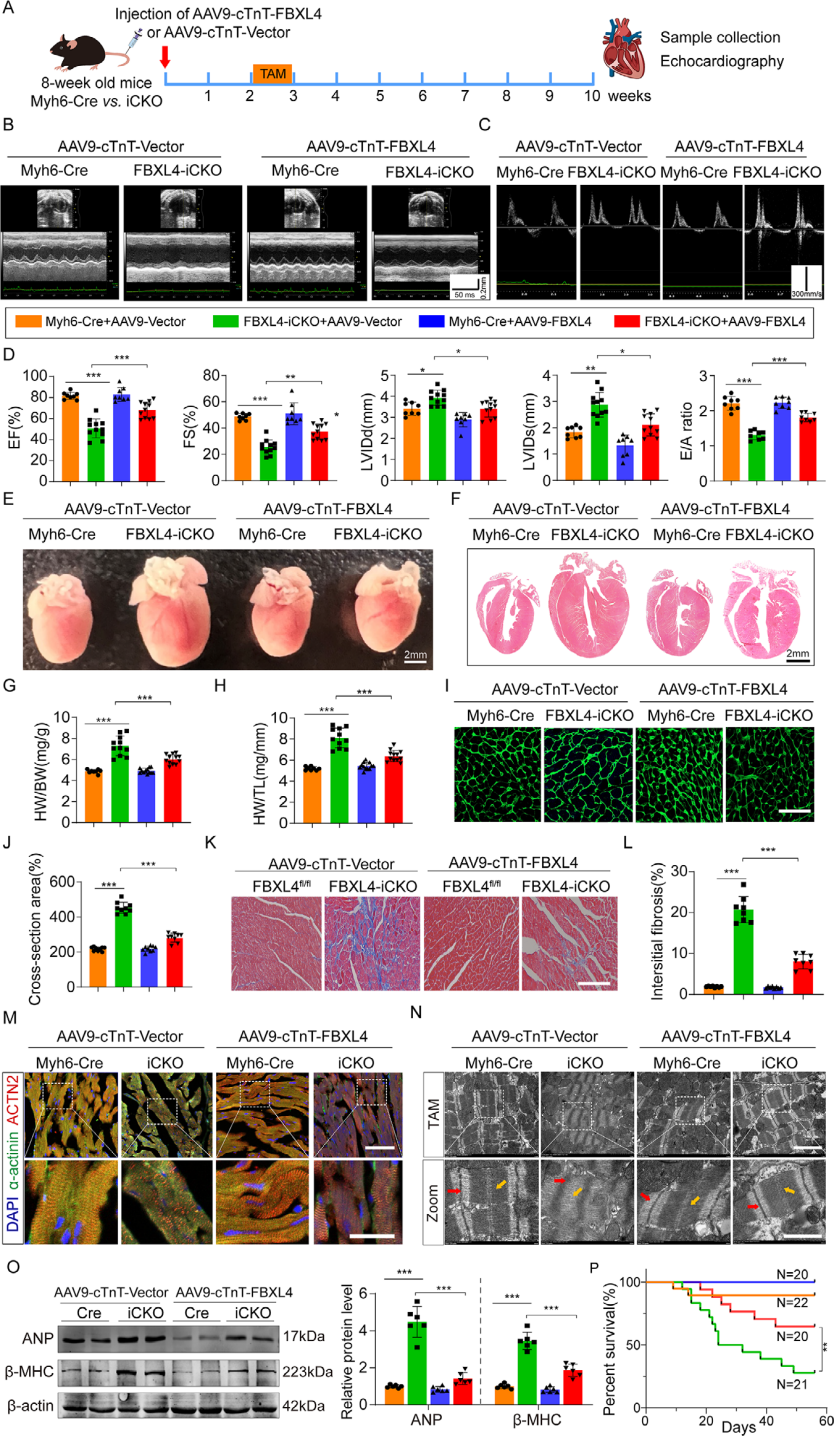
Cardiac‐specific FBXL4 overexpression restore FBXL4‐iCKO induced HF. (A) Schematic diagram of tail intravenous AAV9‐cTnT‐FBXL4/Vector and tamoxifen intraperitoneal injection. (B–D) Representative M‐mode and doppler echocardiography of the left ventricle and statistical data of EF%, FS%, LVIDd, LVIDs, and E/A ratio. *n* = 8–11. (E, F) Gross morphology and HE staining of the hearts (scale bar = 2 mm). G, H) Heart weight (HW)/body weight (BW) and HW/tibia length (TL) ratios in the different groups, *n* = 8–12/group. (I, J) Representative images and statistics of WGA staining (2–3 sections/mouse, Scale bar = 100 µm, *n* = 4). (K, L) Masson's trichrome staining (3 sections/mouse, Scale bar = 100 µm, *n* = 4 mice). (M) Representative IF images and statistics of Z‐disc organization and F‐actin architecture in AAV9‐Vector or AAV9‐FBXL4 treated with FBXL4‐iCKO mice, stained with DAPI (blue), ACTN2 (red), and α‐actinin (green), 3 sections/mouse, *n* = 4 mice. Scale bar = 100 µm. Scale bars: upper, 100 µm; lower, 20 µm. (N) Representative TEM analysis of sarcomere structure in AAV9‐Vector or AAV9‐FBXL4 treated with FBXL4‐iCKO mice. The red and yellow arrows indicate Z‐disc and M‐band, respectively. Scale bars, 100 and 20 µm. (O) Representative western blot analysis and summary data of ANP and β‐MHC and in cardiac tissues, *n* = 6. (P) Survival curve of individual experimental groups. (Myh6‐Cre+AAV9‐cTnT‐Vector, *n* = 22; FBXL4‐iCKO+AAV9‐cTnT‐Vector, *n* = 21; Myh6‐Cre+AAV9‐cTnT‐FBXL4, *n* = 20; FBXL4‐iCKO+ AAV9‐cTnT‐FBXL4, *n* = 20). n represents the number of independent samples per group. Data are shown as mean ± SD. *ns*. indicates no significance. ^*^ *p* < 0.05, ^**^ *p* < 0.01, and ^***^ *p* < 0.001. Statistical differences were assessed by one‐way ANOVA followed by Sidak post hoc multiple comparisons test (D, G H, J, L, O), Kaplan–Meier analysis with the log‐rank Mantel‐Cox test (P).

To determine whether FBXL4 deletion is specific to pathological cardiac hypertrophy, we employed a murine ramp swimming model of exercise‐induced physiological hypertrophy (Figure S3A) [23]. After three weeks of training, we confirmed the expected upregulation of the cardioprotective gene HSP70—a marker specific to physiological rather than pathological hypertrophy [24]—whereas both FBXL4 mRNA and protein expression levels remained unaltered (Figure S3B–D). Furthermore, echocardiography revealed progressive deterioration of cardiac function specifically in FBXL4‐iCKO mice (Figure S3E and Figure 3F), supporting the conclusion that loss of FBXL4 is specific to pathological cardiac remodeling.

### 2.3 Cardiac‐Specific FBXL4 Overexpression Restored FBXL4‐iCKO‐Induced HF

To further evaluate the impact of FBXL4 on HF, we constructed AAV9 vectors encoding FBXL4 (AAV9‐FBXL4) or a negative control (AAV9‐Vector) under the murine cardiac troponin‐T (cTnT) core promoter. The overexpression of FBXL4 by AAV9‐FBXL4 was first confirmed by the significant increases in FBXL4 protein and mRNA expression levels in FBXL4‐iCKO hearts (Figure S4A,B). Cardiomyocytes from adult mice also exhibited significant upregulation of FBXL4 expression (Figure S4C). The AAV9‐FBXL4 virus was delivered via tail vein injection 14 days before tamoxifen‐induced FBXL4 knockout, thereby preceding the onset of the phenotypic manifestations (Figure 3A). As illustrated in Figure 3B through 3D, FBXL4 overexpression strongly alleviated the impairment of cardiac function in FBXL4‐iCKO mice, as reflected by the significant restoration of the EF%, FS%, and E/A ratio, along with the amelioration of the increases in LVIDs and LVIDd. By comparison, AAV9‐Vector did not produce any appreciable effects on FBXL4 deletion‐induced cardiac dysfunction. The increases in the gross size of the heart and the HW/BW and HW/TL ratios in the FBXL4‐iCKO mice were markedly attenuated by AAV9‐FBXL4 but not by AAV9‐Vector (Figure 3E–H). Similarly, the application of AAV9‐FBXL4 abolished the FBXL4 deficiency‐induced increases in cardiac cells and fibrosis (Figure 3I–L). As expected, IF staining and TEM also revealed that FBXL4 overexpression significantly mitigated sarcomere remodeling in the FBXL4‐iCKO mice (Figure 3M,N). Moreover, we found that AAV9‐FBXL4 protected against FBXL4‐iCKO‐induced cardiomyocyte hypertrophy, as revealed by decreases in ANP and β‐MHC levels (Figure 3O). More strikingly, the mortality of FBXL4‐iCKO mice injected with AAV9‐FBXL4 was significantly lower than that of mice injected with control AAV9‐Vector (Figure 3P). These results indicate that FBXL4 replacement can relieve FBXL4 deficiency‐induced cardiac hypertrophy and heart failure.

### 2.4 Cardiomyocyte‐Specific FBXL4 Overexpression Alleviates Cardiac Hypertrophy and Dysfunction

**FIGURE 4:**
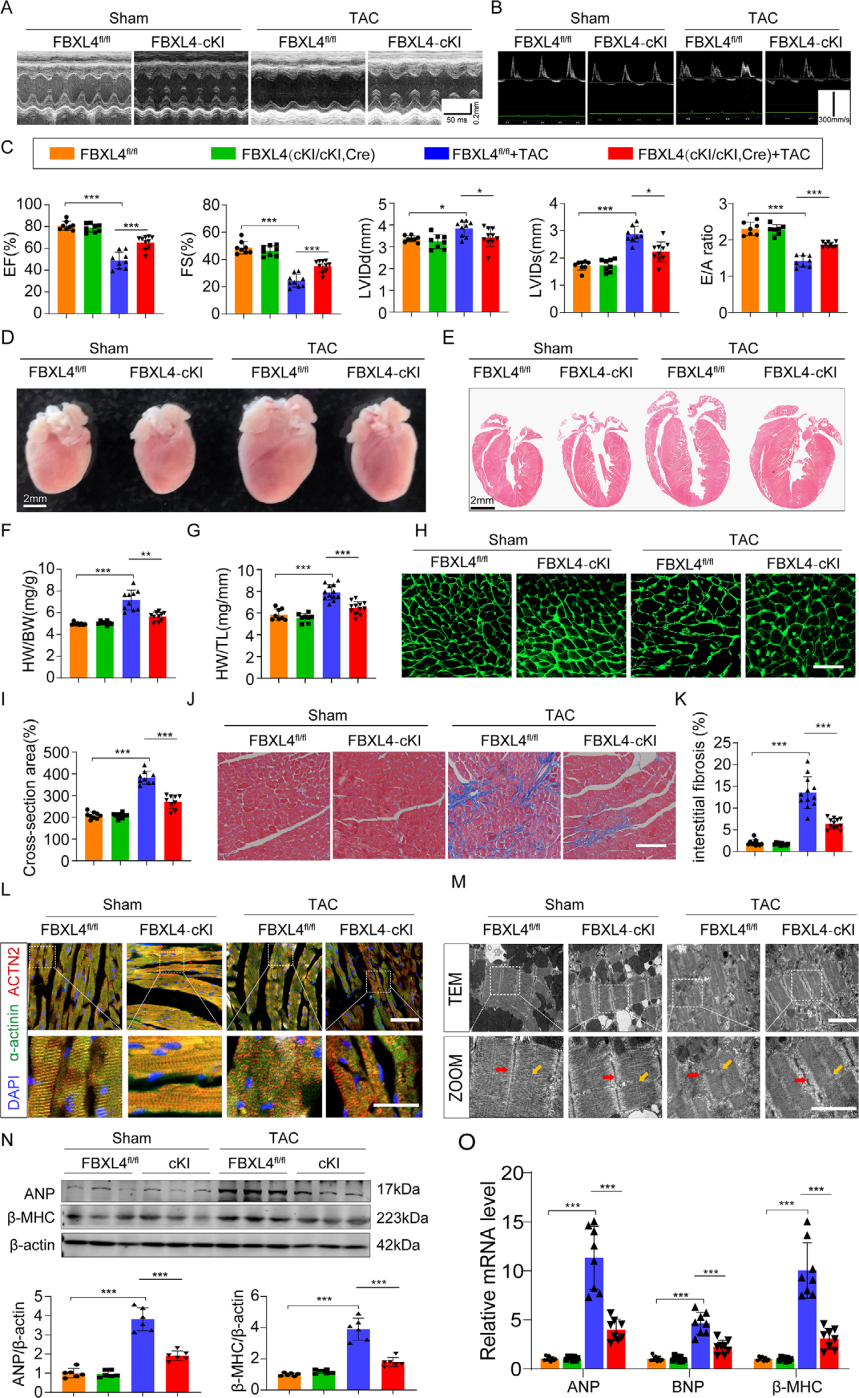
Cardiomyocytes‐specific FBXL4 overexpression alleviates cardiac hypertrophy and dysfunction. (A–C) Representative M‐mode echocardiography and doppler echocardiography of the left ventricle and statistical data of EF%, FS%, LVIDd, LVIDs and E/A ratio *n* = 8–10/group. (D, E) Gross morphology and HE staining of the hearts (scale bar = 2 mm). (F, G) Heart weight (HW)/body weight (BW) and HW/tibia length (TL) ratios in the different groups, *n* = 8–12/group. (H, I) Representative images and statistics of WGA staining (2‐3 sections/mouse, Scale bar = 100 µm, *n* = 4). (J, K) Masson's trichrome staining (3 sections/mouse, Scale bar = 100 µm, *n* = 4 mice). L) Representative immunofluorescence images and quantitation of Z‐disc organization and F‐actin architecture in FBXL4‐cKI mice following TAC‐induced hypertrophy, stained with DAPI (blue), ACTN2 (red), and α‐actinin (green), 3 sections/mouse, *n* = 4 mice. Scale bars: upper, 100 µm; lower, 20 µm. M) Representative TEM analysis of sarcomere structure in FBXL4‐cKI mice following TAC‐induced hypertrophy. The red and yellow arrows indicate Z‐disc and M‐band, respectively. Scale bars, 100 µm and 20 µm. *n* = 3/group. (N) Representative western blot analysis and summary data of ANP and β‐MHC and in cardiac tissues. *n* = 6. (O) qRT‐PCR analyses of ANP, BNP, and β‐MHC mRNA expression in cardiac tissues. *n* = 8. n represents the number of independent samples per group. Data are shown as mean ± SD. ^*^ *p* < 0.05, ^**^ *p* < 0.01, and ^***^ *p* < 0.001. Statistical differences were assessed by obtained one‐way ANOVA followed by Sidak post hoc multiple comparisons test (C, F, H, I, K, N, O).

To elucidate the role of FBXL4 in cardiac hypertrophy in vivo, conditional cardiomyocyte‐specific FBXL4 knock‐in (cKI) mice were generated by crossing FBXL4^fl/fl^ mice with Myh6‐Cre transgenic mice (Figure S5A,B). The successful overexpression of FBXL4 in the heart was confirmed by Western blot and qRT–PCR analyses (Figure S5C,D). Echocardiography revealed that the FBXL4^fl/fl^ mice developed dilated left ventricles and cardiac dysfunction at 8 weeks post TAC, characterized by reduced EF%, FS%, and E/A ratios and increased LVIDd and LVIDs, whereas these deteriorations were significantly attenuated in the TAC mice with cKI (Figure 4A–C). The cardiac morphology and function of cKI mice did not notably differ from those of FBXL4^fl/fl^ mice. However, compared with FBXL4^fl/fl^ control mice, cKI mice displayed reduced cardiac chamber volume (Figure 4D,E) and lower HW/BW and HW/TL ratios (Figure 4F,G). Moreover, TAC‐induced increases in the areas of cell size and fibrosis were abrogated in FBXL4‐cKI mice (Figure 4H–K). IF staining revealed that Z‐disc organization and F‐actin architecture were restored in FBXL4‐cKI mice following TAC‐induced hypertrophy (Figure 4L). Consistent with these findings, compared with control mice, FBXL4‐cKI mice maintained sarcomeric integrity with normalized Z‐line/M‐band alignment after TAC surgery (Figure 4M). Western blot analysis further confirmed the reduction in the protein expression levels of the hypertrophic markers ANP and β‐MHC in the TAC‐treated FBXL4‐cKI mice (Figure 4N), which was accompanied by decreased mRNA expression levels of ANP, BNP, and β‐MHC (Figure 4O). Collectively, these results indicate that the cardiomyocyte‐specific overexpression of FBXL4 attenuates TAC‐induced cardiac dysfunction and sarcomeric disorganization.

### 2.5 FBXL4 Attenuates Ang II‐Induced Cardiomyocyte Hypertrophy In Vitro

Next, we directly investigated the role of FBXL4 in cardiomyocytes by stimulating NMCMs transfected with small interfering RNA targeting FBXL4 (siRNA‐FBXL4) or a scrambled negative control (NC). Si‐FBXL4 effectively decreased the protein expression level of FBXL4 in NMCMs (Figure S6A). IF staining for α‐actinin revealed that FBXL4 knockdown increased the size of cardiomyocytes (Figure S6B). Similarly, hypertrophy markers were dramatically increased at both the protein and mRNA levels after FBXL4 knockdown in NMCMs (Figure S6C–G). Thus, consistent with the in vivo findings, the downregulation of FBXL4 induced cardiomyocyte hypertrophy in vitro.

Conversely, FBXL4 overexpression had the opposite effect on FBXL4 silencing in NMCMs (Figure S6H). FBXL4 overexpression mitigated Ang II‐induced increases in cell size and ANF, BNP, and β‐MHC expression at both the protein and mRNA levels (Figure S6I–N).

### 2.6 FBXL4 Physically Interacts With PFN1 to Promote Its Degradation

**FIGURE 5:**
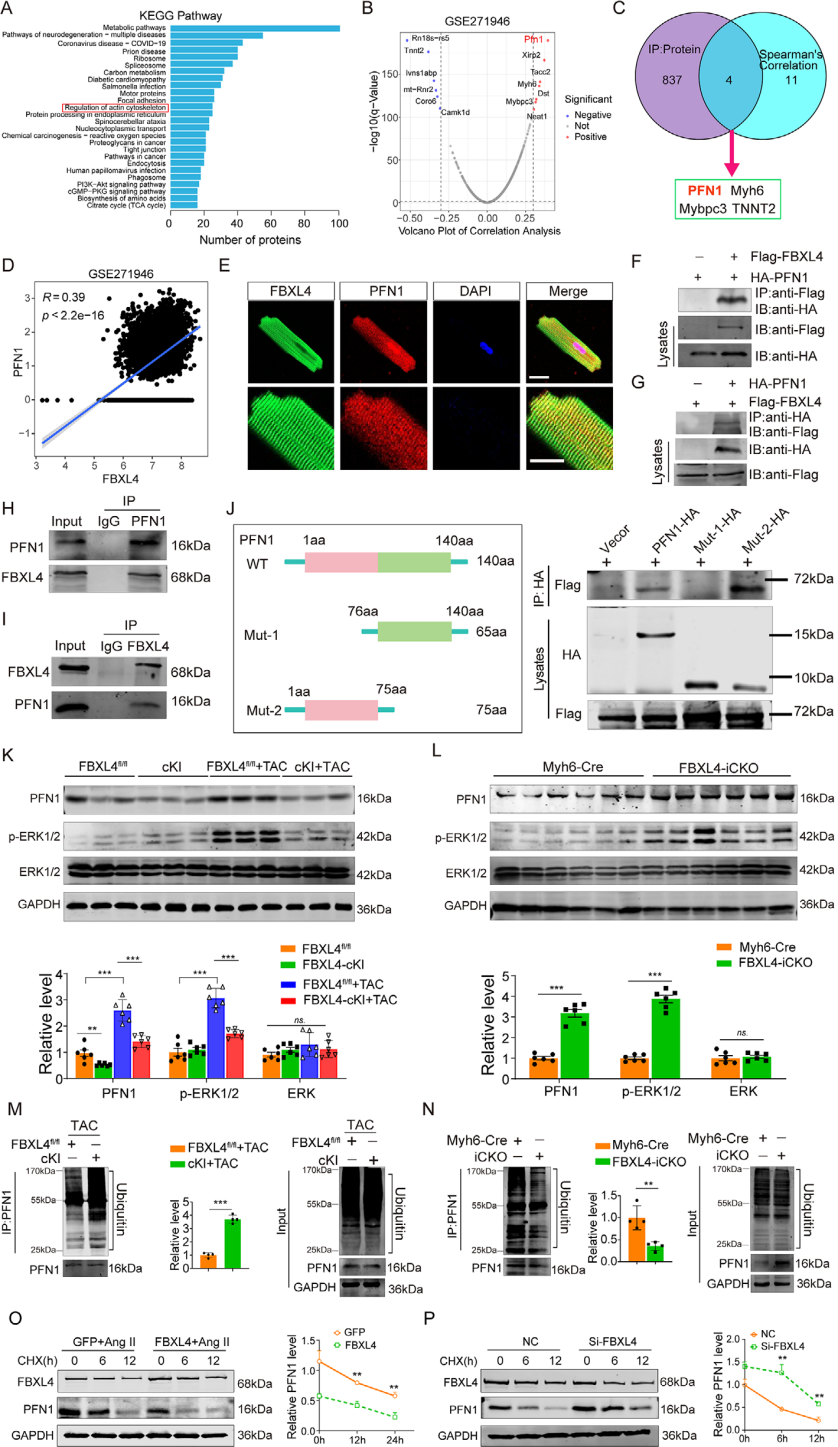
FBXL4 physically interacts with PFN1 to promote its degradation. (A) Bar plot showing Kyoto Encyclopedia of Genes and Genomes (KEGG) analysis of FBXL4‐binding genes. (B) The volcano plot highlights the top 13 genes exhibiting the most significant correlations with FBXL4. (C) The Venn diagram illustrates the overlap between 837 proteins identified by mass spectrometry and candidates prioritized by Spearman's correlation analysis, defining the putative substrate of FBXL4. (D) Scatter plots illustrating the correlation between FBXL4 and PFN1 in the single‐cell dataset (GSE120064): A two‐tailed *p*‐value < 0.05 denotes statistical significance, and an absolute Spearman correlation coefficient |R| ≥ 0.30 indicates a meaningful association. (E) IF staining showing the cellular distribution of FBXL4 and PFN1 in CMs. FBXL4 stained in green, PFN1 stained in red, and the nucleus stained in blue with DAPI. Scale bars: upper, 100 µm; lower, 10 µm. (F, G) Lysates from HEK293T cells transfected with Flag‐FBXL4 and HA‐PFN1 plasmids were immunoprecipitated with anti‐Flag/anti‐HA followed by WB with anti‐Flag (FBXL4) and anti‐HA (PFN1) (*n* = 3). (H, I) Endogenous protein interactions were examined in cardiomyocyte lysates immunoprecipitated (IP) with anti‐rabbit IgG or anti‐FBXL4/PFN1 antibody, and analyzed by Western blot with antibodies to detect PFN1 and FBXL4 (*n* = 3). (J) Schematic diagram for the function domain deletion mutant of PFN1 (left). Western blot of whole cell lysates and anti‐HA Co‐IP samples derived from HEK293T cells transfected with the indicated constructs (right), *n* = 3. (K) Representative immunoblotting analysis and quantification of PFN1, phosphorylated ERK1/2 (p‐ERK1/2 at Thr202/Tyr204), and ERK1/2 protein levels in FBXL4^fl/fl^ and FBXL4‐cKI mice hearts following TAC‐induced hypertrophy (*n* = 6 hearts per group). Normalize phospho‐ERK1/2 to total ERK1/2. Normalize PFN1 and ERK1/2 to β‐actin. β‐actin was used as an internal control. (L) Representative immunoblotting analysis and quantification of PFN1, p‐ERK1/2, and ERK1/2 protein levels in FBXL4‐iCKO and Myh6‐Cre hearts (*n* = 6 hearts per group). Normalize phospho‐ERK1/2 to total ERK1/2. Normalize PFN1 and ERK1/2 to GAPDH. GAPDH was used as an internal control. (M, N) Lysates from heart tissues of FBXL4‐cKI or FBXL4‐iCKO mice were immunoprecipitated with anti‐PFN1 antibody and blotted with total ubiquitin‐conjugated protein or PFN1 body, and quantification of the relative ubiquitinated PFN1 level (*n* = 3). (O, P) NMCMs were infected with Ad‐GFP, Ad‐FBXL4, siRNA‐control or siRNA‐FBXL4, and then treated with Cycloheximide (CHX, 10 µm) for the indicated time periods. Representative western blot analysis of FBXL4 and PFN1 protein levels for each group (left), and quantification of PFN1 level (right; *n* = 4). *n* represents the number of independent samples per group. Data are shown as mean ± SD. *ns*. indicates no significance. ^*^ *p* < 0.05, ^**^ *p* < 0.01, and ^***^ *p* < 0.001. Statistical differences were assessed by one‐way ANOVA followed by Sidak post hoc multiple comparisons test (K) and unpaired t test with Welch's correction (L, M, N). Two‐way ANOVA with Bonferroni's post‐hoc test was used to determine the difference among the treatment groups and different time points (O, P).

To elucidate the molecular mechanism underlying the antihypertrophic effect of FBXL4, we performed coimmunoprecipitation (co‐IP) coupled with mass spectrometry (MS) using lysates from NMCMs. Based on the established colocalization of FBXL4 with cytoskeletal proteins in adult cardiomyocytes, KEGG pathway analysis revealed enrichment of multiple interactors involved in the regulation of the actin cytoskeleton (Figure 5A). To identify genes coexpressed with FBXL4 in heart failure, we integrated single‐cell RNA sequencing data from failing murine hearts (GSE271946) and identified the top 20 genes most strongly correlated with its expression (Figure 5B). This list was intersected with 837 candidate FBXL4‐interacting proteins from mass spectrometry analyses. Among the resulting overlapping genes, we prioritized PFN1 for further investigation, as FBXL4 deficiency did not alter the expression of other candidates, such as MYH6, Mybpc3, or TNNT2 (Figure S7A,B). Given its established role in regulating sarcomeric structure and promoting cardiomyocyte hypertrophy [20], we identified PFN1 as the sole physiological substrate of FBXL4 (Figure 5C). FBXL4 and PFN1 were analyzed by Spearman correlation, with *p* < 0.05 indicating statistical significance and an absolute correlation coefficient (R) greater than 0.3 indicating a significant correlation (Figure 5D). Therefore, we further explored the interaction between FBXL4 and PFN1 in cardiomyocytes. Previous findings confirmed the periodic distribution of PFN1 along myofibrillar in cardiomyocytes [20, 25]. Confocal microscopy further revealed that FBXL4 and PFN1 are predominantly localized in the cytoplasm and exhibit recurring colocalization along the sarcomeric architecture (Figure 5E). HEK‐293T cells were cotransfected with Flag‐FBXL4 and HA‐PFN1. Coimmunoprecipitation followed by immunoblot analysis demonstrated that FBXL4 interacts with PFN1 (Figure 5F,G). Moreover, endogenously expressed PFN1 and FBXL4 could interact with each other in NMCMs (Figure 5H,I). To map the domain for the interaction between FBXL4 and PFN1, we tested the ability of full‐length Flag‐tagged FBXL4 and HA‐tagged PFN1 deletion mutants to bind to full‐length Flag‐tagged FBXL4 expressed in HEK293T cells. Coimmunoprecipitation assays with anti‐Flag antibodies revealed that amino acids 1–75 of PFN1 were required for its binding to FBXL4 (Figure 5J). Previous studies have shown that PFN1 overexpression promotes myocardial hypertrophy by activating the ERK1/2 MAPK signaling pathway and inducing sarcomeric reorganization. We found that cardiac‐specific overexpression of FBXL4 significantly reduced PFN1 and phosphorylated ERK1/2 (p‐ERK1/2 at Thr202/Tyr204) levels, whereas total ERK expression remained unaltered (Figure 5K). Conversely, FBXL4‐deficient murine hearts exhibited elevated PFN1 and p‐ERK1/2 levels without affecting total ERK expression (Figure 5L).

We simultaneously assessed the effects of FBXL4 on the PFN1 signaling pathway in cultured NMCMs. FBXL4 knockdown increased the protein expression levels of PFN1 and p‐ERK1/2 (Figure S8A,B). Conversely, FBXL4 overexpression attenuated the angiotensin II‐induced upregulation of PFN1 and p‐ERK1/2 expression (Figure S8E,F). Morphologically, FBXL4 depletion significantly increased the surface area of cardiomyocytes, accompanied by sarcomere disorganization (Figure S8C and Figure 8D). These structural anomalies were ameliorated by FBXL4 overexpression (Figure S8G,H). Although FBXL4 has been linked to mitochondrial regulation, our data indicate that its cardioprotective function is independent of mitochondrial pathways. Neither knockdown nor overexpression of FBXL4 affected the mitochondrial membrane potential, ROS production, or the activity of respiratory chain complexes (Figure S9A–N). Correspondingly, TEM analysis confirmed that the mitochondrial pathology observed in TAC mice, such as swelling, cristae disorganization, and alterations in mitochondrial number, was not ameliorated in FBXL4‐cKI mice (Figure S9O). Importantly, despite these persistent mitochondrial abnormalities, sarcomere integrity and normal Z‑line/M‑band alignment were preserved in FBXL4‑cKI mice after TAC surgery (Figure S9O). Together, these results indicate that the cardioprotective function of FBXL4 operates independently of canonical mitochondrial pathways.

As an E3 ubiquitin ligase, the FBXL4 gene product belongs to the F‐box protein family (FBLs), which recognizes specific substrates through different protein–protein interaction domains [26]. Notably, FBXL4 depletion or overexpression did not alter PFN1 mRNA levels either in vivo or in vitro (Figure S10). We thus investigated whether FBXL4 regulates PFN1 ubiquitination and degradation in cardiomyocytes. As depicted in Figure 5M, ubiquitination of PFN1 increased, and the protein expression of PFN1 decreased in FBXL4‐iCKO mice. Conversely, FBXL4 silencing reciprocally suppressed the ubiquitination of PFN1 (Figure 5N). Similarly, overexpression of FBXL4 increased, whereas knockdown of FBXL4 significantly decreased, the ubiquitination level of PFN1 in NMCMs (Figure S11). Cycloheximide, an inhibitor of protein synthesis [27], was used to determine the degradation rate of the PFN1 protein. Enforced overexpression of FBXL4 significantly shortened the half‐life of the PFN1 protein (Figure 5O), whereas knockdown of FBXL4 with siRNA markedly prolonged the half‐life of the PFN1 protein (Figure 5P). These results demonstrate that FBXL4 binds PFN1 to regulate the ubiquitination and stability of PFN1.

PFN1 undergoes cyclic phosphorylation and dephosphorylation, and phosphorylation impairs its ability to bind to actin, thereby disrupting actin polymerization [28]. In wild‐type (WT) mice subjected to TAC, phosphorylation ratio of PFN1 at Ser138 and Tyr129 was significantly reduced compared with that in sham‐operated controls (Figure S12A–G). Similarly, compared with the Myh6–Cre controls, the cardiac‐specific knockout of FBXL4 decreased PFN1 phosphorylation ratio at these sites (Figure S12H–N). To investigate whether FBXL4 binds preferentially to the phosphorylated or unphosphorylated forms of PFN1, we generated phosphorylation‐deficient mutants at S138 and Y129 by substituting alanine for the respective residues (S138A and Y129A), and phosphomimetic mutants by substituting glutamic acid for the same residues (S138E and Y129E). Co‐immunoprecipitation assays demonstrated strong binding of FBXL4 to both WT PFN1 and its nonphosphorylatable mutants (S138A and Y129A). In contrast, binding to phosphomimetic PFN1 mutants (S138E and Y129E) was substantially reduced (Figure S12O). Consistent with these findings, FBXL4 increased the ubiquitination of WT PFN1 as well as that of the S138A and Y129A mutants (Figure S12P). Together, these results indicate that FBXL4 promotes the ubiquitin‐mediated degradation of PFN1 independent of its phosphorylation status.

### 2.7 PFN1 is a Critical Inducer of Cardiac Hypertrophy and Heart Failure in Mice

To determine whether PFN1 overexpression is sufficient to drive cardiac hypertrophy and heart failure, we performed gain‐of‐function studies. We used an adeno‐associated virus serotype 9 (AAV9) vector under the cardiomyocyte‐specific troponin T (cTnT) promoter to express PFN1 (AAV9‐PFN1), with an AAV9‐cTnT empty vector as a control. Protein analysis confirmed the cardiac‐specific overexpression of PFN1 in AAV9‐PFN1‐treated mice compared with control mice (Figure S13A,B). Serial echocardiography revealed that AAV9‐PFN1–treated mice developed progressive cardiac dysfunction and severe chamber dilation, as indicated by severely impaired E/A ratios, EF%, and FS%, as well as increased LVIDd and LVIDs (Figure S13C–E). Subsequent morphometric analyses corroborated these findings, confirming significant cardiac enlargement, chamber dilation, and ventricular wall thinning in AAV9‐PFN1‐treated mice (Figure S13F–I). Histological assessment revealed significantly increased cardiomyocyte cross‐sectional areas and elevated interstitial fibrosis deposition in AAV9‐PFN1‐treated hearts (Figure S13J,K). Cardiac‐specific overexpression of PFN1 led to significant sarcomere disarray, as evidenced by IF staining and TEM (Figure S13L,M). This structural deficit was accompanied by the concurrent upregulation of the mRNA and protein expression levels of ANP, BNP, and β‐MHC (Figure S13N,O). Importantly, survival analysis revealed that AAV9‐PFN1‐treated mice manifested mortality onset at 1 week, with 75% lethality observed at 8 weeks (Figure S13P).

To establish a direct functional link between PFN1 and cardiac remodeling, we generated a cardiomyocyte‐specific PFN1 knockdown model by intravenous injection of AAV9 carrying a cTnT promoter‐driven small interfering RNA targeting PFN1 (si‐PFN1) into C57BL/6 mice (Figure S14A,B). Following eight weeks of pressure overload induced by TAC, mice with PFN1 knockdown resulted in significantly reduced cardiac morphometric and ventricular wall thickness parameters, accompanied by a marked improvement in cardiac function (Figure S14C–E). Histological analyses using WGA, H&E, and Masson's staining revealed that TAC induced pronounced cardiomyocyte hypertrophy and interstitial fibrosis, both of which were substantially attenuated by PFN1 knockdown (Figure S14F–M). Moreover, IF staining and TEM revealed that sarcomeric integrity was significantly better preserved in PFN1‐knockdown hearts than in TAC control hearts, which was accompanied by reduced mRNA expression levels of ANP and β‐MHC (Figure S14N–Q). Collectively, these data identify PFN1 as a critical driver of maladaptive remodeling in response to pressure overload and demonstrate that its cardiomyocyte‐restricted silencing is sufficient to protect sarcomere structure and halt the progression to heart failure.

### 2.8 K70 of PFN1 Is Important for Its K48‐Linked Ubiquitination Mediated by FBXL4

**FIGURE 6:**
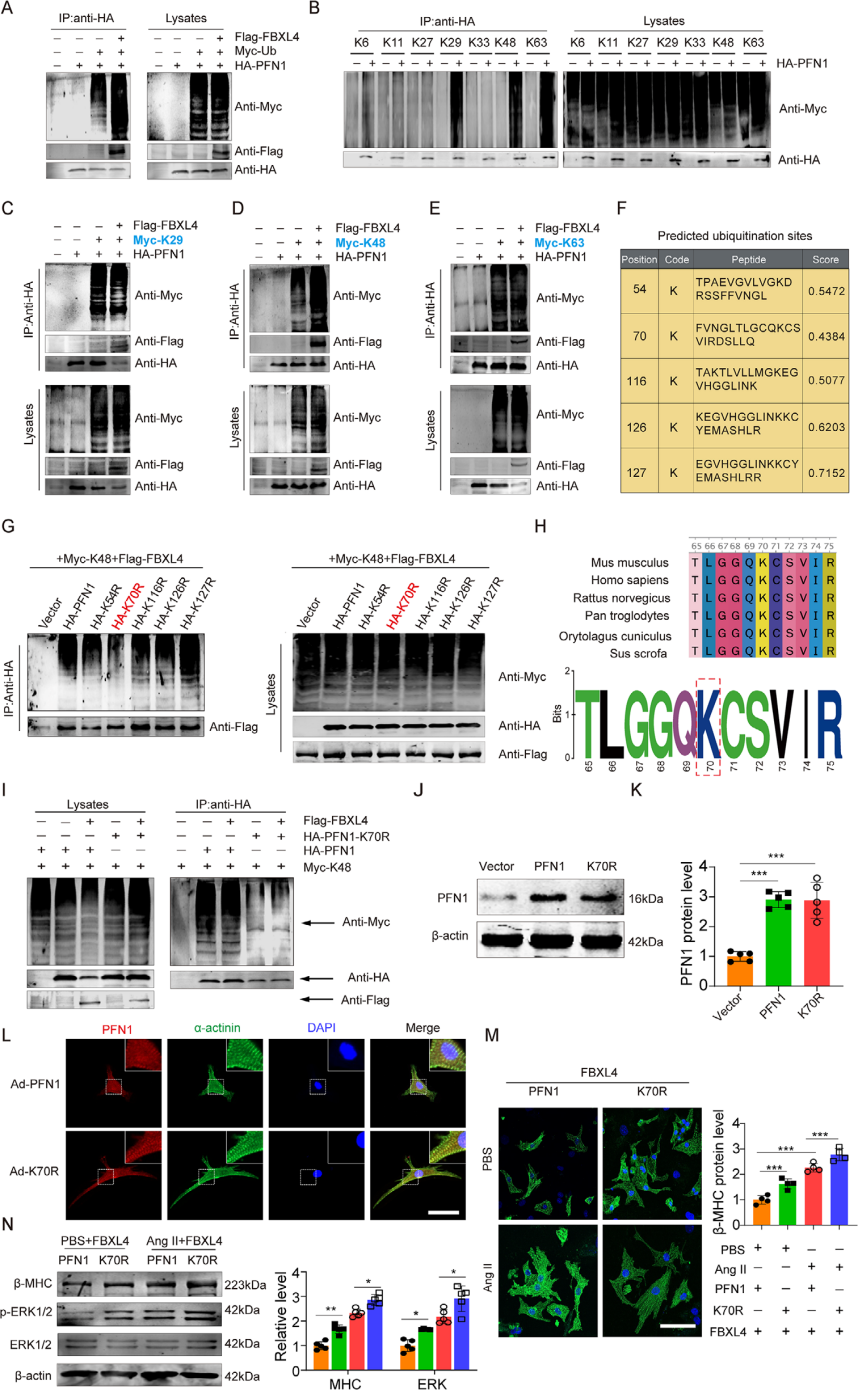
K70 of PFN1 is important for its K48‐linked ubiquitination mediated by FBXL4. (A) HEK293T cells overexpressing FBXL4 were transfected with the indicated plasmid combinations to measure ubiquitination of PFN1. (B) HEK293T cells overexpressing FBXL4 were transfected with the indicated plasmid combinations to measure K6/K11/K27/K29/K33/K48/K63‐linked ubiquitination of PFN1. (C–E) HEK293T cells overexpressing FBXL4 were transfected with the indicated plasmid combinations to measure K29, K48, and K63‐linked ubiquitination of PFN1. (F) The ubiquitination sites of PFN1 were predicted by BDM‐PUB and UBPRED. (G) HEK293T cells were transfected with the indicated plasmid combinations to measure K48‐linked ubiquitination of PFN1. (H) WebLogo output for amino acid sequences of PFN1 from Mouse, Homo, Rat, Cuniculus, Chimpanzee, and Pig. (I) HEK293T cells overexpressing Flag‐FBXL4 and Myc‐K48 were transfected with the indicated plasmid combinations to measure the ubiquitination of HA‐PFN1/PFN1‐K70R. (J, K) Representative immunoblot and quantification showing PFN1 protein levels in NMCMs following transduction with K70R or WT PFN1, *n* = 5. (L) IF staining showing the cellular distribution of PFN1 and PFN1‐K70R in NMCMs. α‐actinin stained in green, PFN1 stained in red, the nucleus stained in blue with DAPI. Scale bars: 50 µm. (M) Cell size in NMCMs transfected with FBXL4, PFN1, and PFN1‐K70R stimulated with PBS or Ang II for 48 h. Scale bars: 50 µm; (N) Protein levels of β‐MHC, p‐ERK1/2, and ERK in NMCMs transfected with Vector, PFN1, and PFN1‐K70R, *n* = 4. *n* represents the number of independent samples per group. Data are shown as mean ± SD. *ns*. indicates no significance. ^*^ *p* < 0.05, ^**^ *p* < 0.01, and ^***^ *p* < 0.001. Statistical differences were assessed by two‐way ANOVA followed by Sidak post hoc multiple comparisons test (J), one‐way ANOVA with Tukey's multiple comparisons test (N, M).

To explore whether FBXL4 regulates the ubiquitination of PFN1, HEK293T cells were transfected with plasmids encoding HA‐PFN1 and Myc‐Ub, along with Flag‐tagged FBXL4. The results clearly demonstrated that the ubiquitination of PFN1 was enhanced by FBXL4 overexpression (Figure 6A). There are seven lysine residues within ubiquitin, allowing site‐specific ubiquitination to occur at K6 (Lysine, abbreviated K), K11, K27, K29, K33, K48, or K63 [29]. To investigate the type of ubiquitin chain linkage in PFN1, we cotransfected seven types of ubiquitin plasmids (each containing only one wild‐type lysine site with the others mutated) with an empty vector or HA‐PFN1 for ubiquitination detection. The results revealed that the ubiquitination signal of Flag‐PFN1 was significantly enhanced after cotransfection with K29, K48, and K63 but not with K6, K11, K27 or K33 (Figure 6B), suggesting that PFN1 can generally be ubiquitinated by K29, K48, or K63. To determine the specific site that is mediated by FBXL4 for PFN1 ubiquitination, we used HA‐tagged K29, K48, and K63 ubiquitin to perform ubiquitination assays. The results indicated that overexpression of FBXL4 clearly increased K48‐linked but not K29‐ or K63‐linked ubiquitination of PFN1 (Figure 6C–E).

We then employed bioinformatics methods to predict the possible ubiquitination sites of PFN1. Based on the intersection predicted by BDM‐PUB (http://bdmpub.biocuckoo.org/results.php) and UBPRED (http://www.ubpred.org/), eight lysine residues (K54, K70, K116, K126, K127) were selected to construct PFN1 mutants (K to R, arginine abbreviation R) (Figure 6F). HEK293T cells were transfected with plasmids encoding wild‐type (WT) and PFN1 mutants (K to R), together with Myc‐K48 and Flag‐FBXL4. Subsequent ubiquitination assays revealed that FBXL4‐mediated K48‐linked ubiquitination of PFN1 was markedly reduced after mutation at K70 of PFN1 (Figure 6G). The PROSITE sequence logo for PFN1 indicated that the K70 site is highly conserved (Figure 6H). We additionally explored the effect of PFN1‐K70 site mutation on K48‐linked ubiquitination. Mutation of the K70 site significantly blocked the K48‐linked ubiquitination of PFN1 mediated by FBXL4 (Figure 6I). These findings suggest that K70 of PFN1 is important for its K48‐linked ubiquitination mediated by FBXL4. The impact of the K70R mutation on the hypertrophic phenotype of cardiomyocytes was then investigated. Successful transduction of K70R and WT PFN1 into NMCMs was confirmed by substantial increases in the corresponding protein levels posttransfection (Figure 6J,K). Notably, despite colocalizing with α‐actinin, similar to wild‐type PFN1, the PFN1‐K70R mutation specifically reversed the inhibitory effects of FBXL4 overexpression on Ang II‐induced responses, including increases in p‐ERK1/2 levels, β‐MHC expression, and cell size (Figure 6L–N). Together, these results indicate that lysine 70 of PFN1 is a critical site for its K48‐linked ubiquitination by FBXL4 and that impairment of this modification promotes cardiomyocyte hypertrophy.

### 2.9 AAV9‐Mediated Knockdown or Pharmacological Inhibition of PFN1 Partially Reversed Cardiac Dysfunction and HF in FBXL4‐iCKO Mice

**FIGURE 7:**
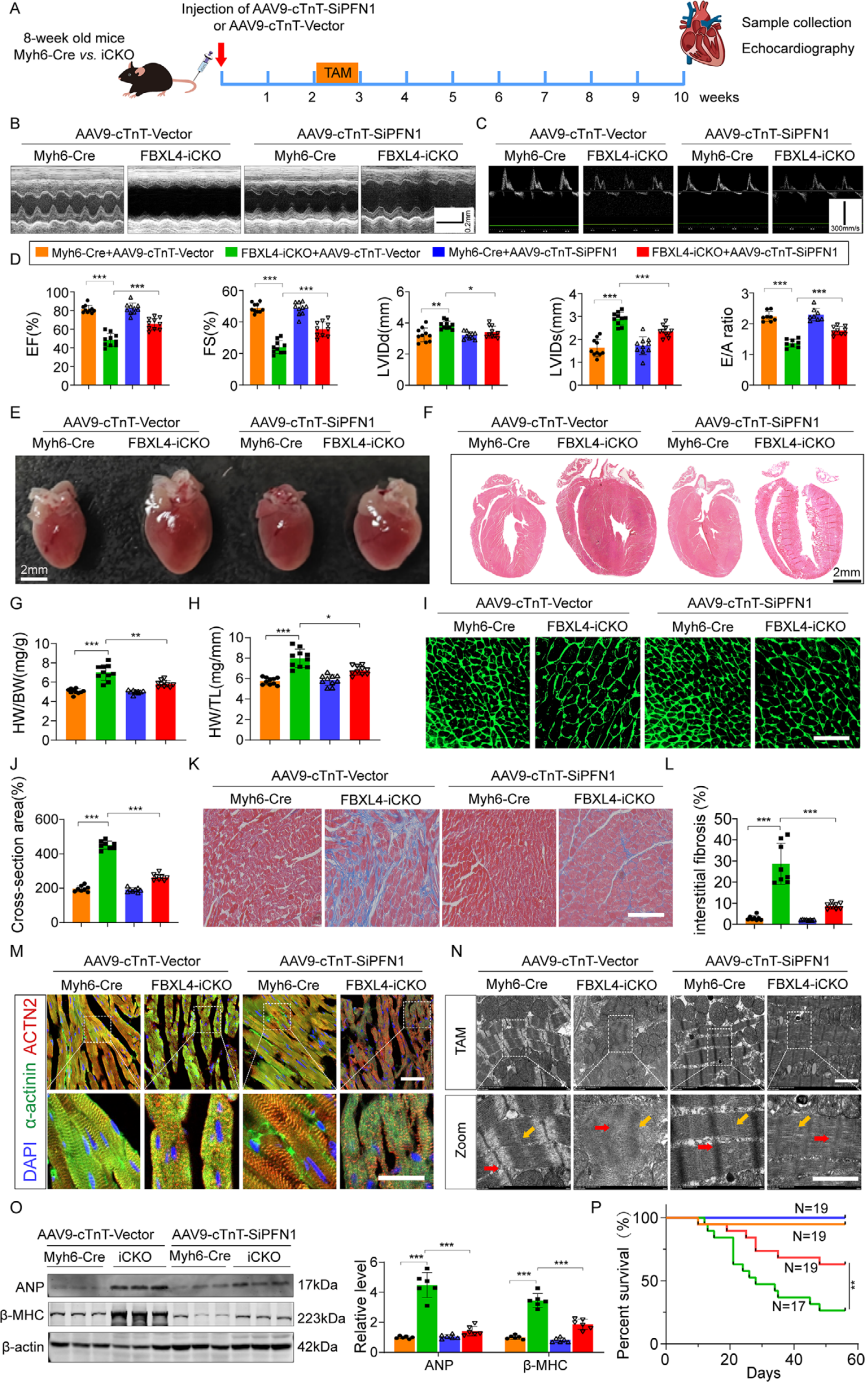
Knockdown of PFN1 partially rescues the HF in FBXL4‐iCKO Mice. (A) Schematic diagram of tail intravenous AAV9‐cTnT‐SiPFN1/Vector and tamoxifen intraperitoneal injection. (B–D) Representative M‐mode echocardiography of the left ventricle and statistical data of EF%, FS%, LVIDd, LVIDs, and E/A ratio. *n* = 8–10/group. (E, F) Gross morphology and HE staining of the hearts (scale bar = 2 mm). (G, H) Heart weight (HW)/body weight (BW) and HW/tibia length (TL) ratios in the different groups, *n* = 10/group. (I, J) Representative images and statistics of WGA staining (2–3 sections/mouse, Scale bar = 100 µm, *n* = 4). (K, L) Masson's trichrome staining (3 sections/mouse, Scale bar = 100 µm, *n* = 4 mice). (M) Representative IF images of Z‐disc organization and F‐actin architecture in AAV9‐cTnT‐Vector or AAV9‐cTnT‐SiPFN1 treated with Myh6‐Cre and FBXL4‐iCKO mice, stained with DAPI (blue), ACTN2 (red), and α‐actinin (green). Scale bars: upper, 100 µm; lower, 20 µm, 3 sections/mouse, *n* = 4 mice. (N) Representative TEM image of sarcomere structure in AAV9‐cTnT‐Vector or AAV9‐cTnT‐SiPFN1 treated with Myh6‐Cre and FBXL4‐iCKO mice. The red and yellow arrows indicate Z‐disc and M‐band, respectively. Scale bars: upper, 100 µm; lower, 20 µm. *n* = 3. (O) Representative western blot analysis and summary data of ANP and β‐MHC and in cardiac tissues, *n* = 6. (P) Survival curve of individual experimental groups. (Myh6‐Cre+AAV9‐ cTnT‐Vector, *n* = 19; FBXL4‐iCKO+AAV9‐cTnT‐Vector, *n* = 19; Myh6‐Cre+AAV9‐cTnT‐SiPFN1, *n* = 19; FBXL4‐iCKO+AAV9‐cTnT‐SiPFN1, *n* = 17). n represents the number of independent samples per group. Data are shown as mean ± SD. ^*^ *p* < 0.05, ^**^ *p* < 0.01, and ^***^ *p *< 0.001. Statistical differences were assessed by two‐way ANOVA followed by Sidak post hoc multiple comparisons test (D, G H, J, L, O), Kaplan–Meier analysis with the log‐rank Mantel‐Cox test (P).

If an increase in PFN1 protein expression indeed mediated the prohypertrophic phenotypes observed in FBXL4‐iCKO mice, then silencing PFN1 expression should eliminate the detrimental changes in the hearts of FBXL4‐iCKO mice. To test this hypothesis, we injected an AAV9 viral vector carrying an siRNA (AAV9‐siPFN1) into FBXL4^fl/fl^ and FBXL4‐iCKO mice to silence the expression of PFN1. The knockdown of PNF1 by AAV9‐siPFN1 was first confirmed by the significant reduction in PFN1 levels in the heart (Figure S15A,B). Echocardiography revealed that AAV9‐siPFN1 attenuated cardiac hypertrophy and HF in iCKO mice, as reflected by improvements in cardiac function, including increases in the E/A ratio and EF% and FS% and decreases in the LVIDd and LVIDs (Figure 7A–D). Deficiency of FBXL4 induced increases in the HW/BW ratio. HW/TL, gross heart size, and mean cross‐sectional area were effectively mitigated by AAV9‐SiPFN1 (Figure 7E–H), as was myocardial interstitial fibrosis, as determined by Masson's staining (Figure 7I–L). Furthermore, IF and TEM revealed preserved sarcomeric ultrastructures in AAV9‐siPFN1‐treated FBXL4‐iCKO mice (Figure 7M,N). The improvements in cardiac structure and function resulting from AAV9‐siPFN1 administration were accompanied by pronounced decreases in ANP and β‐MHC protein expression levels (Figure 7O). Surprisingly, AAV9‐cTnT‐siPFN1 administration significantly increased the survival rate of FBXL4‐iCKO mice (Figure 7P).

To determine whether specific inhibition of PFN1 could rescue cardiac dysfunction in FBXL4‐iCKO mice, we administered PFN1‐IN‐1 (10 mg/kg daily) or vehicle via intraperitoneal injection for seven weeks (Figure S16A). Echocardiographic analysis revealed that PFN1‐IN‐1 treatment alleviated cardiac hypertrophy and improved cardiac function, as indicated by the EF%, FS%, and E/A ratios, along with reduced LVIDd and LVIDs, and improved the myocardial performance index. In contrast, vehicle‐treated mice exhibited no functional improvement (Figure S16B–D). Consistent with the attenuation of cardiac hypertrophy, PFN1‐IN‐1 treatment also reduced heart size and decreased the HW/BW and HW/TL ratios in TAC‐induced mice (Figure S16E–H). Furthermore, PFN1‐IN‐1 treatment attenuated the increase in the cross‐sectional area and the interstitial fibrosis of cardiomyocytes in FBXL4‐iCKO mice (Figure S16I–L). IF staining and TEM confirmed that PFN1 inhibition restored sarcomere structural defects in FBXL4‐deficient cardiomyocytes (Figure S16M,N). Accordingly, the elevated expression levels of the hypertrophy markers ANP, BNP, and β‐MHC in iCKO mice were significantly suppressed by PFN1‐IN‐1 (Figure S16O). Importantly, inhibition of PFN1 markedly improved survival in FBXL4‐iCKO mice (Figure S16P).

Similar qualitative results were observed in complementary in vitro experiments. siRNA‐mediated silencing of FBXL4 markedly exacerbated the hypertrophic phenotype of cardiomyocytes, an effect that was largely reversed by concomitant knockdown of PFN1 via siRNA (si‐PFN1‐2/3) (Figure S17A–F). Similarly, cotransfection with PFN1 abolished the enhanced antihypertrophic effects induced by FBXL4 overexpression (Figure S17G–L). Collectively, these findings demonstrate that FBXL4 modulates cardiomyocyte hypertrophy through a PFN1‐dependent mechanism.

### 2.10 SP1 Transcriptionally Represses FBXL4 Expression During Cardiac Hypertrophy

Given the observed reduction in FBXL4 mRNA expression in cardiac hypertrophy, we next investigated its transcriptional regulation. Using six transcription factor databases (hTFtarget, KnockTF, ENCODE, CHEA, TRRUST, GTRD, and JASPAR), we identified FOXA1, FOXA2, and SP1 as candidate transcription factors with high binding scores to the FBXL4 promoter (Figure S18A). Neither FOXA1 nor FOXA2 knockdown affected FBXL4 expression (Figure S18B–E). In contrast, SP1 depletion increased FBXL4 mRNA and protein levels (Figure S18F–H). Accordingly, treatment of wild‐type mice with the SP1 antagonist mithramycin A (MTM, 150 µg/kg) significantly enhanced FBXL4 expression (Figure S18I,J). Moreover, SP1 protein was also upregulated after TAC surgery (Figure S18K). SP1 is known to modulate gene transcription by binding to GC‐rich promoter elements and has been implicated in regulating cardiac hypertrophic genes [30, 31]. These findings suggest that SP1 may contribute to cardiac hypertrophy by repressing FBXL4 transcription.

JASPAR database analysis predicted multiple GC‐rich SP1 binding sites in the FBXL4 promoter (Figure S18L). Chromatin immunoprecipitation followed by qPCR confirmed the significant enrichment of SP1 at the endogenous FBXL4 promoter (Figure S18M). To assess transcriptional regulation, we cotransfected neonatal mouse cardiomyocytes with SP1‐targeting siRNA and FBXL4 promoter–luciferase constructs. SP1 knockdown enhanced FBXL4 promoter activity, most strongly with a construct containing the −1332 site (FBXL4‐4) (Figure S18N). Furthermore, in cardiomyocytes overexpressing SP1(Figure S18O), subsequent FBXL4 transfection reversed the SP1‐induced exacerbation of Ang II–induced hypertrophy, as shown by reduced cell size, lower β‐MHC protein levels, and decreased ANF, BNP, and β‐MHC mRNA expression levels (Figure S18P–S). Collectively, these results demonstrate that SP1 binds to the FBXL4 promoter and represses its transcription during cardiac hypertrophy.

### 2.11 FBXL4 Deficiency Induces Hypertrophic Phenotypes in hiPSC‐CMs

**FIGURE 8:**
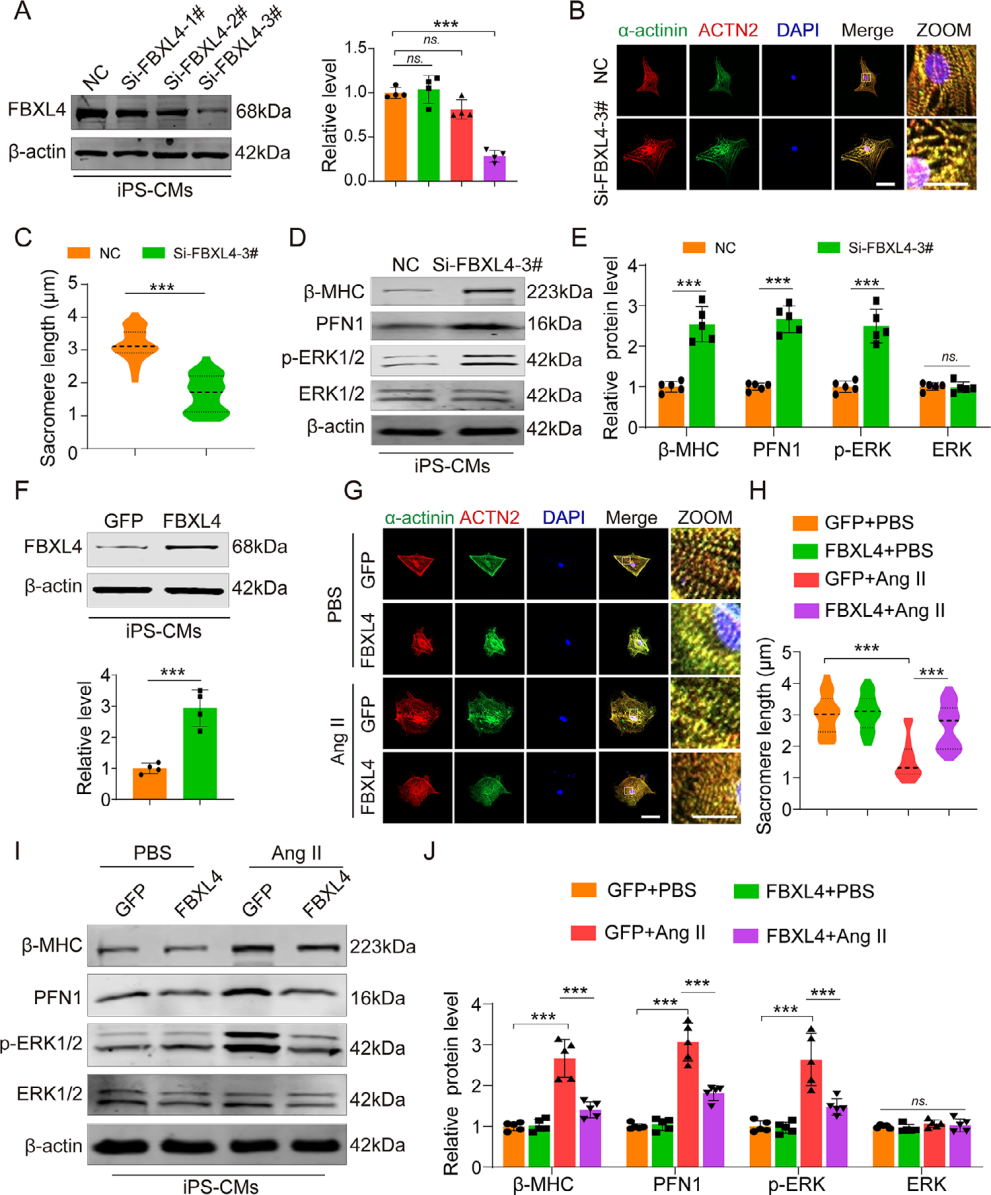
FBXL4 deficiency induces hypertrophic phenotypes in human induced pluripotent stem cell‐derived cardiomyocytes (iPSC‐CMs). (A) Verification of the efficacy of human FBXL4 siRNA (siFBXL4) in silencing FBXL4 expression at the protein levels (*n* = 4) in hiPSC‐CMs. NC is a negative control siRNA. (B, C) Representative images and quantification of myofibrillar disarray in hiPSC‐CMs by staining with ACTN2 (red), α‐actinin (green), and DAPI (blue). Scale bar, 50 µm. Zoomed images show a higher magnification. Scale bar, 1 µm. At least 100 cells were counted in each group from 6 independent experiments. (D, E) Representative immunoblotting analysis and quantification of β‐MHC, PFN1, pERK1/2, and ERK1/2 protein levels after transfection of siFBXL4 in hiPSC‐CMs. (*n* = 4). (F) Verification of the efficacy of the human FBXL4 plasmid in FBXL4 overexpression at the protein levels (*n* = 4). GFP is a negative control FBXL4. (G, H) Representative images and quantification of myofibrillar disarray in hiPSC‐CMs by staining with ACTN2 (red), α‐actinin (green), and DAPI (blue). Scale bar, 50 µm. Zoomed images show a higher magnification. Scale bar, 1 µm. At least 100 cells were counted in each group from 6 independent experiments. (I, J) Representative immunoblotting analysis and quantification of β‐MHC, PFN1, pERK1/2, and ERK1/2 protein levels under Ang II‐induced hiPSC‐CMs hypertrophy in the FBXL4 and GFP group (*n* = 4). n represents the number of independent samples per group. Data are shown as mean ± SD. *n.s* indicates no significance. ^*^ *p* < 0.05, ^**^ *p* < 0.01, and ^***^ *p *< 0.001. Statistical differences were assessed by one‐way ANOVA followed by the Dunn post hoc multiple comparisons test (A), two‐tailed unpaired Student's *t* tests (C, E, F), two‐way ANOVA followed by Sidak post hoc multiple comparisons test (H, J).

To investigate the conserved role of FBXL4 in cardiac hypertrophy across species, we transfected human induced pluripotent stem cell‐derived cardiomyocytes (hiPSC‐CMs) with FBXL4‐targeting siRNA to knock down its expression (Figure 8A). Silencing FBXL4 led to increased cell size and sarcomere disorganization in hiPSC‐CMs (Figure 8B,C). Conversely, overexpression of human FBXL4 via a plasmid vector attenuated Ang II‐induced hypertrophy and preserved sarcomere structure (Figure 8F–H). We further evaluated the influence of FBXL4 on the PFN1 signaling pathway. Western blot analysis revealed that FBXL4 knockdown upregulated the expression of β‐MHC and PFN1 and phosphorylated ERK1/2 (Figure 8D,E), whereas FBXL4 overexpression reduced their expression levels following Ang II stimulation (Figure 8I,J). Together, these results identify FBXL4 as a potential therapeutic target for treating cardiac hypertrophy in humans.

## 3 Discussion

In this study, we found that FBXL4 expression was significantly downregulated in cardiomyocytes during pathological cardiac hypertrophy. We identified FBXL4 as a novel antihypertrophic factor that preserves cardiac function during cardiac hypertrophy and heart failure. Mechanistically, we demonstrated that FBXL4 mediates the K48‐linked ubiquitination of PFN1 at lysine 70 (K70), thereby suppressing ERK1/2 signaling and preserving sarcomeric integrity. Furthermore, the transcription factor SP1 was found to repress FBXL4 transcription in the context of myocardial hypertrophy. These findings establish a mechanistic link between FBXL4/PFN1‐mediated myofilament remodeling and pathological cardiac hypertrophy and suggest that FBXL4 may serve as a promising therapeutic target for the treatment of hypertrophic cardiomyopathy (Graphic Abstract).

The ubiquitin–proteasome system (UPS) facilitates selective protein degradation via a hierarchical E1–E2–E3 enzyme cascade, culminating in the polyubiquitylation of substrates and their subsequent delivery to the 26S proteasome for ATP‐dependent proteolysis [32, 33, 34]. By tightly controlling proteostasis, the UPS orchestrates essential cellular functions, such as cell cycle progression, signal transduction, and responses to cellular stress [35, 36, 37], and therefore, its dysfunction has been implicated in diverse pathologies, including neurodegeneration and cancer [38, 39]. Notably, FBXL4, an F‐box protein that serves as a substrate recognition component of the SCF ubiquitin ligase complex, plays a critical role in preserving mitochondrial DNA function [10, 40]. A previous study demonstrated that FBXL4 confers protection against HFpEF by modulating Drp1‐dependent mitochondrial dynamics and downstream SERCA2a activity [13]. Conversely, FBXL4 overexpression promotes cell death while suppressing autophagy in H9c2 cardiomyocytes exposed to chronic intermittent hypoxia (CIH) [41]. The opposing phenotypes observed in the two studies likely stem from fundamentally distinct pathophysiological mechanisms: the central pathophysiological mechanism underlying CIH‐induced cardiac injury encompasses oxidative stress exacerbation, sympathetic overactivation, systemic inflammatory cascades, and endothelial dysfunction, all of which are directly induced by hypoxia–reoxygenation cycles [42, 43, 44]. Conversely, the core pathological manifestation of HFpEF is left ventricular diastolic dysfunction, chiefly attributable to myocardial hypertrophy, interstitial fibrosis, microvascular impairment, and chronic systemic inflammation [44, 45]. These observations reveal that FBXL4 mediates spatiotemporally divergent effects throughout the progression of cardiac injury and clinically distinct cardiac disease entities, a compelling premise that requires mechanistic investigation. To address this hypothesis, we engineered inducible, cardiomyocyte‐specific FBXL4 knockout (iCKO) mice by crossing FBXL4^fl/fl^ mice with Myh6‐MerCreMer transgenic mice, given the hereditary disease phenotypes associated with FBXL4 deficiency. Notably, we observed significant downregulation of FBXL4 expression in heart tissue from patients with heart failure, hypertrophic mouse hearts, and Ang II‐treated cardiomyocytes. Conditional ablation of FBXL4 in cardiomyocytes precipitated a robust hypertrophic response in vivo, establishing its function as a novel pathophysiological modulator of cardiac remodeling and failure progression.

To identify downstream targets of FBXL4, we conducted liquid chromatography–tandem mass spectrometry (LC–MS) on angiotensin II (Ang II)‐induced hypertrophic cardiomyocytes and identified multiple putative FBXL4 substrates. Given the striking myofilament disarray in FBXL4‐iCKO mice, KEGG pathway analysis revealed significant enrichment of cytoskeleton‐associated proteins. Integration of single‐cell RNA sequencing data from human failing hearts enabled Spearman correlation analysis between FBXL4 and cardiomyocyte‐enriched transcripts, identifying profilin 1 (PFN1) as a putative FBXL4‐interacting partner. PFN1 is a ubiquitously expressed actin‐binding protein involved in a broad spectrum of cellular processes, including actin polymerization and transcriptional regulation [46, 47, 48]. Consequently, PFN1 is essential for diverse cellular functions, including membrane trafficking, endocytosis, cell cycle progression, migration, proliferation, survival, transcription, stemness, and autophagy [49, 50, 51]. Our findings demonstrate that PFN1 acts as a prohypertrophic factor contributing to pathological cardiac remodeling. Moreover, PFN1 silencing reversed the detrimental effects of FBXL4 deletion on cardiac function, highlighting the pivotal role of PFN1 in mediating the function of FBXL4. Although FBXL4 is known primarily for its role in mitochondrial regulation, our findings demonstrate that its cardioprotective function is mitochondria independent. Instead, FBXL4 acts in a context‐dependent manner, likely by engaging specific substrates across different cellular environments and stress conditions. Using subcellular localization analysis and mass spectrometry‐based proteomic screening in cardiomyocytes, we provide the first evidence that FBXL4 maintains sarcomere integrity and cardiac function by targeting PFN1, revealing a functional FBXL4–PFN1 interaction in the mammalian myocardium.

Additionally, we identified PFN1 as a novel substrate of the E3 ubiquitin ligase FBXL4. Coimmunoprecipitation assays defined the FBXL4–PFN1 interaction motif, revealing that FBXL4 directly binds the N‐terminal region (residues 1–75) of PFN1 and promotes its proteasomal degradation through K48‐linked polyubiquitination. Within this region, we identified lysine 70 (K70) as the critical residue for FBXL4‐mediated ubiquitination. FBXL4 preferentially interacts with unphosphorylated PFN1 and drives its ubiquitination and degradation independent of its phosphorylation status. The functional significance of this modification was confirmed by the PFN1‐K70R mutation, which abolished the cardioprotective effects of FBXL4 and underscored the essential role of K70 ubiquitination in suppressing cardiac hypertrophy and heart failure. PFN1 is known to be regulated by posttranslational modifications, such as phosphorylation at S137 in breast cancer cells and Y129 during VEGF‐induced angiogenesis [52]; However, systematic mapping of its ubiquitination sites remains lacking. Our work thus provides the first evidence identifying K70 as a functional ubiquitination site that governs PFN1 stability in the context of cardiac pathology.

Our findings revealed that the expression of SP1, a transcriptional coactivator, was significantly upregulated under hypertrophic stress. Knockdown or pharmacological inhibition of PFN1 increased FBXL4 expression. Furthermore, we demonstrated for the first time that SP1 binds to the FBXL4 promoter region and transcriptionally represses its expression, supporting a key role for the SP1–FBXL4 axis in cardiac hypertrophy. Although SP1‐mediated repression of SFRP5 has been linked to atrial fibrillation, our results reveal a distinct pathway through which SP1 promotes hypertrophy. In cardiomyocytes, SP1 competes with SP3 to activate the cTnT promoter, triggering a cAMP‐dependent signaling cascade that increases calcium influx and elevates cytosolic calcium levels [53]. SP1 upregulation is also linked to a network of hypertrophy‐related factors, including KLF5, lncRNAs (CTBP1‐AS, SYHE1‐AS1, and SNHG14), and the promoters of PCDH17 and PDE5 [30]. Collectively, our findings highlight SP1 as a potential therapeutic target for treating pathological cardiac hypertrophy and heart failure.

This study has several limitations. First, as the PFN1 pathway represents just one of many signaling cascades involved in cardiac hypertrophy, we cannot exclude the possibility that FBXL4 regulates cardiac remodeling and heart failure through PFN1‐independent mechanisms. Second, while we have shown that PFN1 knockout or pharmacological inhibition partially rescues cardiac dysfunction in FBXL4‐iCKO mice, whether FBXL4 exerts its cardioprotective effects exclusively through PFN1 remains unresolved. To address this, future studies should employ cardiomyocyte‐specific FBXL4‐PFN1 double‐knockout and double‐overexpression models to delineate the full scope of the FBXL4‐dependent regulatory network. Third, although we identified lysine 70 (K70) of PFN1 as a ubiquitination site, its functional relevance requires further characterization. Generating myocardial cell‐specific K70R point mutant mice via CRISPR/Cas9‐mediated editing will be an essential step toward assessing the structural and functional impact of this mutation on sarcomeres under both physiological and stress conditions. Finally, high‐throughput screening approaches may facilitate the discovery of small‐molecule modulators of the SP1‐FBXL4‐PFN1 signaling axis, potentially offering novel therapeutic strategies for heart failure.

In summary, we demonstrate a novel role for FBXL4 in cardiac hypertrophy. FBXL4 protects against pathological cardiac hypertrophy by alleviating PFN1 expression. These findings indicate that FBXL4 may be a potential target for therapeutic interventions in pressure‐induced cardiac hypertrophy and heart failure.

## 4 Experimental Section

More detailed methods are described in the Supplementary Materials.

### 4.1 Human Cardiac Samples

Healthy human cardiac samples were collected from the tissue bank of the Heilongjiang Academy of Medical Sciences (Harbin, China), and diseased samples from patients with heart failure (HF). Detailed information on the donors and patients was described previously [12]. Demographic characteristics of the human subjects from whom the heart tissues were used are summarized in Supplementary Table S1. The use of human cardiac tissues for the present study was approved by the Ethics Committee of Harbin Medical University (YJSKY2025‐051). Our study protocols complied with the guidelines that govern the use of human tissues outlined in the Declaration of Helsinki.

### 4.2 Experimental Animals

All experiments involving animals were approved by the Ethic Committees of Harbin Medical University and conformed to the Guide for the Care and Use of Laboratory Animals published by the US National Institutes of Health (NIH Publication No. 85–23, revised 1996). Mice of experimental were randomly assigned to experimental groups using computer‐generated randomization. All experiments were permitted by the Animal Care and Use Committee of Harbin Medical University (SYDW2025‐115).

### 4.3 Mouse Model of Cardiac Hypertrophy Induced by Aortic Constriction and Pharmacological Intervention

Transverse aortic constriction (TAC) was performed as previously described [54]. Mice were randomly assigned to sham or TAC groups and anesthetized with intraperitoneal avertin (0.2 g/kg). Following orotracheal intubation with a 20‐gauge catheter, mice were mechanically ventilated at 100 breaths/min with a tidal volume of 0.3 mL. The transverse aorta was ligated using a 7‐0 silk suture tied against a 27‐gauge needle, which was then removed to create a constriction of 0.4 mm in diameter. Sham‐operated mice underwent the same procedure without aortic ligation. Upon study completion, mice were euthanized by cervical dislocation under deep anesthesia induced with 3% pentobarbital sodium or 2.5% isoflurane. Furthermore, mithramycin A (MCE. HY‐A0122) and PFN1‐IN‐1 (MCE. HY‐136808) were administered via intraperitoneal injection.

### 4.4 Echocardiography

Left ventricular (LV) function was assessed by echocardiography using a Vevo2100 system (VisualSonics, Toronto, Canada) with a 10 MHz transducer. M‐mode recordings were obtained as described previously [54, 55] to measure LV internal dimensions at end‐diastole (LVIDd) and end‐systole (LVIDs). From these, ejection fraction (EF) and fractional shortening (FS) were calculated, with FS derived as ((LVIDd—LVIDs) / LVIDd) × 100. Diastolic function was evaluated from apical 4‐chamber views using pulsed‐wave and tissue Doppler imaging at the mitral valve. The E/A ratio, representing early (E) to atrial (A) filling velocities, was measured from pulsed‐wave Doppler traces.

### 4.5 Co‐Immunoprecipitation (Co‐IP)

co‐IP assays were performed following established protocols [54]. Transfected neonatal mouse cardiomyocytes (NMCMs), heart tissues, or co‐transfected HEK293T cells were lysed in ice‐cold IP buffer (20 mm Tris pH 7.5, 150 mm NaCl, 1% Triton X‐100, 0.5% sodium pyrophosphate, 0.1 mm β‐glycerophosphate, EDTA, 0.1 mm Na3VO4, 1 µg/mL leupeptin) supplemented with 1% phosphatase inhibitor cocktail. Following lysis on ice for 10 min and centrifugation (12,000 × g, 15 min, 4°C), the supernatants were incubated with specific antibodies and Protein A/G Magnetic Beads overnight at 4°C. The bead complexes were then washed three times with cold IP buffer, resuspended in 1× SDS loading buffer, and boiled at 95°C for 10 min for subsequent western blot analysis.

### 4.6 Ubiquitination Assays

Following 6 h MG132 treatment (50 µm), cells were harvested. Cultured NMCMs, HEK293T cells, or cardiac tissues underwent ice‐cold lysis for 30 min in buffer (50 mm Tris pH 7.4, 150 mm NaCl, 1% Triton X‐100, 1% sodium deoxycholate, 1% SDS) supplemented with phosphatase inhibitors (0.1 mm Na_3_VO_4_, 10 mm NaF, 1.5 mm EDTA) and protease inhibitors (1 µg/mL leupeptin, 1% cocktail; Beyotime P0013B/Selleck B14001). Lysates were centrifuged (12,000 × g, 4°C, 15 min). Ubiquitination assays followed established protocols [55]. Supernatants were incubated overnight at 4°C with Protein A/G Magnetic Beads (HY‐K0202, MCE) pre‐bound to anti‐HA (51064‐2‐AP, Proteintech) or anti‐PFN1 (YN2843, Immunoway). After triple washing with PBS/0.5% Tween‐20, bound proteins were eluted by denaturation in Laemmli buffer. Ubiquitination levels were detected via immunoblotting using anti‐Myc or anti‐Ubiquitin antibodies, the latter identifying total ubiquitin‐conjugated proteins.

### 4.7 Statistical Analysis

Results are expressed as the mean ± SD. All statistical analyses were performed with GraphPad Prism 7.0 software. The Shapiro–Wilk test was used to test the normality of all data obtained from the in vivo study. For comparisons between two groups, analyses were performed using the unpaired Student t‐test (for normally distributed data) or the Mann–Whitney test (for not normally distributed data). Comparisons between multiple groups were performed using one‐way analysis of variance (ANOVA) followed by Tukey's post hoc multiple comparison test or two‐way ANOVA followed by Sidak's post hoc multiple comparison test. When two conditions were considered between groups, two‐way ANOVA with appropriate post‐hoc correction was used. All experiments were performed independently, *p* < 0.05 was considered statistically significant.

## Author Contributions

W‐J.D., Z‐M.D., and X‐D.L. conceived the study concept. X‐D.L., X‐Q.H., X‐Y.H. and Y.Z., performed the experimental studies. Z‐R.W., H‐D.L., H‐N.D., S.W. and X.Z. carried out the data analysis. X‐D.L. wrote the manuscript. W‐J.D., Z‐M.D., and X‐D.L. provided the funding. All authors reviewed the manuscript. X‐D.L., X‐Q.H., X‐Y.H. and Y.Z. contributed equally to this work.

## Conflicts of Interest

The authors declare no conflicts of interest.

## Supporting information




**Supporting File**: advs73882‐sup‐0001‐SuppMat.docx.

## Data Availability

All data needed to evaluate the conclusions in the paper are present in the paper and/or the Supplementary Materials. The high‐throughput transcriptome sequencing data presented in the study are deposited in the GEO under accession codes GSE57345 (https://www.ncbi.nlm.nih.gov/geo/query/acc.cgi?acc = GSE57345) and GSE36074 (https://www.ncbi.nlm.nih.gov/gds/?term = GSE36074). The scRNA‐seq data in this study are based on the dataset available in the GEO under accession code GSE271946 (www.ncbi.nlm.nih.gov/geo/query/acc.cgi?acc = GSE271946).
