# Supplementary material for: F‐Box and Leucine‐Rich Repeat Protein 4 (FBXL4) Maintains Sarcomere Integrity and Cardiac Function by Enhancing K48‐Linked Ubiquitinated Degradation of Profilin‐1 (PFN1)
Source: Adv Sci (Weinh). 2026 Jan 27;13(17):e16702. doi: 10.1002/advs.202516702 (PMC13042929; doi:10.1002/advs.202516702)
Supplement: Supplementary file 1 — Supporting File: advs73882‐sup‐0001‐SuppMat.docx. [file ADVS-13-e16702-s001.docx]

**F-box and leucine-rich repeat protein 4 (FBXL4) Maintains Sarcomere Integrity and Cardiac Function by Enhancing K48-linked Ubiquitinated Degradation of Profilin-1 (PFN1)**

Short title: *FBXL4 ameliorates cardiac hypertrophy and heart failure*

Xingda Li^1#*^, Xueqi He^1#^, Xinyuan Hao^1#^, Yu Zhang^1#^, Xin Zhao^1^, Shuang Wang^1^, Zhenru Wang^1^, Haonan Du^1^, Hongda Li^1^, Lian Yi^2^, Zhimin Du^1,3*^, Weijie Du^4,5,6*^

Affiliations: 1. Institute of Clinical Pharmacy of The Second Affiliated Hospital of Harbin Medical University, State Key Laboratory of Frigid Zone Cardiovascular Diseases (SKLFZCD), Harbin, China.

2. Department of Cardiology, the First Affiliated Hospital of Harbin Medical University, No.23, YouZheng Street, NanGang District, Harbin 150001, Heilongjiang Province, China.

3. State Key Laboratory of Quality Research in Chinese Medicines, Macau University of Science and Technology, Macau, 999078, China.

4. State Key Laboratory of Frigid Zone Cardiovascular Diseases (SKLFZCD), Department of Pharmacology (State Key Laboratory -Province Key Laboratories of Biomedicine-Pharmaceutics of China, Key Laboratory of Cardiovascular Research, Ministry of Education), College of Pharmacy, Harbin Medical University, Harbin 150081, China.

5. Research Unit of Noninfectious Chronic Diseases in Frigid Zone (2019RU070), Chinese Academy of Medical Sciences, Harbin 150081, China.

6. Northern Translational Medicine Research and Cooperation Center, Heilongjiang Academy of Medical Sciences, Harbin Medical University, Harbin, China.

^#^Xingda Li, Xueqi He, Xinyuan Hao, Yu Zhang contributed equally to this study.

^*^Address correspondence to:

Prof. Weijie Du

State Key Laboratory of Frigid Zone Cardiovascular Diseases (SKLFZCD), Department of Pharmacology (State Key Laboratory -Province Key Laboratories of Biomedicine-Pharmaceutics of China, Key Laboratory of Cardiovascular Research, Ministry of Education), College of Pharmacy, Harbin Medical University, Harbin 150081, China.

Tel: +86-86605353;

E-mail: duweijie@hrbmu.edu.cn

Prof. Zhimin Du

Institute of Clinical Pharmacy of The Second Affiliated Hospital of Harbin Medical University, State Key Laboratory of Frigid Zone Cardiovascular Diseases (SKLFZCD), Harbin, China.

Tel: +86-86605353;

E-mail: dzm1956@126.com

Dr. Xingda Li

Institute of Clinical Pharmacy of The Second Affiliated Hospital of Harbin Medical University, State Key Laboratory of Frigid Zone Cardiovascular Diseases (SKLFZCD), Harbin, China.

Tel: +86-86605353;

E-mail: lixingdahmu@163.com

**Supplementary** **Materials and Methods**

**Generation of genetically modified mice**

The *FBXL4* transgenic mice were generated by Cyagen Biosciences Company (China). The *FBXL4* floxed allele was engineered at Cyagen Biosciences (Shanghai, China). The gRNA to FBXL4 gene, the donor vector containing loxP sites, and Cas9 mRNA were co-injected into fertilized mouse eggs to generate targeted conditional knockout offspring. The FBXL4 floxed mice was bred with Myh6-MerCreMer (MCM) lines to generate cardiomyocyte-specific FBXL4 knockout mice. Tamoxifen (T5648, Sigma) dissolved in corn oil was administered intraperitoneally (20 mg/kg·day) for 5 consecutive days to induce recombination activity in Myh6-MCM mice. Genomic DNA was isolated from the mouse tail tip and the genotyping was performed (PD101, Vazyme, China) using the primers as follows:

FBXL4: F1: 5’-CCAAAGCAAACCAAGGTAAATGG-3’ R1: 5’-ATTTCCGTTATTGGCCCCATGAAA-3’

Myh6-Cre：5’ATGACAGACAGATCCCTCCTATCTCC-3’ and 5’-CTCATCACTCGTTGCATCATCGAC-3’;

Myh6-MerCreMer: 5’-TCTATTGCACACAGCAATCCA-3’, 5’-CCAGCATTGTGAGAACAAGG-3’.

FBXL4 conditional knockin mice were generated by Cyagen Biosciences Inc (Guangzhou, China). The gRNA to mouse FBXL4 gene, the donor vector containing “CAG promoter-loxP-PGK-Neo-6*SV40 pA-loxP-Kozak-Mouse FBXL4 CDS-3xFLAG tag-WPRE-BGH pA” cassette, and Cas9 mRNA were co-injected into fertilized mouse eggs to generate targeted conditional knockin offspring. F0 founder animals were identified by PCR followed by sequence analysis, which were bred to wildtype mice to test germline transmission and F1 animal generation. FBXL4 conditional knockin mice were then mated with Myh6-Cre mice. Cardiac-specific FBXL4 overexpression mice were obtained after multiple generations of reproductive crosses. All mice were compared only to non-transgenic or wild-type gender-matched littermates. F3: 5’-CACTTGCTCTCCCAAAGTCGCTC-3’, R1: 5’-GATGGGGAGAGTGAAGCAGAACG-3’, R3: 5’-ATACTCCGAGGCGGATCACAA-3’

**Swimming protocol**

The swimming protocol was initiated with two 10‑minute sessions in pre‑warmed water on day 1, separated by at least four hours. Each day thereafter through day 9, the session duration was progressively increased by 10 minutes, culminating in two 90‑minute sessions on day 9. From days 10 to 20, the mice performed two 90‑minute swimming sessions daily, as previously established. ^[^[^1^](#_ENREF_1)^]^ All subsequent experiments were conducted on day 21.

**Single-cell data collection and processing**

We obtained heart failure single-cell RNA-seq data (GSE271946) from the Single Cell Portal (https://singlecell.broadinstitute.org/single_cell). Preprocessing, quality control, and clustering were performed using Seurat (v5.1.0) in R (v4.3.0; https://www.r-project.org). Low-quality cells were filtered using these thresholds: nCount_RNA between 500 and 90,000, nFeature_RNA between 300 and 10,000, and percent.mt < 5%. Expression values were normalized and scaled, and the top 3,000 variable features were selected. The integrated data were then re-normalized and scaled. Cell clustering was performed at resolution 0.3 and visualized using UAP. Marker genes for each cluster were identified (logFC > 0.25) via the FindAllMarkers function. Cell types were annotated by comparing marker genes with original study annotations and reference markers from the Cell Taxonomy database (<https://ngdc.cncb.ac.cn/celltaxonomy/>).

**GEO database mining**

Raw data in the GSE36074 and GSE57345 dataset was downloaded from the GEO database (https://www.ncbi.nlm.nih.gov/geo/). Limma 3.50.1 package was used to analyze gene expression levels between different samples, such as Normal vs Heart faulure. Genes showing |log2 fold change | > 0.5 and adjusted P value < 0.05 were considered to present differential expression.

**Cell culture and Cell transfection with siRNA**

Cardiomyocytes or HiPSC-derived CMs were isolated from postnatal day 1 mouse hearts. Briefly, hearts were harvested, rinsed with PBS, and minced into pieces before digestion with trypsin (Bl121001; Delta). The supernatant was collected and centrifuged to isolate cells. Cells were initially cultured in DMEM/High Glucose medium (Hyclone) supplemented with 10% fetal bovine serum at 37°C under 5% CO₂ for 1.5 hours, then maintained under the same conditions for continued culture. Two days later, cardiomyocytes were transfected with FBXL4- or PFN1-overexpressing plasmids, using empty vector as a negative control (NC). Plasmid transfections were performed using Lipofectamine 2000 (Invitrogen), and siRNA transfections with X-treme gene siRNA transfection reagent (Roche). The siRNA sequences used are listed below: **Supplementary Table 2.**

**HiPSC-derived CMs culture**

HiPSC-derived cardiomyocytes were maintained on plates coated with 1% fibronectin, with medium replenished every two days. ^[^[^2^](#_ENREF_2)^]^ On day 18, the cells were passaged, seeded into 24-well plates, and transfected with either an FBXL4 overexpression plasmid or siRNA. Subsequently, the cells were treated with angiotensin II for 48 hours and subjected to immunofluorescence staining.

**Quantitative real-time PCR**

Total RNA was extracted from cells subjected to different treatments using Trizol reagent (Invitrogen, Carlsbad, CA). The extracted RNA samples were subsequently subjected to reverse transcription using the High Capacity cDNA Reverse Transcription Kit (Applied Biosystems, Foster City, CA) according to the manufacturer's instructions with a random primer. To quantify the expression levels of the target genes PFN1 and FBXL4, quantitative real-time PCR was performed on an ABI 7500 fast Real-Time PCR system (Applied Biosystems, USA) utilizing SYBR Green PCR Master Mix (Applied Biosystems, Foster City, CA). The reference gene GAPDH was employed as internal control for data normalization. The relative expression levels of PFN1 and FBXL4 mRNA were quantified through the application of the relative quantitative 2^-ΔΔ^ CT method. The primers were designed by Seven/Abcells, Beijing, China, China. The sequences of primers are listed in **Supplementary Table 3.**

**Western blot analysis**
Total protein was extracted using RIPA lysis buffer containing protease inhibitors and subsequently separated by SDS-PAGE before being transferred to polyvinylidene difluoride membranes (Pall Corporation, Mexico, USA). Following this, the membranes were blocked with StartingBlock (Genscript ProBio, Nanjing, China) and probed with the appropriate primary and secondary antibodies. After thorough washing with PBST (phosphate-buffered saline with Tween-20), the membranes were scanned and analyzed using the ODYSSEY machine (LI-COR, America). Antibodies used are listed in **Supplementary Table 4**.

**Construction of adeno-associated virus 9 (AAV9) carrying PFN1 siRNA**

Myh6-Cre and FBXL4-iCKO mice were randomly divided into groups.The adeno-associated virus 9 carrying siRNA for PFN1 (AAV9-siPFN1) and its negative control construct AAV9-siNC were constructed by Cyagen Biosciences Company (China). Mice were given AAV9-siPFN1 or AAV9-siNC (1.04 × 10^11^ viral particles/mL) by intravenous injection through tail vein. After two weeks, mice were subjected to TAC surgery or sham operation for control. Measurements were made six weeks after TAC.

**Construction of adeno-associated virus 9 (AAV9) carrying PFN1 overexpression**

Myh6-Cre and FBXL4-iCKO mice were randomly divided into groups. The adeno-associated virus 9 carrying PFN1 (AAV9-PFN1) and its negative control construct AAV9-siNC were constructed by Cyagen Biosciences Company (China). Mice were given AAV9-siPFN1 or AAV9-siNC (1.1 × 10^11^ viral particles/mL) by intravenous injection through tail vein. The infection was allowed to persist for at least 8 weeks.

**Construction of adeno-associated virus 9 (AAV9) carrying FBXL4**

Myh6-Cre and FBXL4-iCKO mice were randomly divided into groups. The overexpression virus AAV9-FBXL4 and its negative control AAV9-Vector, purchased from Genechem (Shanghai, China), were were administered through the tail vein. Each mouse received AAV9 at a dose of 1×10^11^ viral genomes (vg), and the infection was allowed to persist for at least 4 weeks.

**Transmission Electron Microscopy (TEM)**

Heart tissue was trimmed into small pieces smaller than 1 mm³ and fixed with glutaraldehyde (16537, Electron Microscopy Sciences, USA) to preserve ultrastructure. Post-fixation was treated with 1% citric acid for 2 hours at 4°C, followed by staining with 2% uranium acetate and dehydration with gradient ethanol (50%, 70%, 90%, and 100%) and infiltration with 100% acetone for 2 hours. Pure Epon 812 resin (14120, Electron Microscopy Sciences, USA) was used for embedding and polymerized overnight at 30°C. Ultrathin sections were prepared and collected on Formvar coated copper mesh (FCF100-Cu-UA, Electron Microscopy Sciences, USA), stained with uranium acetate and lead citrate, and then analyzed using a JEM-1200 electron microscope (JEOL Ltd., Tokyo, Japan).

**Chromatin immunoprecipitation (ChIP) assay**

The binding sites of SP1 and FBXL4 were determined using a ChIP assay. For this purpose, formaldehyde was added to the cells to facilitate cross-linking of the target proteins with genomic DNA. Then, cells were digested to obtain lysates, which were then sonicated to achieve genomic DNA of 200–1000 bp fragments. The target proteins and the DNA fragments bound to them were co-immunoprecipitated, purified, and amplified using PCR.

**Dual-Luciferase Reporter Assay**

To construct the FBXL4 expression plasmid, a wild-type (WT) or mutant (MT) promoter fragment of the FBXL4 gene was inserted into the pGL3-basic + PRL-TK vector. SP1 gene was cloned into pcDNA3.1 vector (GENE CREATE, China) for subsequent functional studies. In the dual luciferase reporter assay, HEK293T cells were seeded in 96-well plates and co-transfected with the following plasmid combinations: pGL3-FBXL4-WT + PRL-TK or pGL3-FBXL4-MT + PRL-TK plasmids, along with pcDNA3.1-FBXL4 or the corresponding negative control plasmids, using Lipofectamine 2000 reagent (according to the manufacturer's instructions). Cells were harvested 48 hours after transfection and renilla luciferase activity was measured using a dual luciferase reporter gene detection system (E1910, Promega, Madison, WI, USA). Binding sites between SP1 and the FBXL4 promoter predicted using the Jaspar system list in **Supplementary Table 5.**

**Immunohistochemistry**

NMCMs were seeded onto laminin-coated coverslips for 24 hours transfection of siFBXL4 or FBXL4 overexpressing plasmids followed by Ang II stimulation for another 24 hours. Then, the cells were fixed with 4% formaldehyde, permeabilized with 0.1% Triton X-100 in PBS for 45 min, and stained with α-actinin (A7811, Sigma; diluted at 1:300) at 4°C overnight. Next, the cells were incubated with a Daylight 594 goat anti-mouse antibody at room temperature for 1 h. The cells were incubated with DAPI for 10 min before immunofluorescence capture. Immunofluorescence was visualized under a fluorescence microscope (Carl Zeiss, 37081). Quantification of cell surface area was achieved by measuring 30 randomly selected cells from 4 independent experiments, and the averaged values were used for analysis. Cell surface area was measured using Image-Pro Plus 6.0 software. Moreover, Frozen section staining of cardiac muscle tissue was assessed using wheat germ agglutinin (WGA; L4895, Sigma). Images from these procedures were acquired with a microscope (BX53, OLYMPUS) and analyzed using ImageJ software.

**Reactive Oxygen Species (ROS) Staining**

Measurement of total intracellular ROS levels was performed with the DCFH-DA probe (S0033S, Biyuntian, China). At the expected confluent state, cardiomyocytes were treated with 10 μM DCFH-DA in serum-free DMEM medium and incubated (37°C, 20 min, protected from light). Subsequent imaging was carried out using a confocal laser scanning microscope (FV300, Olympus, Japan).

**Measurement of Oxygen Consumption Rate (OCR)**

Cardiomyocyte oxygen consumption rate (OCR) was measured using a high-resolution respirometer (Oxygraph-2k; Oroboros Instruments). Following the acquisition of the basal OCR, sequential titrations of oligomycin (Omy), the uncoupler FCCP, rotenone (Rot), and antimycin A (Ama) were performed as described previously 36740831. Mitochondrial ATP production was quantified by subtracting the OCR after the addition of 1 μM oligomycin from the basal OCR. ^[^[^3^](#_ENREF_3)^]^

**Histological staining**

The hearts were fixed in 4% paraformaldehyde at 4°C for a period longer than 24 hours. After removing excess liquid with filter paper, the tissues were dehydrated and embedded in paraffin. Then, staining was performed using the Masson Tricolor Staining Kit or HE Staining Kit according to the manufacturer's instructions. The infarct area was determined using Image Pro Plus software, and the results were quantified.

**Supplementary Table 1. Detailed information of the human heart samples.**

| Subject | Diagnosis | Age  (years) | Gender | LVEF% | LVEDd  (mm) |
| --- | --- | --- | --- | --- | --- |
| 1 | Normal | 50 | Male | 68 | 48 |
| 2 | Normal | 52 | Male | 62 | 45 |
| 4 | Normal | 45 | Male | 70 | 52 |
| 5 | DCM | 57 | Male | 26 | 89 |
| 6 | DCM | 52 | Female | 31 | 69 |
| 7 | DCM | 43 | Male | 17 | 67 |
| 8 | DCM | 55 | Female | 27 | 66 |

LVEF, left ventricular eject fraction; LVEDd, left ventricular end-diastolic dimension; IVSD, interventricular septal thickness at diastole. DCM, dilated cardiomyopathy.

**Supplementary Table 2. The sequence of siRNA.**

| Gene | Forward (5’-3’) | Reverse (5’-3’) |
| --- | --- | --- |
| Mus-si-FBXL4 | CCCAAAUCUACAAGACUUAAAdTdT | UUUAAGUCUUGUAGAUUUGGGdTdT |
| Mus-si-PFN1-1  Mus-si-PFN1-2  Mus-si-PFN1-3 | GGGACUCACUGCUGCAAGAdTdT  GGGACUCACUGCUGCAAGAdTdT  GAUCAACAAGAAAUGUUAUdTdT | UCUUGCAGCAGUGAGUCCCdTdT  UCUUGCAGCAGUGAGUCCCdTdT  AUAACAUUUCUUGUUGAUCdTdT |
| Mus-si-SP1 | GCAGAAAGAGGGAGAGCAAdTdT | UUGCUCUCCCUCUUUCUGCdTdT |
| Mus-si-FXOA1 | UGGAAGGGCAUGAGAGCAAdTdT | UUGCUCUCAUGCCCUUCCAdTdT |
| Mus-si-FXOA2  Homo-si-FBXL4-1  Homo-si-FBXL4-2  Homo-si-FBXL4-3 | GCACAAGCGAGGUGGCCUAdTdT  CCGAATTAGTACGCCTTGAAT  CGAATTAGTACGCCTTGAATT  GCCAGGACTATGTGGAACTTA | UAGGCCACCUCGCUUGUGCdTdT  ATTCAAGGCGTACTAATTCCGG  AATTCAAGGCGTACTAATTCCG  TAAGTTCCACATAGTCCGGC |

**Supplementary Table 3. Gene primer sequence.**

| Geen | Primer sequence (5’-3’) |
| --- | --- |
| Homo-FBXL4-F | ACCTATCATCCCGGAGCAGT |
| Homo-FBXL4-R | TAAACTGGCGAGCTTGGGAA |
| Mmu-FBXL4-F | UCAGGUUGCUGGAGGAUAATT |
| Mmu-FBXL4-R  Mmu-ANP-F  Mmu-ANP-R  Mmu-BNP-F  Mmu-BNP-R  Mmu-β-MHC-F  Mmu-β-MHC-R | UUAUCCUCCAGCAACCUGATT  TCGTCTTGGCCTTTTGGCT  TCCAGGTGGTCTAGCAGGTTCT  AGGGAGAACACGGCATCATT  GACAGCACCTTCAGGAGAT  GTGAAGGGCATGAGGAAGAGT  AGGCCTTCACCTTCAGCTGC |
| Mmu-FOXA1-F | GCCAAGACATTCAAGCGCAG |
| Mmu-FOXA1-R | TGCTGGTTCTGGCGGTAATA |
| Mmu-FOXA2-F | ACATACCGACGCAGCTACAC |
| Mmu-FOXA2-R | ATCTTGTTGGGGCTCTGCTG |
| Mmu-SP1-F | ATGGACAGGTCAGTTGGCAG |
| Mmu-SP1-R | AGGCAATGGGTGTTAGGGTG |
| Mmu-FBXL4(Promotor)-F | GGCTGCCTTATCTGGCTTCA |
| Mmu-FBXL4(Promotor)-R | CCCTCCCACAATCCTTCTC |

**Supplementary Table 4. Antibodies used for Western bolt/Immunoprecipitation.**

| Antibody | Dilution ratio | Company | Lot.No. |
| --- | --- | --- | --- |
| FBXL4 | WB 1:200 | Immunoway | YN4163 |
| FBXL4(IP) | IP 1:50 | Santa Cruz | sc-390102 |
| PFN1  PFN1(IP) | WB 1:200  IP 1:50 | Immunoway  Immunoway | YN2843  YN2843 |
| β-MHC | WB 1:5000 | Sigma-Aldrich | SAB4700663 |
| p-ERK1/2 (T202/Y204) | WB 1:1000 | Abways | F137601 |
| Ubiquitin | WB 1:1000 | Cell signaling technology | 10201-2-AP |
| ERK1/2 | WB 1:1000 | Selleckchem | F1716 |
| ANP  HSP70  PFN1(Try129)  PFN1(Ser138)  MYH6  MYBPC4  cTNT | WB1:1000  WB1:2000  WB1:100  WB1:100  WB 1:1000  WB 1:1000  WB 1:1000 | Proteintech  Proteintech  Thermofisher  Thermofisher  Proteintech  Zenbio  Zenbio | 27426-1-AP  10995-1-AP  PA5-143753  PA5-143752  22281-1-AP  671202  222118 |
| GAPDH | WB 1:5000 | Abways | AB0037 |
| SP1 | WB 1:500 | Proteintech | 21962-1-AP |
| HA(IP) | IP 1:50 | Proteintech | 51064-2-AP |
| HA | WB 1:1000 | Proteintech | 51064-2-AP |
| Flag (IP) | IP 1:50 | Proteintech | 66008-4-Ig |
| Flag | WB 1:1000 | Proteintech | 66008-4-Ig |
| Myc | WB 1:000 | Proteintech | 60003-2-Ig |
| Myc | IP 1:50 | Proteintech | 60003-2-Ig |
| β-actin | WB 1:5000 | Abways | F131607 |
| α-actinin | IF 1:200 | Sigma | A7811 |

**Supplementary Table 5. Binding sites between SP1 and the FBXL4 promoter predicted using the Jaspar system.**

| Matrix ID | Name | Score | Relative score | Sequence ID | Start | End | Strand | Predicted sequence |
| --- | --- | --- | --- | --- | --- | --- | --- | --- |
| [**MA0079.2**](https://jaspar.elixir.no/matrix/MA0079.2) | MA0079.2.SP1 | 13.213753 | 0.9640745 | FBXL4 | 1973 | 1982 | + | ccccgcctcc |
| [**MA0079.2**](https://jaspar.elixir.no/matrix/MA0079.2) | MA0079.2.SP1 | 13.213753 | 0.9640745 | FBXL4 | 1990 | 1999 | + | ccccgcctcc |
| [**MA0079.2**](https://jaspar.elixir.no/matrix/MA0079.2) | MA0079.2.SP1 | 12.629657 | 0.9493644 | FBXL4 | 222 | 231 | - | cccctcccac |
| [**MA0079.2**](https://jaspar.elixir.no/matrix/MA0079.2) | MA0079.2.SP1 | 12.023177 | 0.9340907 | FBXL4 | 1968 | 1977 | + | ccccgccccg |
| [**MA0079.2**](https://jaspar.elixir.no/matrix/MA0079.2) | MA0079.2.SP1 | 12.023177 | 0.9340907 | FBXL4 | 1985 | 1994 | + | ccccgccccg |
| [**MA0079.2**](https://jaspar.elixir.no/matrix/MA0079.2) | MA0079.2.SP1 | 11.048319 | 0.9095394 | FBXL4 | 190 | 199 | - | ccccttctcc |
| [**MA0079.2**](https://jaspar.elixir.no/matrix/MA0079.2) | MA0079.2.SP1 | 10.649176 | 0.8994874 | FBXL4 | 198 | 207 | - | cccttcctcc |
| [**MA0079.2**](https://jaspar.elixir.no/matrix/MA0079.2) | MA0079.2.SP1 | 9.750317 | 0.8768501 | FBXL4 | 1951 | 1960 | + | ccccgccctt |
| [**MA0079.2**](https://jaspar.elixir.no/matrix/MA0079.2) | MA0079.2.SP1 | 9.739942 | 0.8765888 | FBXL4 | 659 | 668 | + | ctctgccccc |
| [**MA0079.2**](https://jaspar.elixir.no/matrix/MA0079.2) | MA0079.2.SP1 | 9.547203 | 0.87173486 | FBXL4 | 2305 | 2314 | + | cgccgcctcc |
| [**MA0079.2**](https://jaspar.elixir.no/matrix/MA0079.2) | MA0079.2.SP1 | 8.6904955 | 0.8501592 | FBXL4 | 1945 | 1954 | + | ccctggcccc |

**Supplementary Figures and Figure legends**


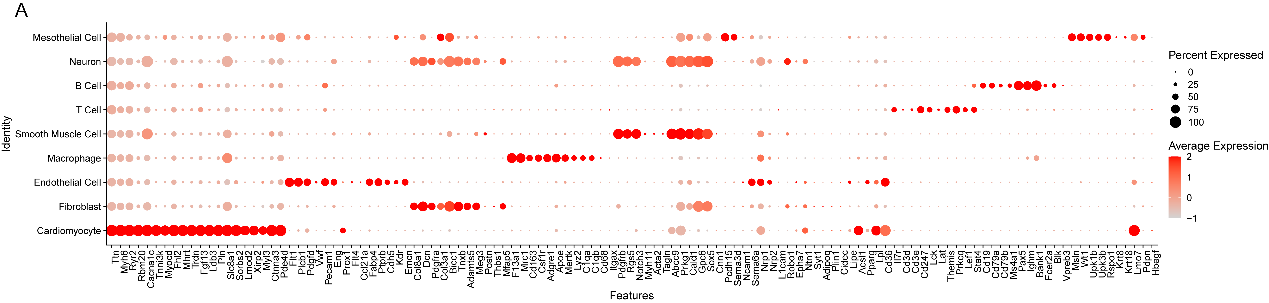


**Supplementary Figure 1. Expression of cell markers for each cell type in single cell sequencing data.**  **A)** Dot map showing differentially expressed cell marker genes in each cell type in GSE255054.

**
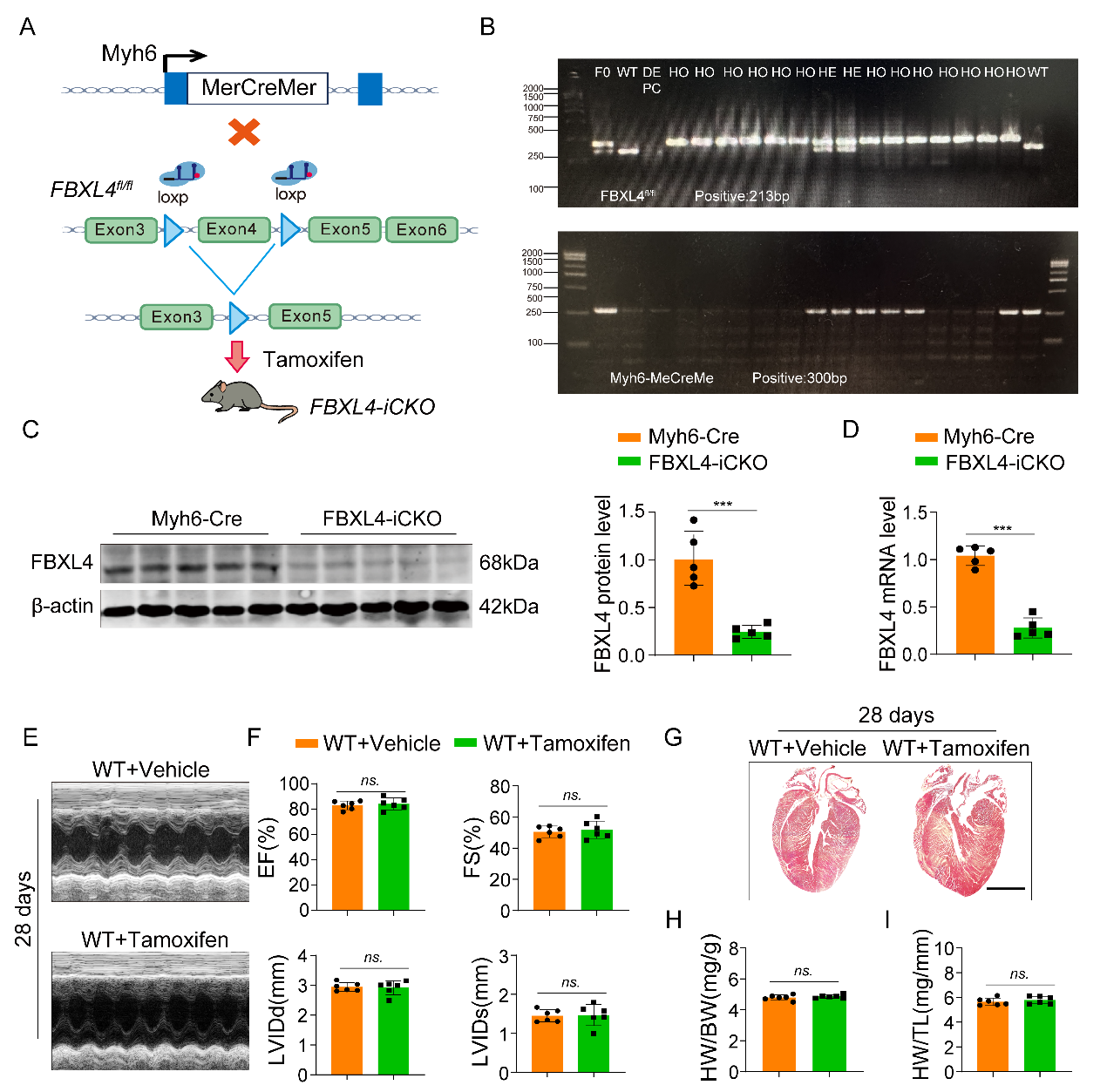
**

**Supplementary Figure 2. Construction of inducible FBXL4 cardiac-specific knockout mice. A, B)** A simple schematic diagram and identification for the generation of FBXL4- iCKO mice. **C, D)** FBXL4 protein and mRNA levels in Myh6-Cre and iCKO mice, n = 5 mice/group. **E, F)** Representative M-mode echocardiography of the left ventricle and statistical data of EF%, FS%, LVIDd and LVIDs. n=6/group. **G)** Representative 4-chamber H&E staining of the hearts. Scale bar=2 mm. **H, I)** Heart weight normalized body weight (HW/BW) and heart weight normalized to tibia length (HW/TL) of WT+Vehicle vs WT+Tamoxifen, n=6/group. n represents the number of independent samples per group*.* The data were shown as means ± SD. *n.s* indicates no significance. **p* < 0.05, ***p* < 0.01, and ****p* < 0.001. Statistical differences were assessed by unpaired t test with Welch’s correction (C, D, F, H, I).


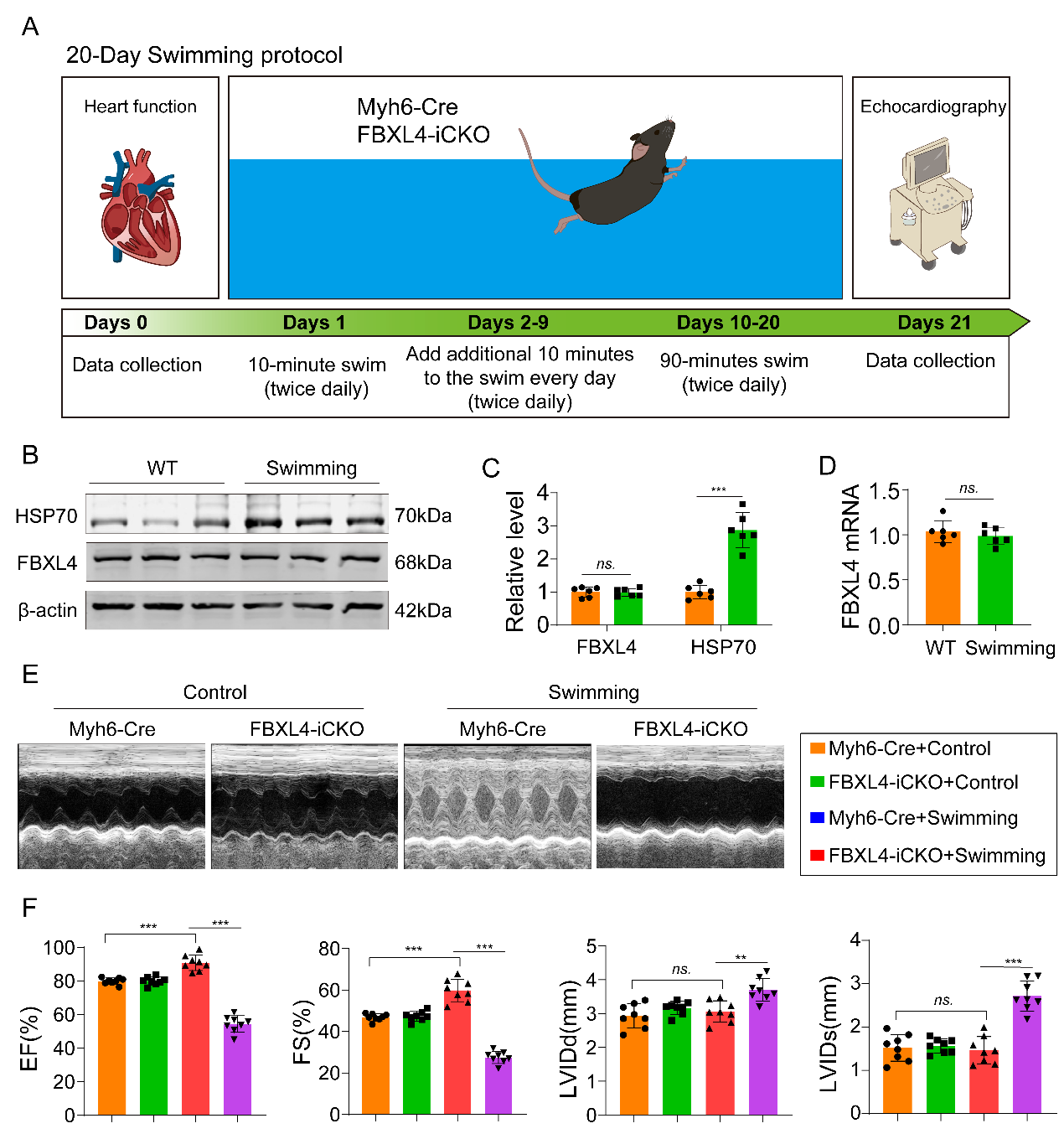


**Supplementary Figure 3. Cardiomyocyte specific deletion of FBXL4 is specific to pathological cardiac remodeling. A)** Diagram depicting swim training protocol and timing of echocardiography and heart tissue harvest. **B-D)** Protein and mRNA levels of HSP70 and FBXL4 in left ventricular tissues of mice after 4-weeks sham or swimming exercise, n = 6 hearts/group. **E, F)** Representative M-mode echocardiography and doppler echocardiography of the left ventricle and statistical data of EF%, FS%, LVIDd and LVIDs, n=8. n represents the number of independent samples per group*.* Data were shown as mean ± SD. **p* < 0.05, ***p* < 0.01, and ****p* < 0.001. *n.s* indicates no significance. Statistical differences were assessed by unpaired t test with Welch’s correction (C, D), two-way ANOVA followed by Sidak post hoc multiple comparisons test (F).


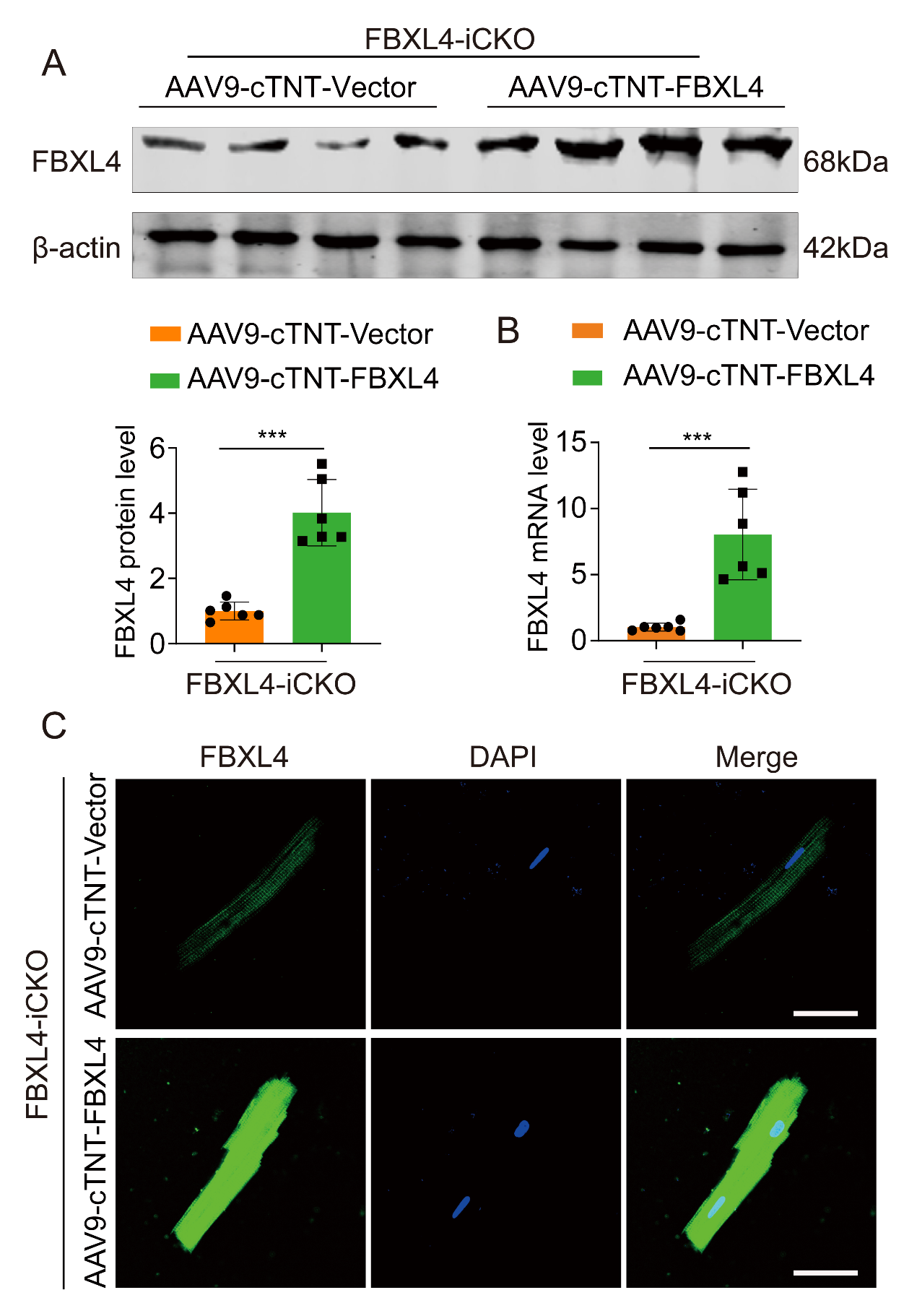


**Supplementary Figure 4. Verification of the transfection efficiency of AAV9-FBXL4 in the hearts of mice. A, B)** FBXL4 protein and mRNA level in mouse myocardial tissues after AAV9-cTNT-FBXL4 administration in FBXL4-iCKO mice, n = 6 mice/group. **C)** IF staining showed that verification of successful transfection of AAV9 carrying vector and FBXL4 in FBXL4-iCKO mice, n = 3 mice/group. Scale bar, 50 μm. n represents the number of independent samples per group. n represents the number of independent samples per group*.* The data were shown as means ± SD. ****p* < 0.001. Statistical differences were assessed by unpaired t test with Welch’s correction (A, B).


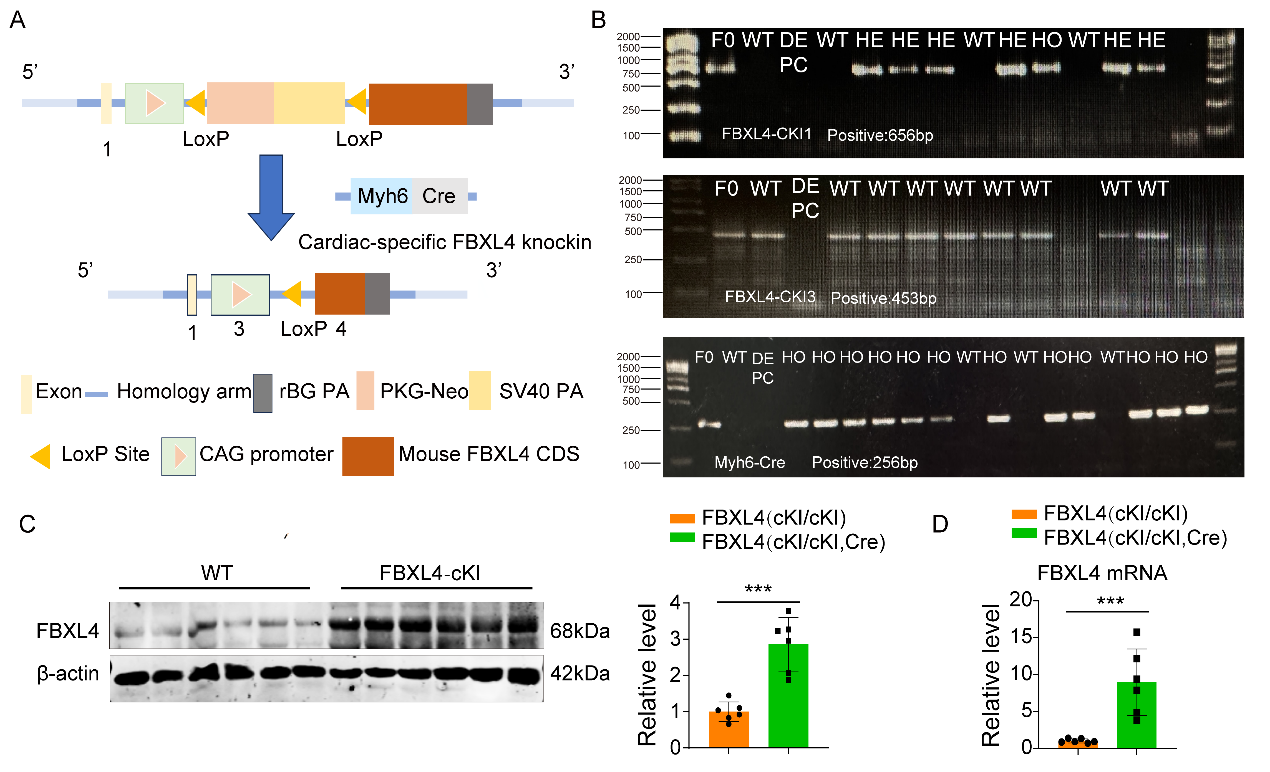


**Supplementary Figure 5. The construction of FBXL4 cardiac-specific knockin (cKI) mice.** **A, B)** A simple schematic diagram and identification for the generation of FBXL4 -cKI mice. **C, D)** FBXL4 protein and mRNA levels in FBXL4^fl/fl^ and FBXL4-cKI mice (n=6). n represents the number of independent samples per group*.* The data were shown as means ± SD. ****p* < 0.001. Statistical differences were assessed by unpaired t test with Welch’s correction (C, D).


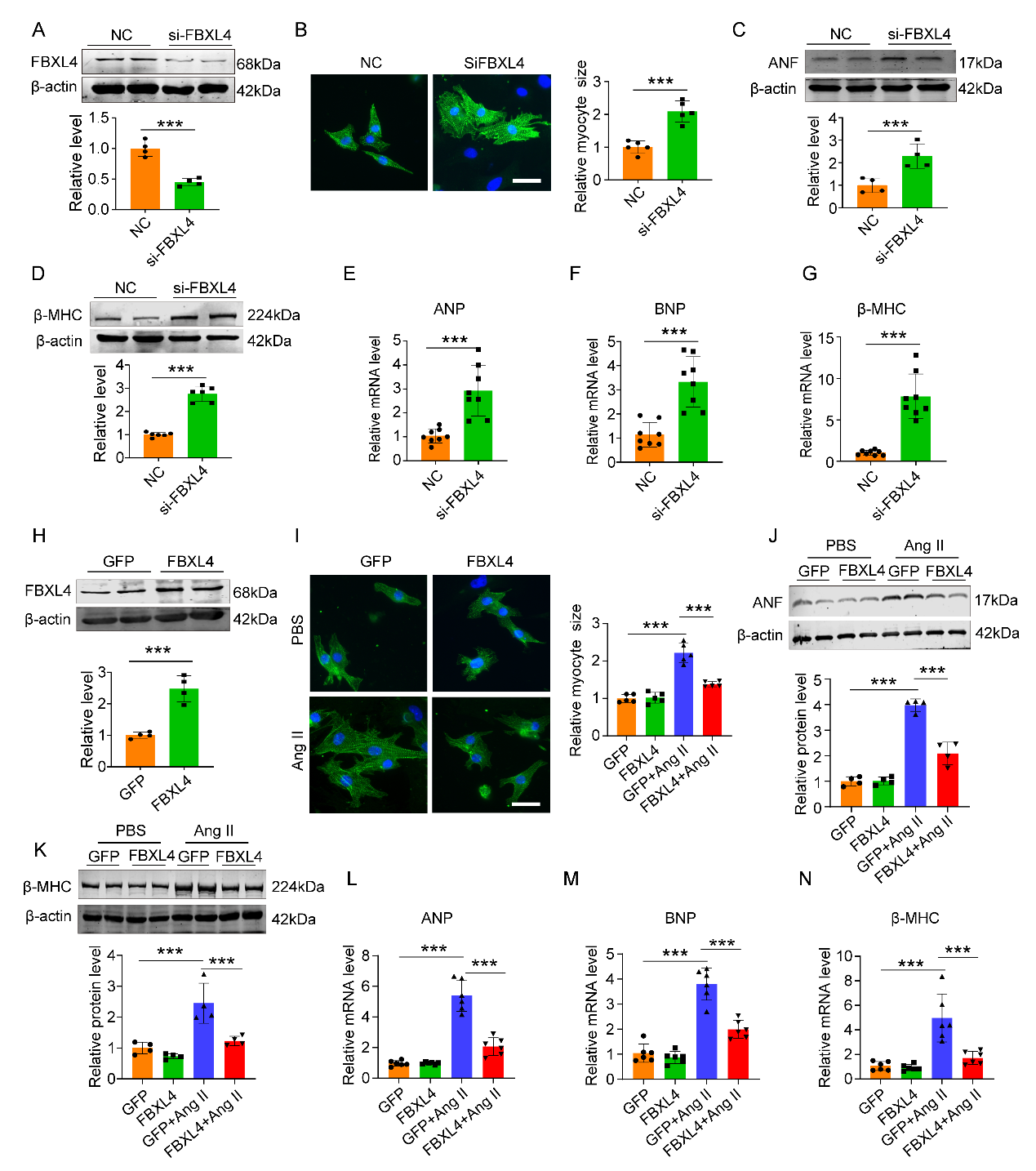


**Supplementary Figure 6. FBXL4 attenuates Ang II-induced cardiomyocyte hypertrophy in *vitro*.** **A)** Verification of the efficacy of FBXL4 siRNA (siFBXL4) in silencing FBXL4 expression at the protein levels (n = 4). NC is a negative control siRNA. **B)** Effects of siFBXL4 on cardiomyocyte size treated with 48 hours (at least 50 cells counted per experiment, n = 5; green represents α-actinin; blue indicates nuclei; scale bar, 50 μm). **C)** Upregulation of ANF protein (n = 4) after transfection of siFBXL4 in cardiomyocytes. **D)** Upregulation of β-MHC protein (n = 6) after transfection of siFBXL4 in cardiomyocytes. **E-G)** Upregulation of ANP, BNP and β-MHC mRNA (n = 8) after transfection of siFBXL4 in cardiomyocytes. **H)** Verification of the efficacy of FBXL4 plasmid in FBXL4 overexpression at the protein levels (n = 4). GFP is a negative control FBXL4. **I)** Overexpression of FBXL4 significantly diminished the enlarged cardiomyocyte size induced by Ang II (1 μM) for 48h without affecting it under control conditions (treated with PBS) (at least 50 cells counted per experiment, n = 5; green represents α-actinin; blue indicates nuclei, scale bar, 50 μm). **J)** Overexpression of FBXL4 reduced the protein (n = 4) levels of ANP in cardiomyocytes. **K)** Overexpression of FBXL4 decreased the protein (n = 4) levels of β-MHC in cardiomyocytes. **L-N)** Overexpression of FBXL4 reduced the mRNA (n = 6) levels of ANP, BNP and β-MHC mRNA in cardiomyocytes. n represents the number of independent samples per group. Data were shown as mean ± SD. **p* < 0.05, ***p* < 0.01, and ****p* < 0.001. Statistical differences were assessed by unpaired t-test with Welch’s correction (B, C, D, E, F, G, H), two-way ANOVA followed by Sidak post hoc multiple comparisons test (I, J, K, L, M, N)


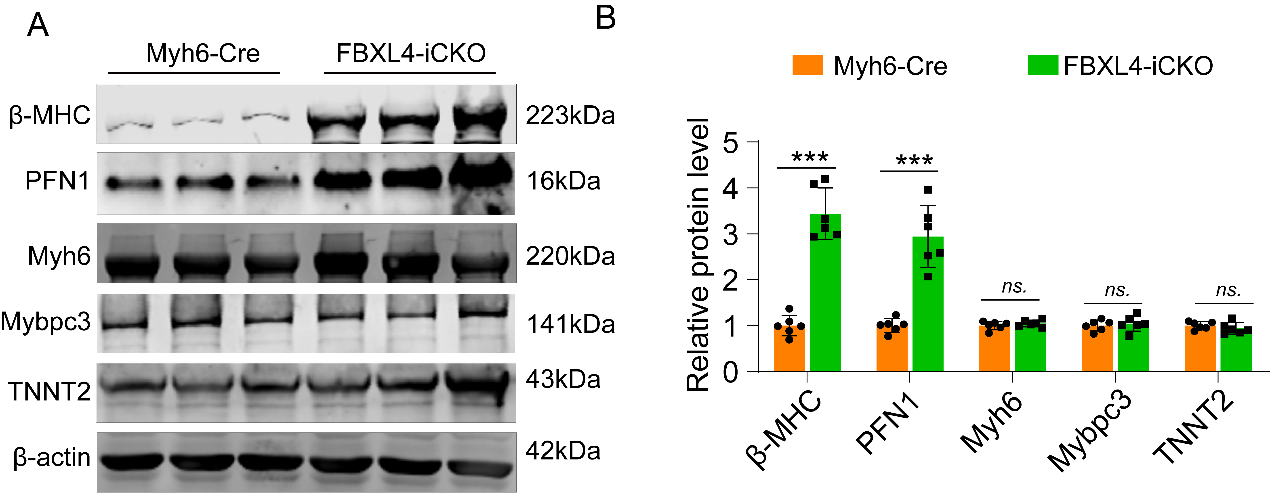


**Supplementary Figure 7. FBXL4 deficiency did not affect the expression of MYH6, Mybpc3, and TNNT2. A, B)** Representative immunoblotting analysis and quantification of β-MHC, PFN1, Myh6, Mybpc3 and TNNT2 protein levels in the Myh6-Cre and FBXL4-iCKO group, n=6. n represents the number of independent samples per group. Data were shown as mean ± SD. *n.s* indicates no significance. ****p* < 0.001. Statistical differences were assessed by unpaired t test with Welch’s correction (B).


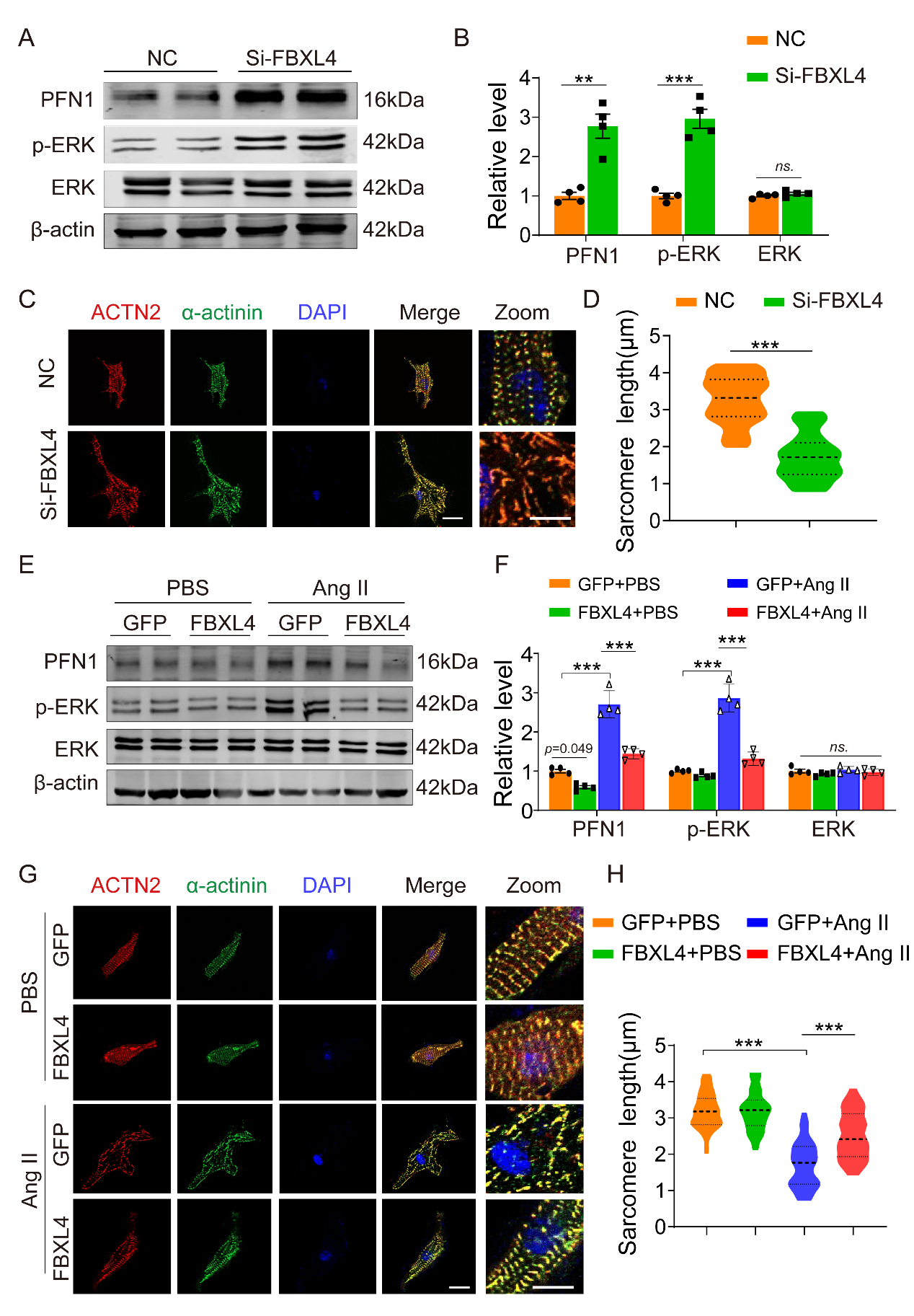


**Supplementary Figure 8. FBXL4 ameliorates cardiomyocyte hypertrophy via PFN1 and sarcomeric remodelling *in vitro*.** **A, B)** Representative immunoblotting analysis and quantification of PFN1, pERK1/2 and ERK1/2 protein levels in the siFBXL4 and NC group (n=4). **C, D)** Representative images and quantification of myofibrillar disarray in NMCMs by staining with ACTN2 (red), α-actinin (green), and DAPI (blue). Scale bar, 20 µm. Zoomed images show a higher magnification. Scale bar, 1 µm. At least 10 cells were counted in each group from 6 independent experiments. **E, F)** Representative immunoblotting analysis and quantification of PFN1, pERK1/2 and ERK1/2 protein levels under Ang II-induced cardiomyocytes hypertrophy in the FBXL4 and GFP group (n=4). **G, H)** Representative images and quantification of myofibrillar disarray in NMCMs by staining with ACTN2 (red), α-actinin (green), and DAPI (blue). Scale bar, 20 µm. Zoomed images show a higher magnification. Scale bar, 1 µm. At least 10 cells were counted in each group from 6 independent experiments. n represents the number of independent samples per group. Data were shown as mean ± SD. *n.s* indicates no significance. **p* < 0.05, ***p* < 0.01, and ****p* < 0.001. Statistical differences were assessed by unpaired t test with Welch’s correction (B, D), two-way ANOVA followed by Sidak post hoc multiple comparisons test (F, H).


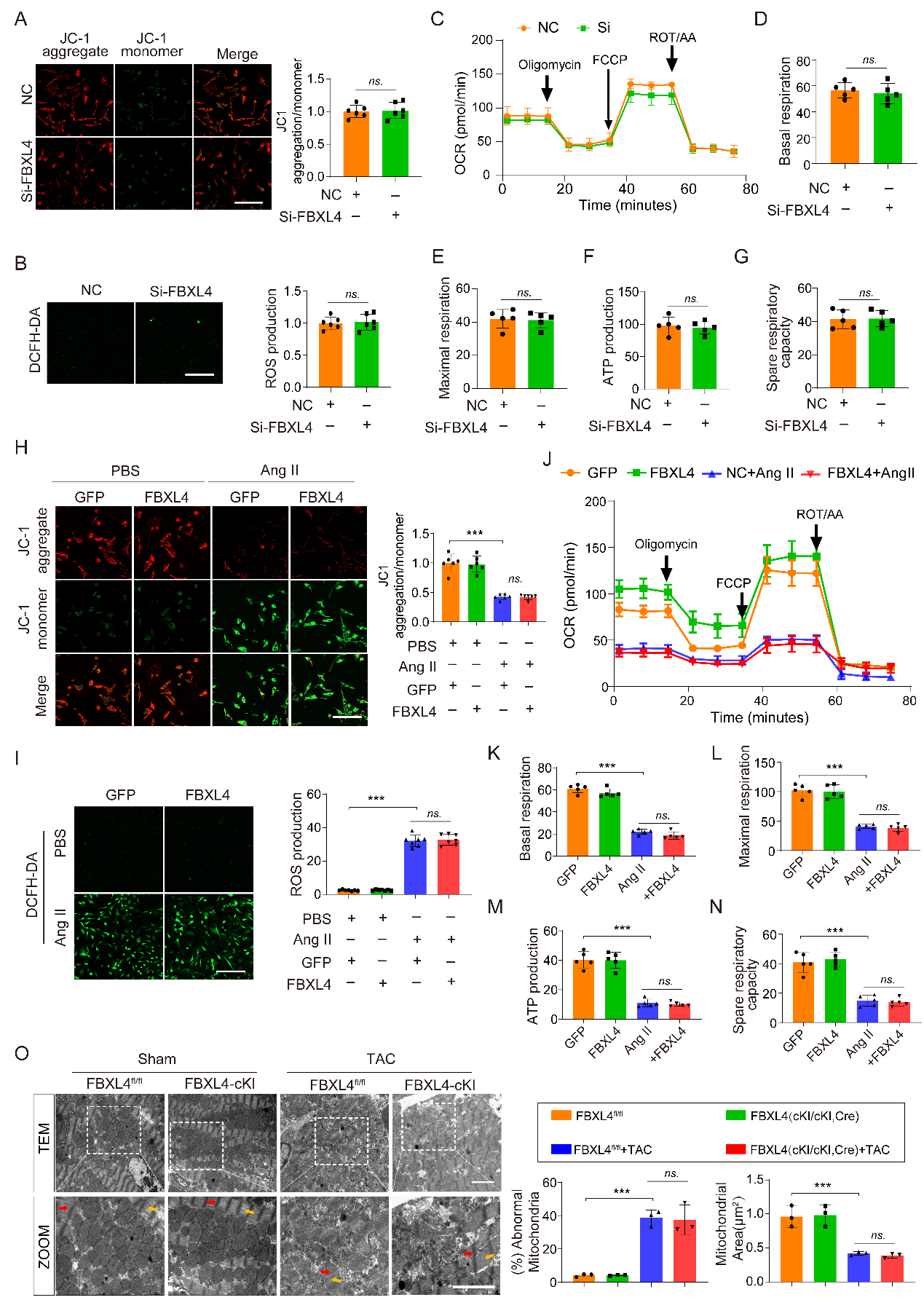


**Supplementary Figure 9. The cardioprotective role of FBXL4 is independent of mitochondrial pathways A)** JC-1 staining images and statistical analysis measuring mitochondrial membrane potential (MMP) in NMCMs after treatment with NC or FBXL4-SiRNA for 48 hours (n = 5; scale bar = 50 μm). **B)** DCFH-DA staining images measuring ROS levels in NMCMs after treatment with NC or FBXL4-SiRNA for 48 hours (n = 5; scale bar = 50 μm). **C)** Analysis of O₂ consumption in NMCMs treated with NC or FBXL4-SiRNA for 48 hours (n = 5). **D)** Statistical analysis of basal respiration (n = 5). **E)** Statistical analysis of maximal respiration (n = 5). **F)** Statistical analysis of mitochondrial ATP production (n = 5). **G)** Statistical analysis of spare respiration capacity (n = 5). NMCMs after treatment with NC or FBXL4-SiRNA for 48 hours in A, B, C, D, E, F, G). **H)** JC-1 staining images and statistical analysis measuring mitochondrial membrane potential (MMP) in (n = 5; scale bar = 50 μm). **I)** DCFH-DA staining images measuring ROS levels in NMCMs after treatment with Ang II, GFP or FBXL4 for 48 hours (n = 5; scale bar = 20 μm) (n = 5; scale bar = 50 μm). **J)** Analysis of O₂ consumption in NMCMs treated with NC or FBXL4-SiRNA for 48 hours (n = 5). **K)** Statistical analysis of basal respiration (n = 5). **L)** Statistical analysis of maximal respiration (n = 5). **M)** Statistical analysis of mitochondrial ATP production (n = 5). **N)** Statistical analysis of spare respiration capacity (n = 5). (NMCMs after treatment with Ang II, GFP or FBXL4 for 48 hours in H, I, J, K, L, M, N). **O)** Representative images and statistical data of TEM from mouse hearts after sham or TAC surgery in FBXL4^fl/fl^ and FBXL4 cKI mice (n=4, scale bar, 100 μm and 20 µm.). n represents the number of independent samples per group. Data were shown as mean ± SD. **p* < 0.05, ***p* < 0.01, and ****p* < 0.001. *n.s* indicates no significance. Statistical differences were assessed by unpaired t test with Welch’s correction (A, D, B, E, F, G), two-way ANOVA with Bonferroni’s post-hoc test was used to determine the difference among the treatment groups and different time points (C, J), two-way ANOVA followed by Sidak post hoc multiple comparisons test (H, I, K, L, M, N, O).


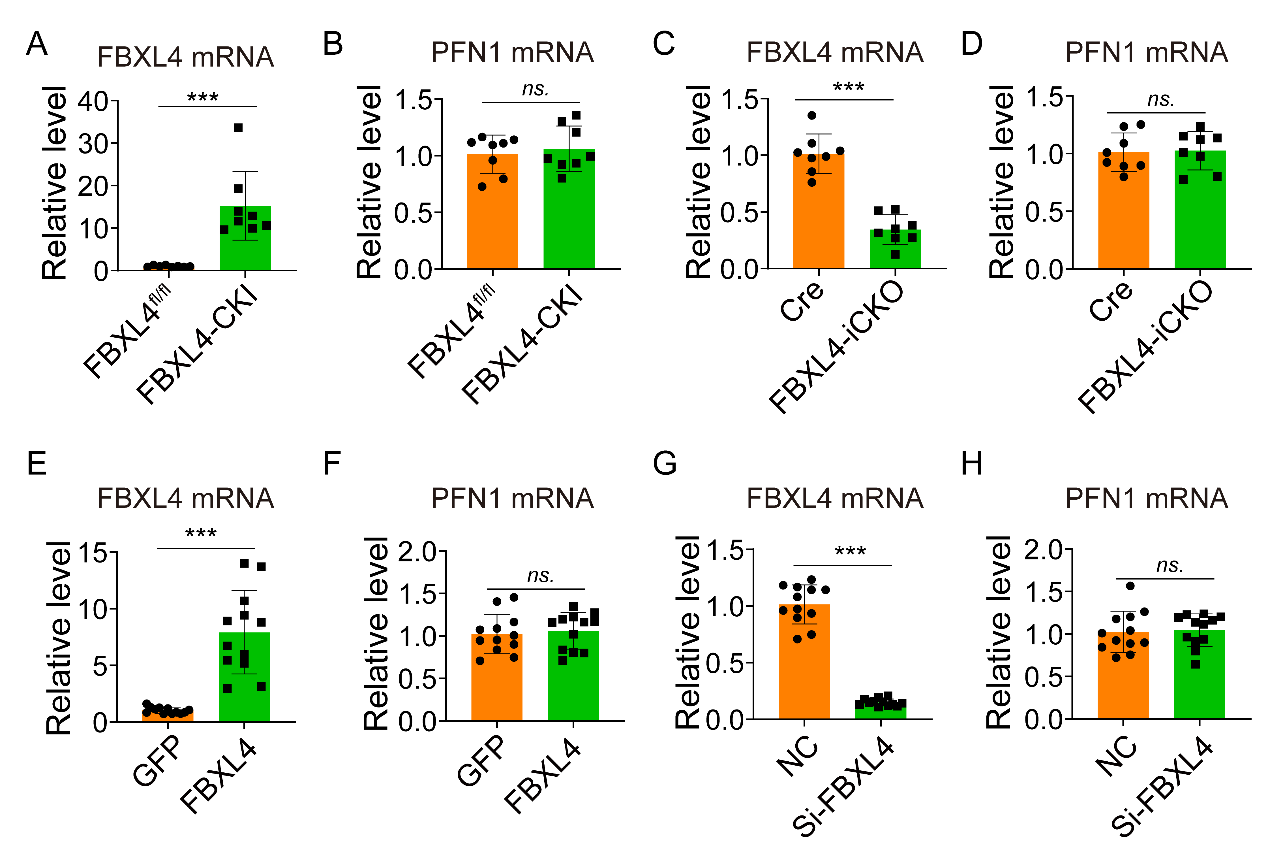


**Supplementary Figure 10. FBXL4 depletion or overexpression did not alter PFN1 mRNA levels either *in vivo* or *vitro*.** **A, B)** qRT-PCR analysis of FBXL4 and PFN1 mRNA expression in FBXL4^fl/fl^ and FBXL4-TG mice, n=8. **C, D)** qRT-PCR analysis of FBXL4 and PFN1 mRNA expression in Myh6-Cre and FBXL4-iCKO mice, n=8. **E, F)** qRT-PCR analysis of FBXL4 and PFN1 mRNA expression in cardiomyocytes transfected with GFP and PFN1, n=12. **G, H)** qRT-PCR analysis of FBXL4 and PFN1 mRNA expression in cardiomyocytes transfected with NC and Si-FBXL4, n=12. n represents the number of independent samples per group. Data were shown as mean ± SD. ****p* < 0.001. *n.s* indicates no significance. Statistical differences were assessed by unpaired t test with Welch’s correction (A, B, C, D, E, F, G, H).


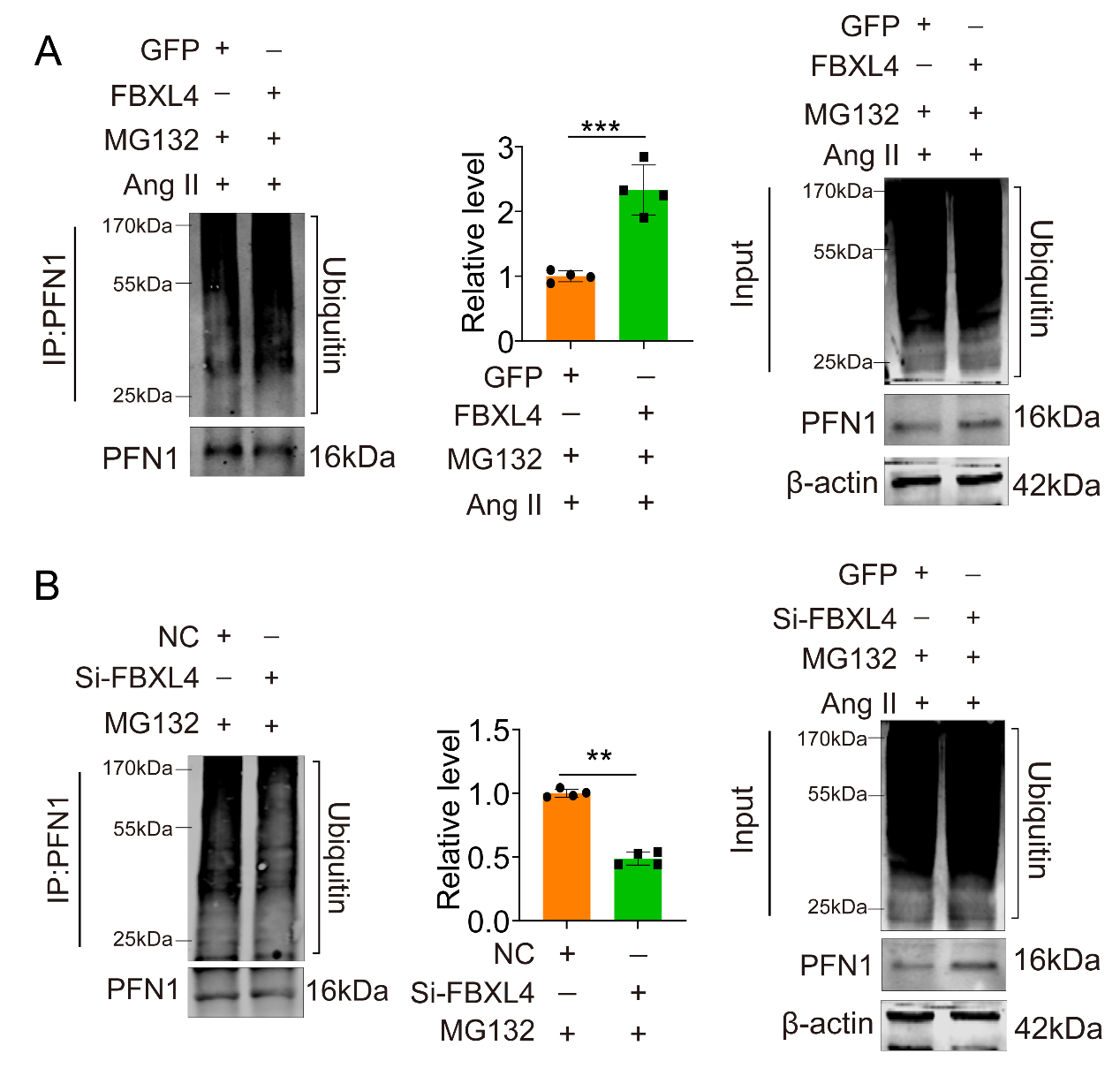


**Supplementary Figure 11. FBXL4 regulates the ubiquitination of PFN1 *in* *vitro*. A)** Lysates were extracted from NMCMs that were transfected with PFN1 and then treated with MG132 for 12h, and immunoprecipitated with anti-PFN1 antibody. Western blot analysis was used to detect the ubiquitination levels of PFN1 with anti-ubiquitin (left), n=4. Input (Western blot analysis of each protein with corresponding antibody) (right). **B)** Lysates were extracted from NMCMs that were transfected with siFBXL4 and then treated with MG132 for 12h, and immunoprecipitated with anti-PFN1 antibody. Western blot analysis was used to detect ubiquitination levels of PFN1 with anti-ubiquitin(left). n=4. Input (Western blot analysis of each protein with corresponding antibody) (right). n represents the number of independent samples per group. Data were shown as mean ± SD. **p* < 0.05, ***p* < 0.01, and ****p* < 0.001. Statistical differences were assessed by unpaired t test with Welch’s correction (A, B).


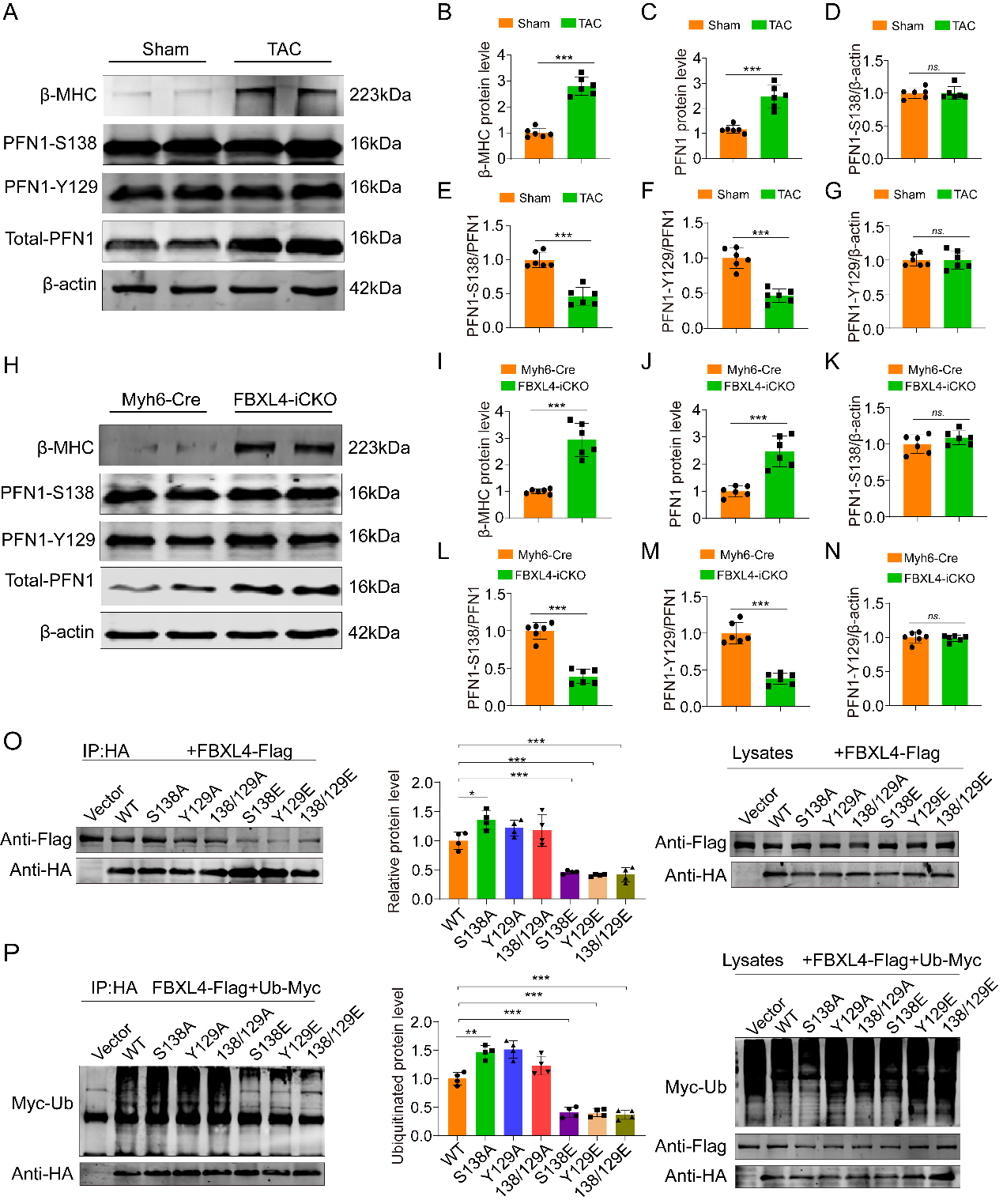


**Supplementary Figure 12. FBXL4 targets PFN1 for ubiquitin-mediated degradation independently of phosphorylation.** **A-G)** Western blot analysis was performed to assess the expression levels of β-MHC, phosphorylated PFN1 at Ser138 (pS138), phosphorylated PFN1 at Tyr129 (pY129), total PFN1, and β-actin, as well as the ratios of pS138 to total PFN1 and pY129 to total PFN1 in Sham and TAC groups. β-actin serves as a loading control. n = 6. **H-N)** Western blot analysis was performed to assess the expression levels of β-MHC, phosphorylated PFN1 at Ser138 (pS138), phosphorylated PFN1 at Tyr129 (pY129), total PFN1, and β-actin, as well as the ratios of pS138 to total PFN1 and pY129 to total PFN1 in Myh6-Cre and FBXL4-iCKO groups. β-actin serves as a loading control. n = 6. **O)** Co-immunoprecipitation (IP) of Flag-FBXL4 and HA-PFN1 in HEK293T cells transfected with the indicated constructs (Vector, WT, S138A, Y129A,138/129A, S138E, Y129E, 138/129E). Immunoblots (IB) show specific pull-down of FBXL4 by PFN1 using anti-HA conjugated beads compared to WT, with input confirming protein expression levels. Quantification of Flag-FBXL4/HA-PFN1 protein normalized (middle). n = 4. **P)** HEK293T cells overexpressing Flag-FBXL4 and Myc-Ub were transfected with the indicated plasmid combinations to measure the ubiquitination of HA-S138A, -Y129A, -138/129A, -S138E, -Y129E, -138/129E. Quantification of Myc-Ub/HA-PFN1 protein normalized (middle). n = 4. n represents the number of independent samples per group. Data were shown as mean ± SD. **p* < 0.05, ***p* < 0.01, and ****p* < 0.001. Statistical differences were assessed by unpaired t test with Welch’s correction (B, C, D, E, F, G, I, J, K, L, M, N), one-way ANOVA analysis followed by Tukey’s post-hoc multiple comparison test (O, P).


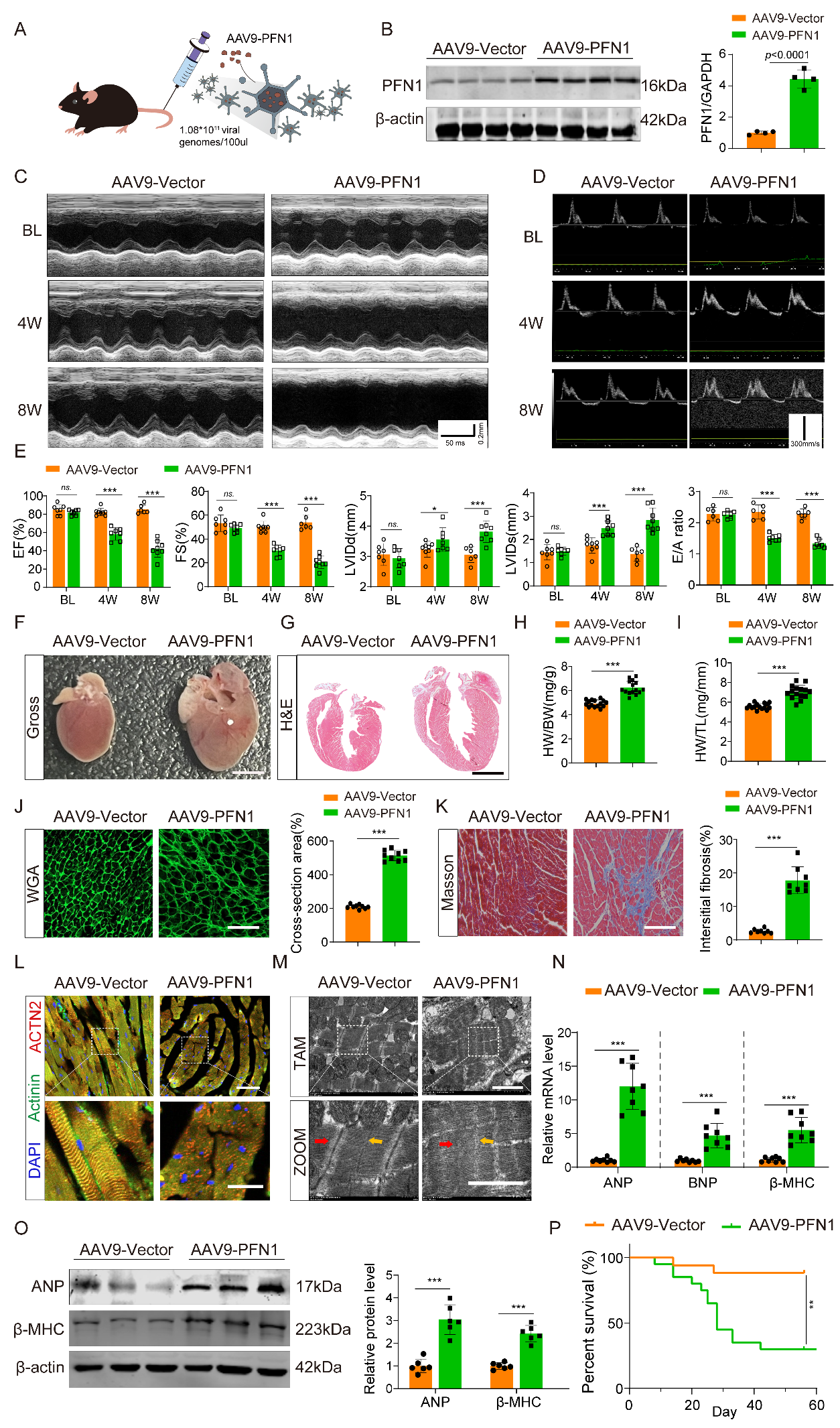


**Supplementary Figure 13. PFN1 is a critical inducer for cardiac hypertrophy and heart failure in mice**. **A)** Diagram of AAV9 (adeno-associated virus 9) carrying PFN1 virus injection.  **B)** Protein level of PFN1 in the hearts of mice, n=4. **C-E)** Representative M-mode echocardiography of the left ventricle and statistical data of EF%, FS%, LVIDd, LVIDs and E/A ratio in AAV9-cTnT-Vector *vs.* AAV9-cTnT-PFN1 mice at baseline, day 28, and day 56, n=7-8. **F, G)** Representative images of heart size photographed with a stereomicroscope and HE staining of heart sections (scale bar, 2 mm). **H, I)** Heart weight (HW)/body weight (BW) and HW/tibia length (TL) ratios in the different groups, n=15/group. **J)** Representative images and statistics of WGA staining, 2-3 sections/mouse, Scale bar=100 μm, n=4. **K)** Representative images and statistics of Masson’s trichrome staining, 3 sections/mouse, Scale bar=100 μm, n=4 mice. **L)** Representative IF images of Z-disc organization and F-actin architecture, stained with DAPI (blue), ACTN2 (red), and α-actinin (green), 3 sections/mouse, n=4 mice. Scale bar=100 μm. Scale bars: upper, 100 µm; lower, 20 µm. **M)** Representative transmission electron microscopy (TEM) analysis of sarcomere structure in AAV9-cTnT-Vector *vs.* AAV9-cTnT-PFN1. n=3/group. The red and yellow arrows indicate Z-disc and M-band, respectively. Scale bars, 20 µm. **N)** qRT-PCR analysis of ANP, BNP and β-MHC in cardiac tissues. n=7-8. **O)** Representative western blot image and analysis of ANP and β-MHC in cardiac tissues. n=6. **P)** Survival curve of AAV9-Vector (n=17) *vs.* AAV9-PFN1 mice (n=18). n represents the number of independent samples per group. Data are shown as mean ± SD. *n.s* indicates no significance. **p* < 0.05, ***p* < 0.01, and ****p* < 0.001. Statistical differences were assessed by two-way ANOVA with Sidak’s multiple comparisons test (E), unpaired t test with Welch’s correction (H, I, J, K, N, O), Kaplan–Meier analysis with the log‐rank Mantel‐Cox test (P).


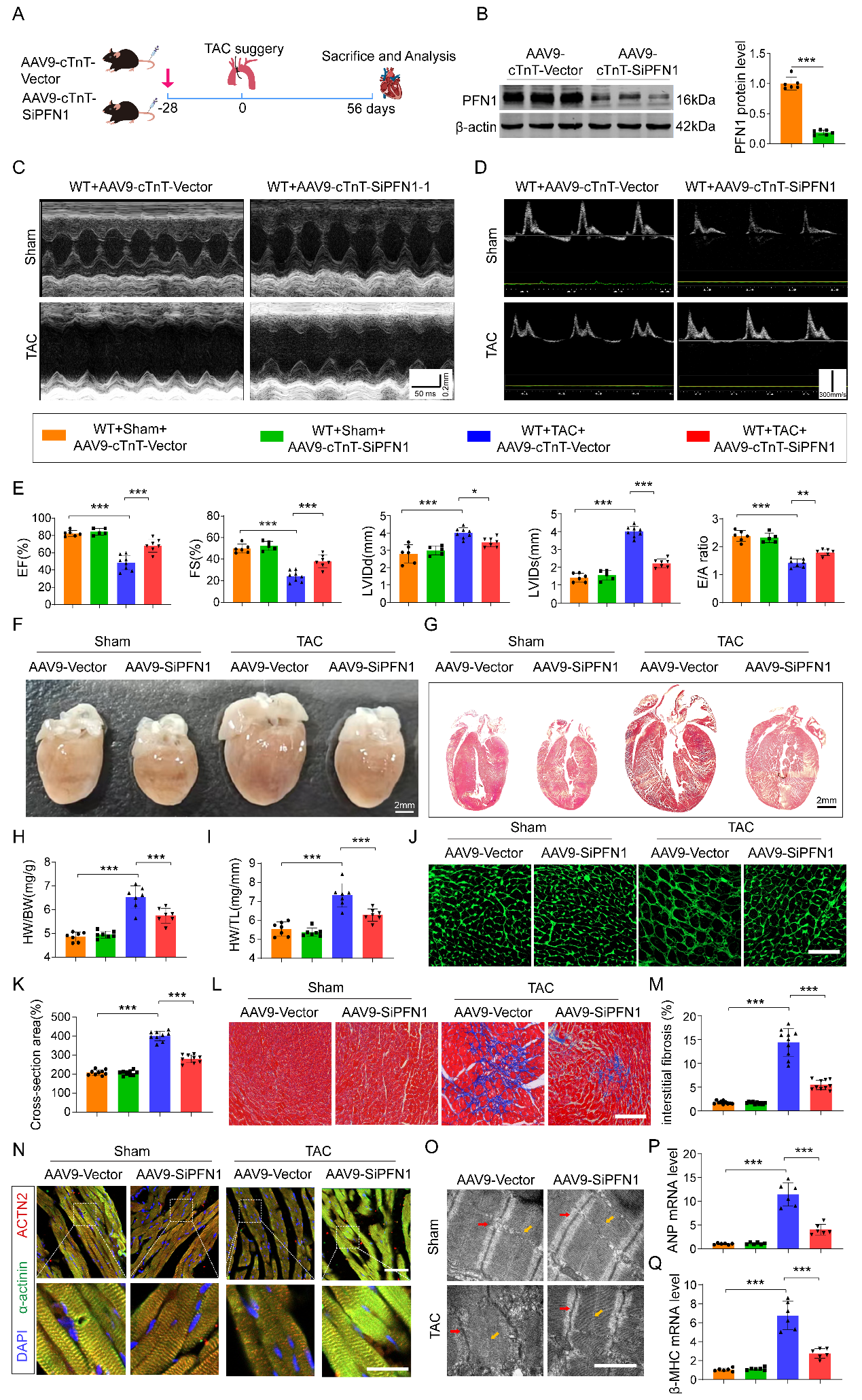


**Supplementary Figure 14. Cardiac-specific PFN1 deficiency attenuates cardiac hypertrophy** **A)** Schematic diagram depicting the experimental strategy for AAV9- cTNT-SiPFN1 and AAV9-cTNT-Vector in the TAC mice. **B)** Protein level of PFN1 in the hearts of mice, n=4. **C-E)** Representative M-mode echocardiography and doppler echocardiography of the left ventricle and statistical data of EF%, FS%, LVIDd, LVIDs and E/A ratio n=6-10/group. **F, G)** Representative images of heart size photographed with a stereomicroscope and HE staining of heart sections (scale bar, 2 mm). **H, I)** Heart weight normalized to body weight and tibia length (HW/BW) (HW/TL), n=15/group. **J, K)** Representative images and statistics of WGA staining (2-3 sections/mouse, Scale bar=100 μm, n=4). **L, M)** Representative images and statistics of Masson’s trichrome staining, 3 sections/mouse, Scale bar=100 μm, n=4 mice. **N)** Representative IF images of Z-disc organization and F-actin architecture, stained with DAPI (blue), ACTN2 (red), and α-actinin (green), 3 sections/mouse, n=4 mice. Scale bars: upper, 100 µm; lower, 20 µm. **O)** Representative TEM analysis of sarcomere structure in AAV9-Vector or AAV9-SiPFN1 treated with WT and WT+TAC mice. The red and yellow arrows indicate Z-disc and M-band, respectively. Scale bars, 20 μm. n=3/group. **P, Q)** qRT-PCR analysis of ANP and β-MHC in cardiac tissues. n=6. n represents the number of independent samples per group. Data were shown as mean ± SD. **p* < 0.05, ***p* < 0.01, and ****p* < 0.001. *p* values obtained via unpaired t test with Welch’s correction (B), two-way ANOVA followed by Sidak post hoc multiple comparisons test (E, H, I, K, M, P, Q).


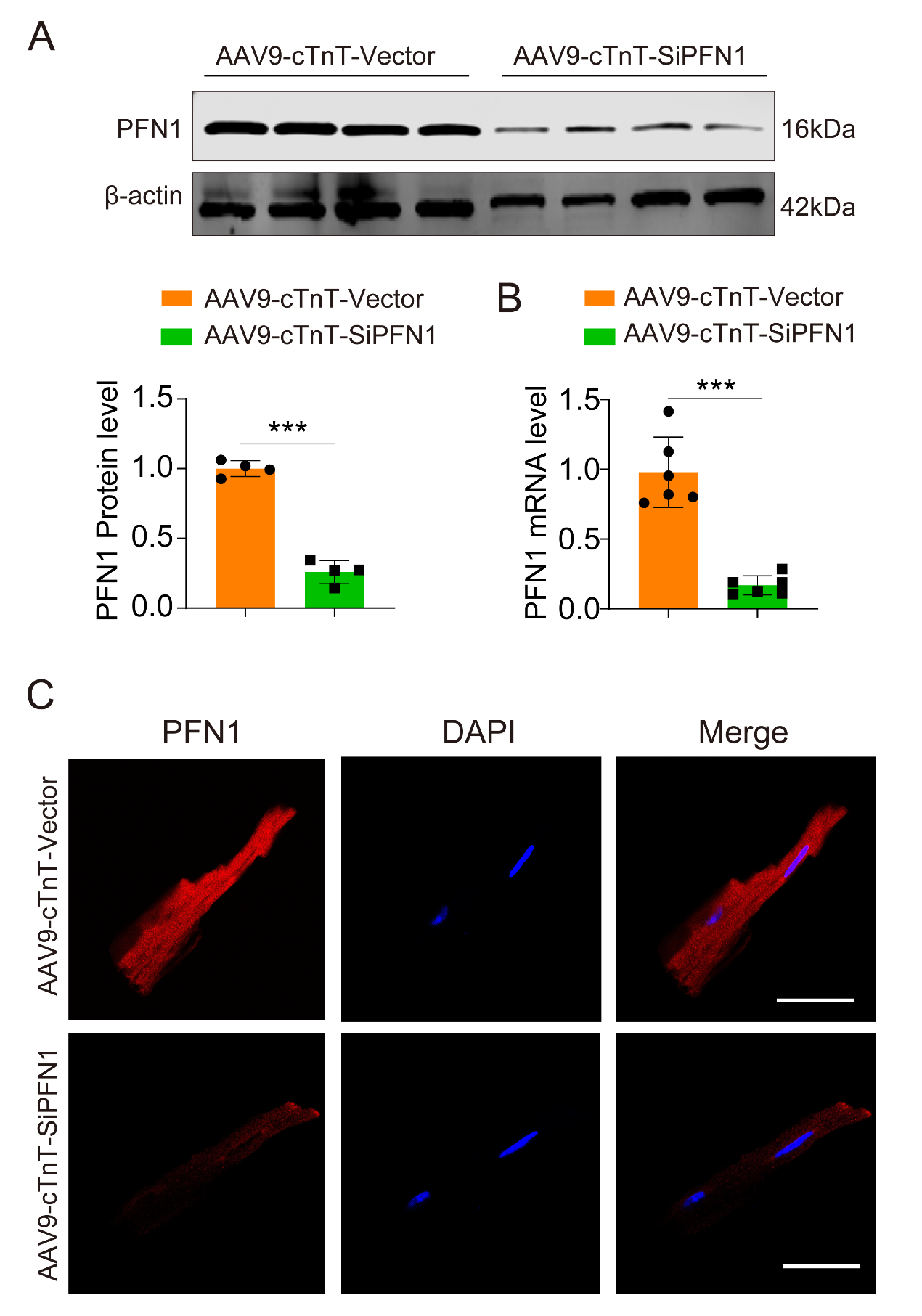


**Supplementary Figure 15. Verification of the knocking down efficiency of AAV9-carrying the siRNA for PFN1 (AAV9-siPFN1) in the hearts of mice.** **A, B)** PFN1 protein and mRNA levels in mouse myocardial tissues after AAV9-siPFN1 administration, n = 4-6 mice/group. **C)** Immunofluorescence staining of adult cardiomyocytes from AAV9-cTNT-Vector and AAV9-cTNT-SiPFN1, stained with PFN1 (red) and DAPI (blue), n = 3 mice/group, Scale bar, 50 μm. n represents the number of independent samples per group. Data were shown as mean ± SD. ****p* < 0.001. Statistical differences were assessed by unpaired t test with Welch’s correction (A, B).


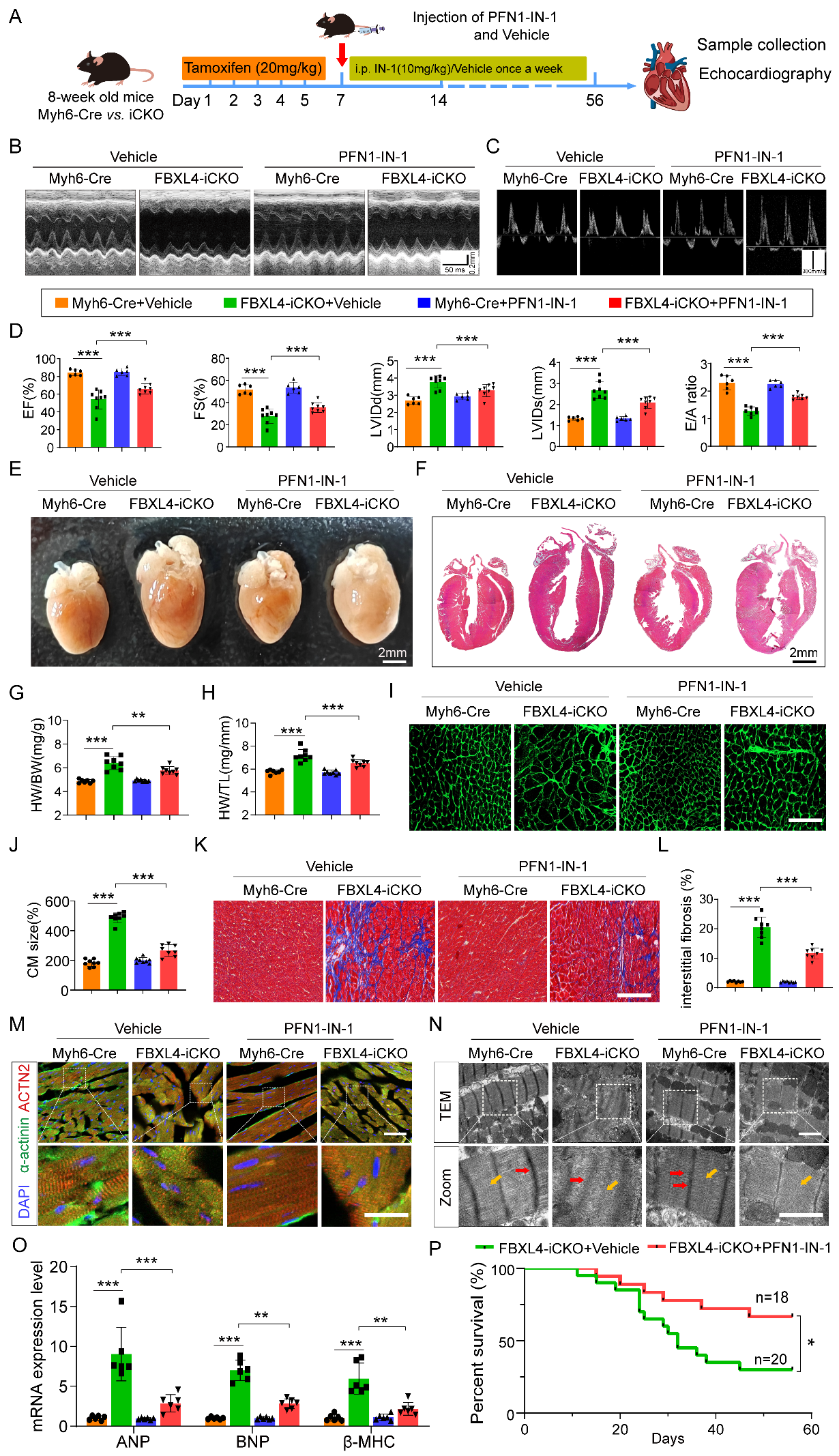


**Supplementary Figure 16. Pharmacological inhibition of PFN1 partially rescues the HF in FBXL4-iCKO Mice.** **A)** Adult FBXL4-iCKO mice and Myh6-Cre control mice were administered tamoxifen via daily injections for 5 consecutive days to induce recombination. Subsequently, the mice received weekly intraperitoneal injections of PFN1-IN-1 or Vehicle for 7 weeks until harvesting. **B-D)** Representative echocardiographic images of M-mode and mitral valve pulse-wave Doppler of EF%, FS%, LVIDd, LVIDs and E/A ratio in different group mice. n=6-8/group. **E, F)** Gross morphology and HE staining of the hearts (scale bar=2 mm). **G, H)** Heart weight (HW)/body weight (BW) and HW/tibia length (TL) ratios in the different groups, n=10/group. **I, J)** Representative images and statistics of WGA staining (12 sections/mouse Scale bar=100 μm). n=8. **(K, L)** Masson’s trichrome staining (Scale bar=100 μm). n=8. **M)** Representative IF images of Z-disc organization and F-actin architecture in AAV9-Vehicle or PFN1-IN-1 treated with FBXL4-iCKO mice, stained with DAPI (blue), ACTN2 (red), and α-actinin (green). 3 sections/mouse, n=4 mice. Scale bar=100 μm. Scale bars: upper, 100 µm; lower, 20 µm. **N)** Representative TEM image of sarcomere structure in AAV9-Vehicle or PFN1-IN-1 treated with FBXL4-iCKO mice. The red and yellow arrows indicate Z-disc and M-band, respectively. Scale bars: upper, 100 µm; lower, 20 µm. n=3. **O)** qRT-PCR analysis of ANP and β-MHC in cardiac tissues, n=6. **P)** Survival curve of individual experimental groups. (FBXL4-iCKO+Vehicle, n=20; FBXL4-iCKO+PFN1-IN-1, n=18). n represents the number of independent samples per group. Data are shown as mean ± SD. **p* < 0.05, ***p* < 0.01, and ****p* < 0.001. Statistical differences were assessed by two-way ANOVA followed by Sidak post hoc multiple comparisons test (D, G H, J, L, O), Kaplan–Meier analysis with the log‐rank Mantel‐Cox test (P).

**
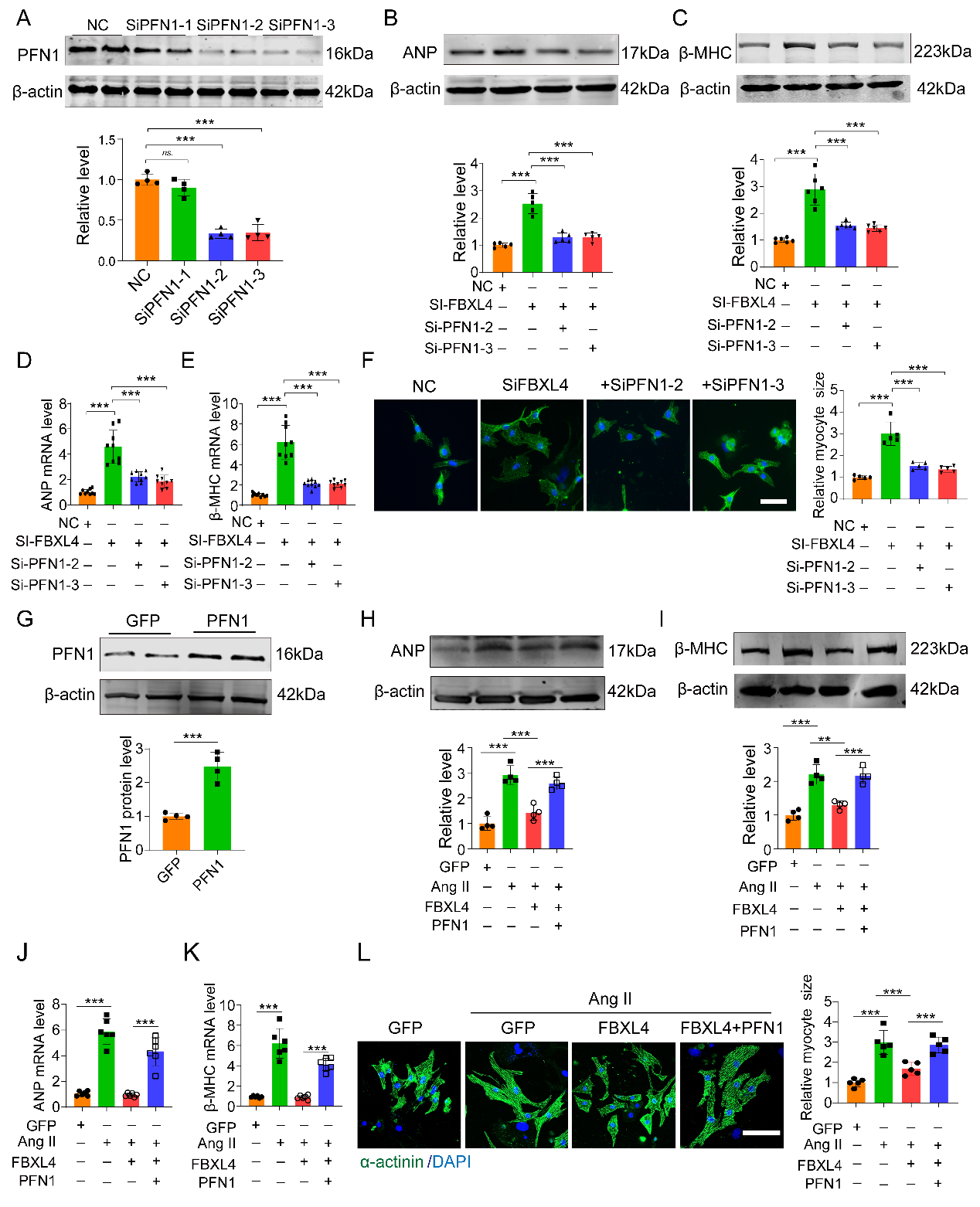
**

**Supplementary Figure 17. FBXL4 modulates cardiomyocytes hypertrophy via PFN1 *in vitro*.** **A)** Verification of the efficacy of PFN1 siRNA (siPFN1) in silencing PFN1 expression at the protein levels (n = 4). **B-C)** ANP and β-MHC protein levels of (n = 4) after co-transfection of SiFBXL4 and SiPFN1-2/SiPFN1-3 in cardiomyocytes. **(D-E)** ANP and β-MHC mRNA levels of (n = 9) after co-transfection of siFBXL4 and SiPFN1-2/SiPFN1-3 in cardiomyocytes. **F)** Effects of SiFBXL4 and SiPFN1-2/SiPFN1-3 co-transfection on cardiomyocyte size treated with 48 hours (at least 50 cells counted per experiment, n = 5; green represents α-actinin; blue indicates nuclei; scale bar, 100 μm). **G)** Verification of the efficacy of PFN1 plasmid in PFN1 overexpression at the protein levels (n = 4). GFP is a negative control PFN1. **H, I)** ANP and β-MHC protein levels of PFN1 and FBXL4 co-transfection in Ang II-induced cardiomyocyte hypertrophy (n = 4). **J, K)** ANP and β-MHC mRNA levels of PFN1 and FBXL4 co-transfection in Ang II-induced cardiomyocyte hypertrophy (n = 6). **L)** Representative images and quantitative data of PFN1 and FBXL4 co-transfection in Ang II-induced cardiomyocyte hypertrophy (at least 50 cells counted per experiment, n = 5; green represents α-actinin; blue indicates nuclei；scale bar, 50 μm). n represents the number of independent samples per group. Data are shown as mean ± SD. **p* < 0.05, ***p* < 0.01, and ****p* < 0.001. Statistical differences were assessed by one-way ANOVA followed by the Dunn post hoc multiple comparisons test (A), two-way ANOVA followed by Sidak post hoc multiple comparisons test (B, C, D, E, F), unpaired t test with Welch’s correction (G), two-way ANOVA followed by Sidak post hoc multiple comparisons test (H, I, J, K, L).


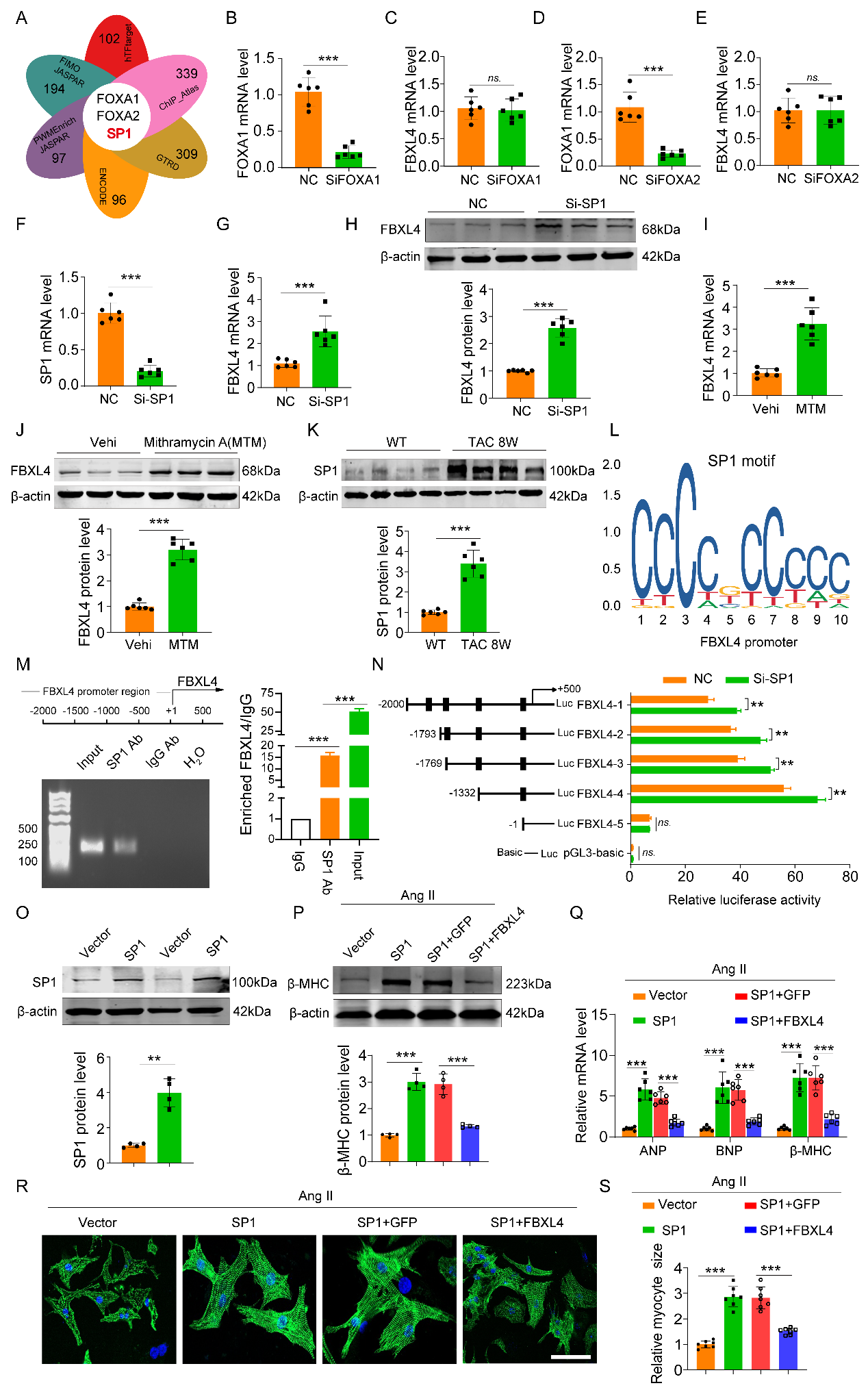


**Supplementary Figure 18. SP1 transcriptionally represses FBXL4 expression during cardiac hypertrophy.** **A)** The Venn diagram illustrates FBXL4’s potential targets as predicted by six databases: hTFtarget, KonckTF, ENCODE, CHEA, TRRUST, GTRD, JASPAR. **B)** Knockdown efficiency of FOXA1 in cardiomyocytes by its siRNA. n=6. **C)** The mRNA levels of FBXL4 in cardiomyocytes transfected with si-FOXA1. n=6. **D)** Knockdown efficiency of FOXA2 in cardiomyocytes by its siRNA. n=6. **E)** The mRNA levels of FBXL4 in cardiomyocytes transfected with si-FOXA2. n=6. **F)** The mRNA levels of SP1 in cardiomyocytes transfected with si-SP1. n=6. **G)** The mRNA levels of FBXL4 in cardiomyocytes transfected with si-SP1. n=6. **H)** The protein levels of FBXL4 in cardiomyocytes transfected with si-SP1. n=6. **I, J)** FBXL4 mRNA and protein levels in WT mice treated with SP1 antagonist, Mithramycin A (MTM) by qRT-PCR and western blot. n=6. **K)** Protein levels of SP1 in left ventricular tissues of hypertrophic mice after 8-weeks sham or TAC operation, n = 6 hearts/group. **L)** SP1 was predicted as a transcription factor of FBXL4. **M)** ChIP-qPCR analysis was performed with SP1 or IgG antibody to determine the binding ability of SP1 to FBXL4 promoter in NMCMs, n=3. **N)** Dual-luciferase assay to analyze the transfected promoters Fbxl4-1, Fbxl4-2, Fbxl4-3, Fbxl4-4, Fbxl4-4, and Fbxl4-5 in NMCMs in the NC group versus siSP1 group. n=3. **O)** Confirmation of SP1 plasmid efficacy in upregulating SP1 protein expression, n=4. **P)** Representative images and quantitative data of β-MHC protein levels of after co-transfection of SP1 and FBXL4 in Ang II-induced cardiomyocytes hypertrophy, n = 4. **Q)** ANP, BNP and β-MHC mRNA levels of after co-transfection of SP1 and FBXL4 in Ang II-induced cardiomyocytes hypertrophy, n=6. **R, S)** Representative images and quantitative data of PFN1 and FBXL4 co-transfection in Ang II-induced cardiomyocyte hypertrophy (at least 50 cells counted per experiment, n=7; green represents α-actinin; blue indicates nuclei；scale bar, 50 μm). n represents the number of independent samples per group. Data are shown as mean ± SD. **p* < 0.05, ***p* < 0.01, and ****p* < 0.001. *n.s* indicates no significance. Statistical differences were assessed by two-tailed unpaired Student’s *t* tests (B, C, D, E, F, G, H, I, J, K, N, O), one-way ANOVA with Tukey’s multiple comparisons test (M), two-way ANOVA followed by Sidak post hoc multiple comparisons test (P, Q, S).

**Reference**

[1] B. J. Wilkins, Y. S. Dai, O. F. Bueno, S. A. Parsons, J. Xu, D. M. Plank, F. Jones, T. R. Kimball, J. D. Molkentin, *Circ Res* **2004**, *94*, 110.

[2] X. Li, L. Zhang, T. Tian, Y. Pei, K. Wang, S. Wang, X. Ning, P. Zhao, Y. Qu, H. Gao, C. Li, X. Liu, J. Yang, Y. Zhang, H. Gao, L. Xuan, Y. Zhang, Y. Lu, B. Cai, B. Yang, Z. Pan, *Cell Death Differ* **2025**.

[3] A. Ibrahim, N. Yucel, B. Kim, Z. Arany, *Cell Metab* **2020**, *32*, 309.
